# Stimulus-Controlled Anion Binding and Transport by Synthetic Receptors

**DOI:** 10.1021/acs.chemrev.3c00039

**Published:** 2023-06-21

**Authors:** Jorn de Jong, Jasper E. Bos, Sander J. Wezenberg

**Affiliations:** Leiden Institute of Chemistry, Leiden University, Einsteinweg 55, 2333 CC Leiden, The Netherlands

## Abstract

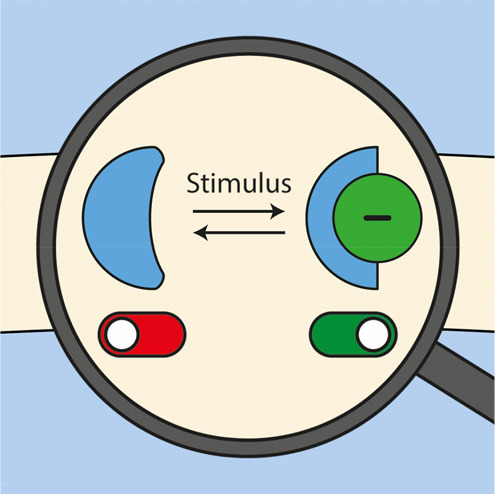

Anionic species are omnipresent and involved in many important biological processes. A large number of artificial anion receptors has therefore been developed. Some of these are capable of mediating transmembrane transport. However, where transport proteins can respond to stimuli in their surroundings, creation of synthetic receptors with stimuli-responsive functions poses a major challenge. Herein, we give a full overview of the stimulus-controlled anion receptors that have been developed thus far, including their application in membrane transport. In addition to their potential operation as membrane carriers, the use of anion recognition motifs in forming responsive membrane-spanning channels is discussed. With this review article, we intend to increase interest in transmembrane transport among scientists working on host–guest complexes and dynamic functional systems in order to stimulate further developments.

## 1 Introduction

Anions are ubiquitous throughout biological systems where they fulfill important roles, ranging from information and energy storage to osmotic regulation and signal transduction.^1,2^ Transport of anions across the plasma membrane is mediated by proteins, which are pivotal to many of our cellular functions. Defects in these proteins can cause serious illnesses of which the most well-known example is cystic fibrosis, but also the renal disease Bartter’s syndrome, and some forms of myotonia have been associated with faulty anion transport.^3^ Artificial receptors that are capable of recognizing and binding anions are therefore receiving major attention.^4−10^ Some of these receptors have been shown capable of facilitating transmembrane transport and therefore possess new modes of anticancer and antibacterial activity.^11−21^ In addition, they could one day be able to take over the function of malfunctioning transport proteins, thus offering new therapeutic treatment to faulty transport-related diseases. However, where the transport activity of membrane proteins is regulated in response to stimuli in the environment, current artificial anion receptors with transport capability typically exist in only a single (high-affinity) state. Controlling their transport properties, e.g., by altering the binding affinity using physiochemical stimuli would allow local activation at a pathological site (and deactivation elsewhere or when excreted) and hence, prevent undesired side effects as well as build-up of resistance in pharmacological treatment.^22−27^ Furthermore, stimuli-responsiveness will be a prerequisite toward the future development of transport systems that, like proteins, can operate fully autonomously in a biological environment.

Beside transmembrane transport, synthetic anion receptors have been applied in other key technologies, e.g., analyte sensing^28−30^ and wastewater treatment,^31,32^ and control of binding affinity would also be beneficial in these applications. Quantitative detection of analytes, for example, can suffer from background noise caused by other solutes, and selective extraction may overcome this problem.^33^ Further, extraction of contaminants from wastewater streams is important in avoiding environmental pollution. Where for such extractions to be successful a high binding affinity is desired, at the same time, it hampers recovery of the substrate and recyclability. This problem can be solved by switching the receptor between a high- and low-affinity state. Therefore, increasing effort is being devoted to the integration of stimuli-responsive functions into artificial anion receptors,^34−38^ which is still posing a major challenge as substrate binding can have a detrimental effect on the stimuli-responsive nature and multiple equilibria can be involved.

**Figure 1:**
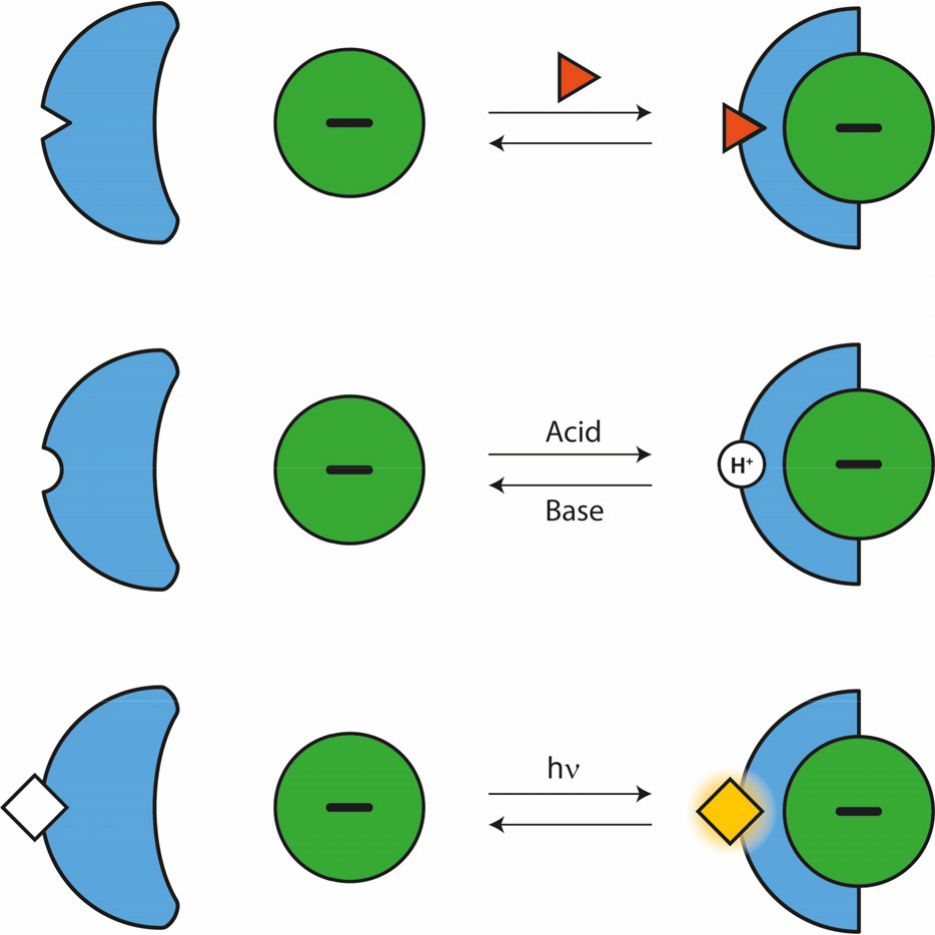
Approaches to stimuli-responsive anion receptors.

Within the field of host–guest chemistry, various stimuli have been applied to modulate the strength of noncovalent interactions that define binding affinity, among which are light^34,39−44^ and chemical or electrochemical stimuli.^45−49^ Where the use of light as exogenous stimulus is very convenient as it can be delivered with high spatiotemporal control and does not produce waste in the system,^22−27^ the use of an endogenous stimulus could be valuable when self-activation at a pathological site is desired. Tumor cells, for example, have a microenvironment with lower pH than normal cells as well as elevated concentrations of reducing agents (e.g., glutathione).^50−53^ What stimulus is preferred will thus largely depend on the envisioned application. Toward control of anion binding affinity, the use of light is by far dominant, and successful designs are mostly based on molecular photoswitches that are used as receptor backbone (Figure 1). In addition, allosteric modulators (in the form of metal cations) and pH change have been applied, whereas examples of redox control are scarce.

**Figure 2:**
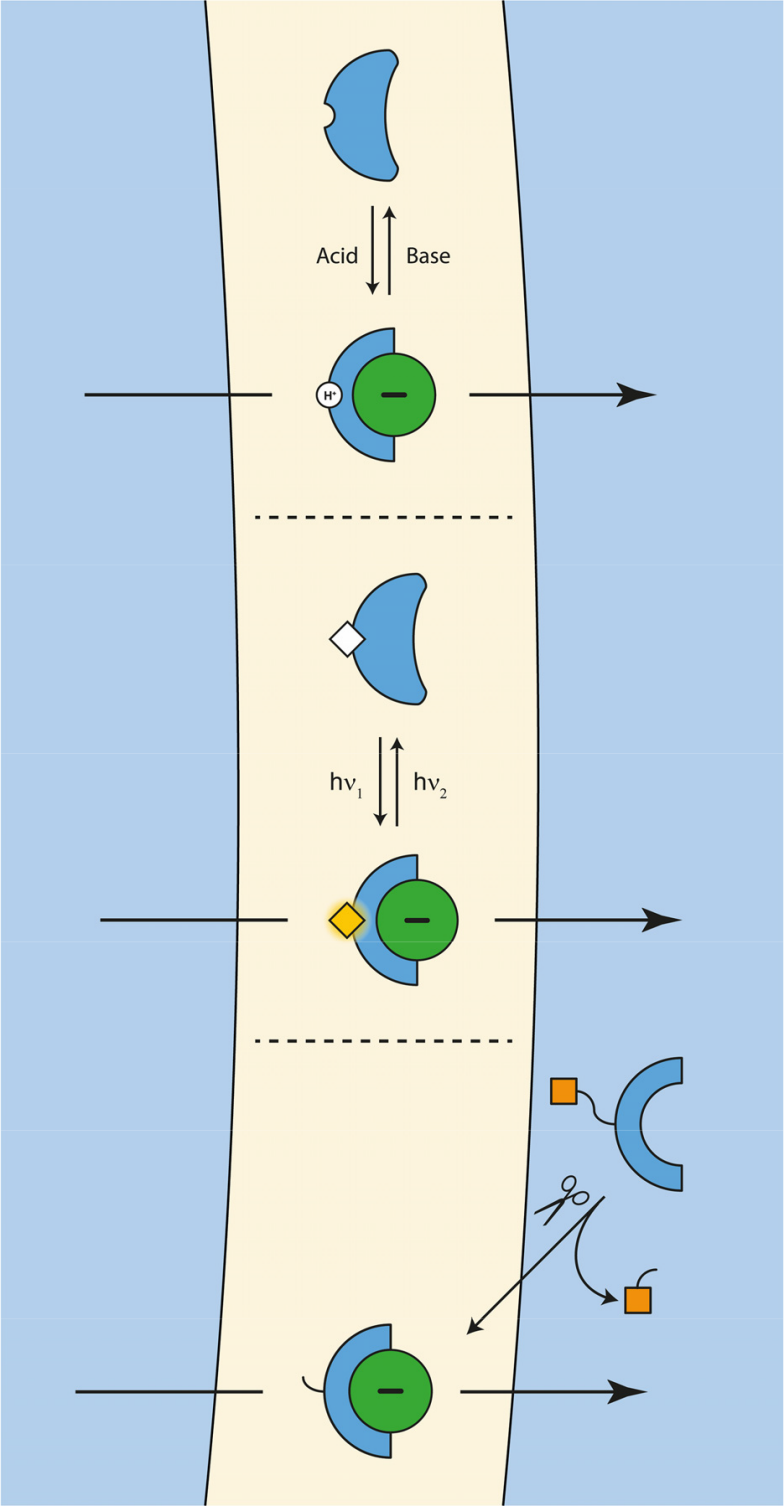
Most common approaches to control anion transport.

Interestingly, for the majority of stimulus-controlled anion receptors, no data with respect to transmembrane transport studies is reported, probably because the assays required to test them were unavailable to the laboratories where they were developed. As binding affinity has been related to transport activity, some of them may thus potentially act as switchable transporters, which is still to be explored. Although a significant number of articles that describe control of transport activity has appeared in recent years, most of them rely on strategies that are different than the ones used to control affinity, i.e., binding site (de)protonation and protective group cleavage to alter membrane partitioning (Figure 2). It thus seems that there is a gap to narrow between the development of responsive receptors and applications in transmembrane transport, which is what drove us to write this review article.

Herein, we give a fully comprehensive overview of the methods that have been developed to control binding affinity and transmembrane transport of anions. We start by highlighting the approaches that have been used to dynamically control binding properties and then continue with examples of responsive membrane carriers and channels. For the nonexpert, a brief description of commonly used assays to investigate transport behavior is included. While the last section describing anion-selective channels does not strictly fall under the term “receptors”, they often consist of similar anion recognition motifs and follow similar responsivity principles as the receptors and carriers that are discussed. Our aim is to identify which types of switchable anion binders have been, or can potentially be, successfully applied to facilitate transport.

## 2 Anion Capture and Release

### 2.1 Allosteric Control

The earliest systems investigated toward control of anion binding involve allosterism,^45−47,49^ as it is central to the working mechanism of various proteins. The binding of an allosteric effector in these systems can either result in stronger (positive allosterism) or weaker (negative allosterism) anion binding. Strategies developed so far are primarily based on complexation of (alkali) metal cations with ligands that are either directly connected to anion receptor motifs or to calixarene cavitands bearing such moieties, leading to a conformational change and repositioning of attached anion-binding substituents, i.e., amide or (thio)urea groups. Although in the examples discussed here the structural change is evident, contribution of ion-pairing interactions to enhance the overall binding affinity cannot always be excluded.

#### 2.1.1 Metal–Ligand Coordination

**Figure 3:**
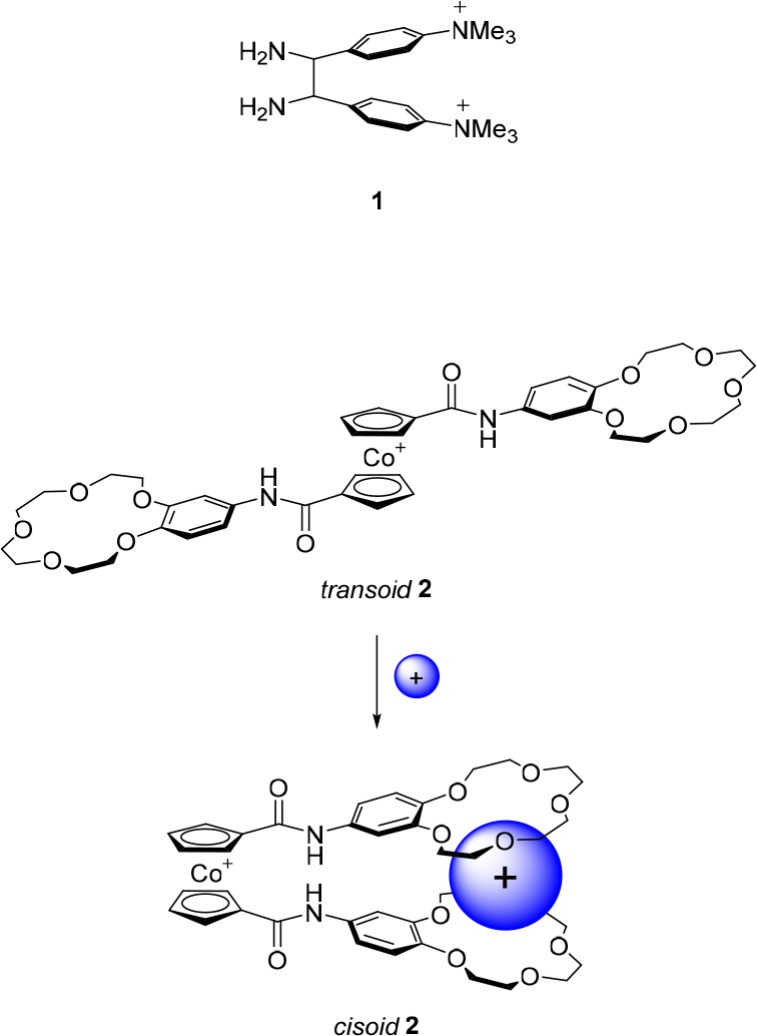
Allosterically controlled diphenylethane- and cobaltocenium-based receptors.

The first example of an allosterically controlled system that we encountered is the 1,2-diamino-1,2-diphenylethane-based receptor **1** with two *para*-trimethylammonium substituents, which was reported by the group of Schneider in 1992 (Figure 3).^54^ This receptor was shown to bind several dianionic guests in a *gauche* conformation with moderate strength via salt-bridge and π–π interactions in D_2_O (*K*_a_ = 10 M^–1^ – 27 M^–1^), as was determined by ^1^H NMR titrations. Coordination of Cd^2+^ ions to the amino-substituents induced a contracted form of the *gauche* conformer having a smaller torsional angle and Me_3_N^+^–Me_3_N^+^ distance, leading to an approximately 2-fold decrease in affinity for *ortho-*benzenedicarboxylate, *para-*benzenedicarboxylate, and *meta*-benzenedisulfonate.

A few years later, following a similar principle, Beer et al. developed cobaltocenium receptor **2** (Figure 3), which bears two benzamidocrown ether substituents.^55^ Upon complexation with alkali metal ions, the preferred conformation of this molecule was expected to change from *transoid* to *cisoid*, thereby bringing the two hydrogen-bond-donating amide groups closer together such that Cl^–^ and Br^–^ binding would be enhanced. ^1^H NMR studies in acetonitrile-*d*_3_ revealed that, in the absence of alkali metal cations, these halide anions had a binding affinity in the order of *K*_a_ ∼ 10^3^ M^–1^. Addition of Na^+^ did not exert significant influence on the binding strength, presumably because two of these ions are complexed by one receptor. Surprisingly, upon addition of the larger K^+^ ion, which was expected to bridge the crown ether moieties and thereby enhance anion binding affinity, complete release of the bound anions was observed. The authors postulated that anion binding is sterically restricted in the K^+^ complexation-induced and rigidified *cisoid* conformation, giving rise to negative allosterism.

**Figure 4:**
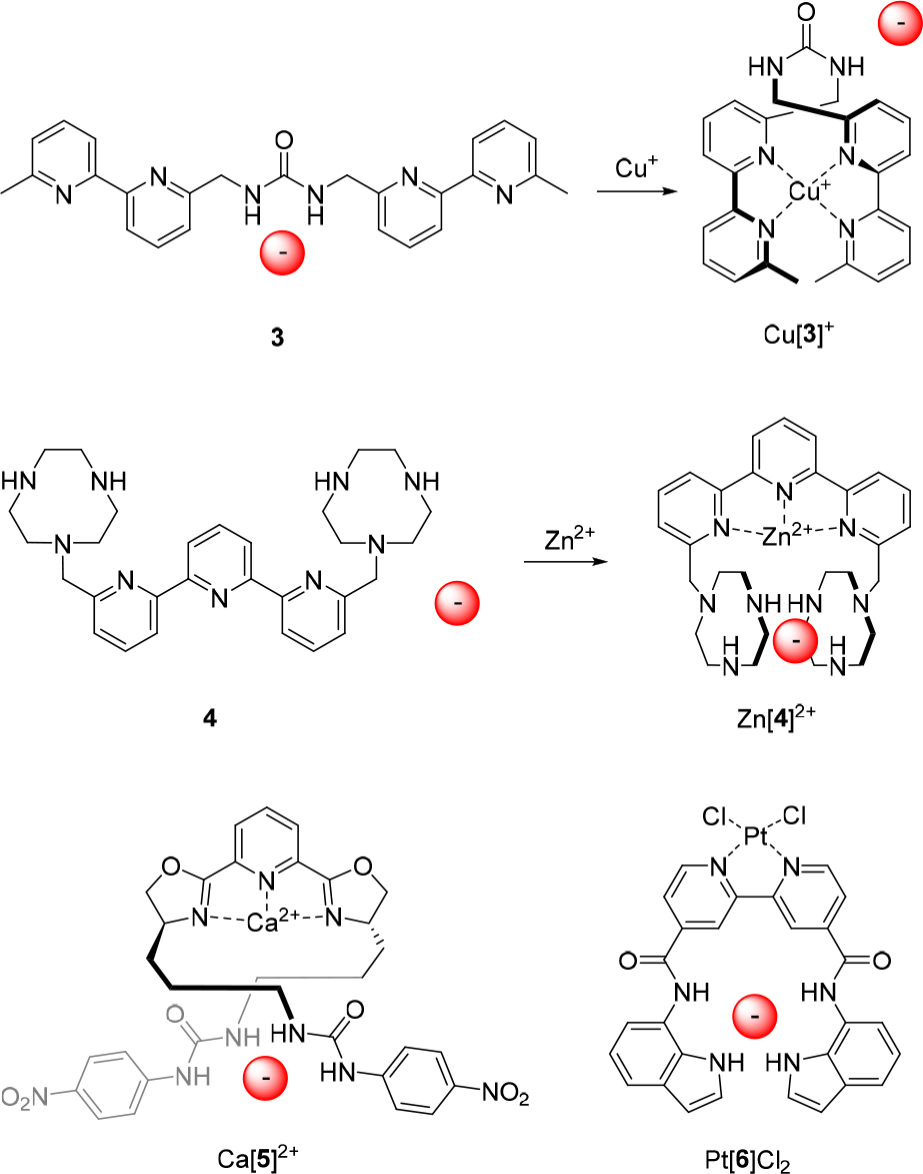
Metal cation-responsive allosteric anion receptors.

Toward the same goal, the group of Branda developed bipyridine-appended urea receptor **3** (Figure 4), which bound CH_3_CO_2_^–^ and *para*-methyl benzoate in DMSO-*d*_6_ with association constants of 50 M^–1^ and 55 M^–1^, respectively, as was determined by ^1^H NMR spectroscopy.^56^ Complexation of Cu^+^ by the two bipyridine substituents effectively restricted access to the urea binding site. Release of CH_3_CO_2_^–^ from the anion/receptor adduct was additionally demonstrated *in situ* by addition of one equivalent of [Cu(CH_3_CN)_4_](PF_6_). Unfortunately, attempts to reverse this process, for example, by subsequent addition of neocuproine as a Cu^+^ scavenger, were unsuccessful.

Where these previous designs displayed negative allosterism, a positive allosteric effect was accomplished by Bencini, Lippolis, and co-workers.^57^ They developed the diphosphate-selective receptor **4** in which the anion-binding [9]aneN_3_ units became preorganized for anion binding upon complexation of the central terpyridine unit with a metal cation, inducing a change from a *W*-shaped to a *U*-shaped conformation (Figure 4). At neutral pH, the receptor primarily existed as a diprotonated species, which displayed a binding constant for P_2_O_7_^4–^ of *K*_a_ = 7.9 × 10^2^ M^–1^. Remarkably, the binding affinity toward the [Zn(**4**)]^2+^ complex was approximately 11 000 times higher, with a *K*_a_ value of 8.9 × 10^6^ M^–1^. It may very well be that such a large increase is not only caused by a positive allosteric effect, but that ion-pairing or coordination of the anion to the metal cation plays an additional role. Nevertheless, in this case, the authors suggested on the basis of DFT calculations and potentiometric analysis that the P_2_O_7_^4–^ anion solely interacts via hydrogen bonding with the two protonated [9]aneN_3_ units, and that additionally two water molecules coordinate to the zinc center.

The group of Nabeshima designed receptor **5**, based on a 2,6-bis(oxazolinyl)pyridine (Pybox) ligand with two 4-nitrophenylurea substituents and able to undergo a comparable *W*-shaped to *U*-shaped conformational change upon metal complexation (Figure 4).^58^ The Pybox backbone is easier to functionalize than the terpyridine ligand and is chiral. UV–vis titrations in a mixture of acetonitrile/1%H_2_O revealed that the free receptor binds Cl^–^ with an affinity constant of *K*_a_ = 1.82 × 10^3^ M^–1^, whereas upon addition of Br^–^ and I^–^. no significant UV–vis spectral changes were observed. The [Ca(**5**)]^2+^ complex showed much larger binding affinities of *K*_a_ = 3.2 × 10^5^ M^–1^ for Br^–^ and I^–^ and of *K*_a_ = 1.0 × 10^6^ M^–1^ for Cl^–^, which corresponds to a 550-fold difference in binding strength between metal-complexed and uncomplexed states.

With the same objective, Caltagirone et al. developed the 4,4′-dicarboxamido-di(indol-7-yl)-2,2′-bipyrdine ligand **6** (Figure 4), of which the neutral Pt(II) chloride complex showed significantly higher binding affinity toward anions (in particular for H_2_PO_4_^–^) than its nonmetalated counterpart.^59^ That is, where the binding constant between the free receptor and the metal complex measured by ^1^H NMR titrations in DMSO-*d*_6_/0.5%H_2_O differed to around 3-to-4-fold for Cl^–^ and CH_3_CO_2_^–^ and 8-fold for benzoate, the affinity difference for H_2_PO_4_^–^ was approximately 40-fold with binding constants of *K*_a_ = 90 M^–1^ and *K*_a_ = 3644 M^–1^, respectively. Like in the previous examples, the higher binding affinity for the metal complex as compared to the free receptor can be ascribed to preorganization of the binding site.

#### 2.1.2 Functionalized Macrocycles

**Figure 5:**
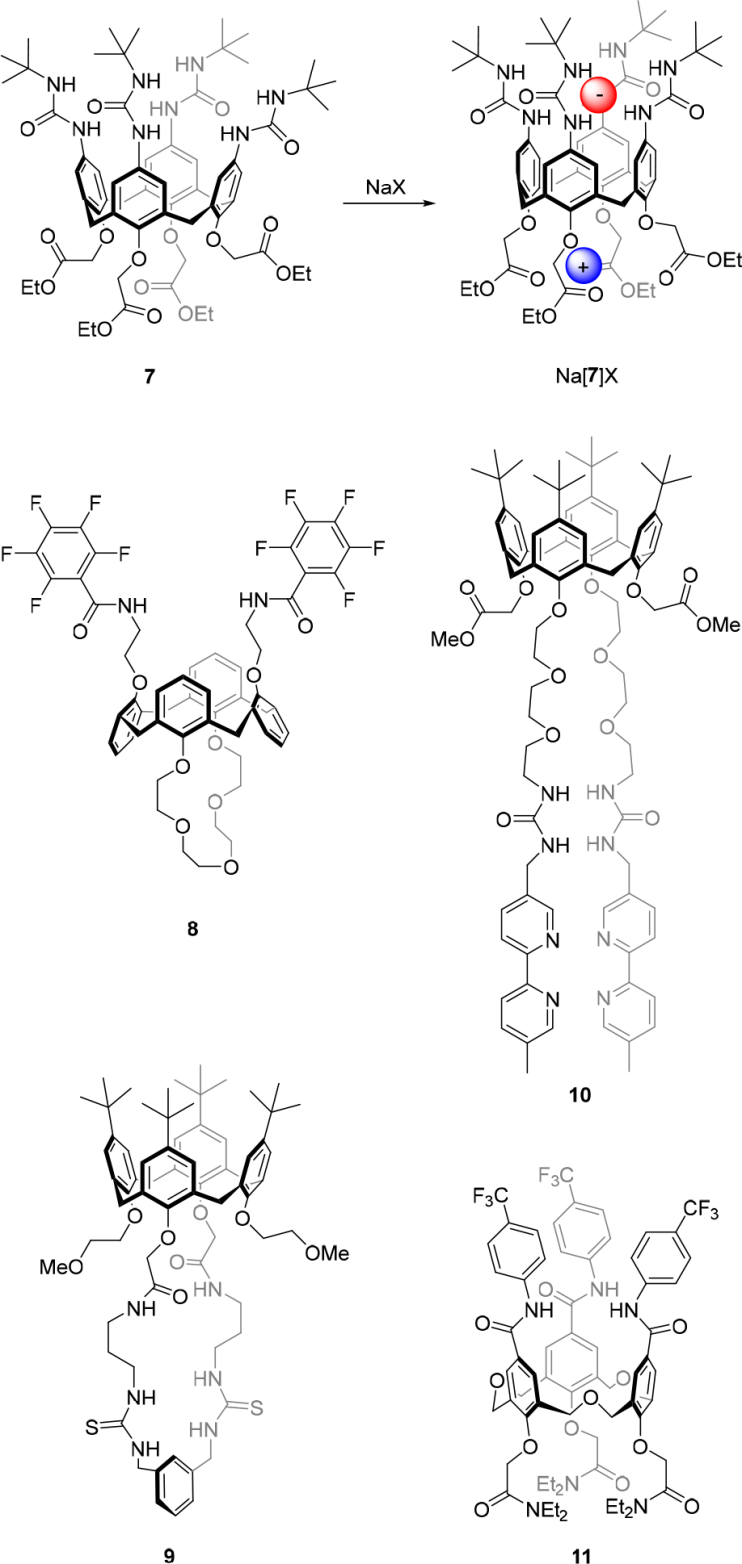
Calix[4]arene-based allosterically controlled anion receptors.

A more widely explored strategy toward allosteric control of anion binding is based on the use of calixarene scaffolds,^60^ which allow binding site preorganization upon interaction with (alkali) metal ions. In 1996, the group of Reinhoudt described calix[4]arenes functionalized with ester moieties on the lower rim and urea groups on the upper rim.^61^ The interaction of Na^+^ with the ester groups on the lower rim of **7** favored a more cylindrical shape instead of the regular cone conformation (Figure 5). In this cylindrical shape, the urea groups are brought closer together, and as a consequence, the association constant determined for the Na^+^ salts of Cl^–^, Br^–^, and I^–^ (e.g., *K*_a_ ≥ 10^4^ M^–1^ for Cl^–^ and *K*_a_ = 1.3 × 10^3^ M^–1^ for Br^–^ in chloroform) was larger than for the respective tetrabutylammonium (NBu_4_^+^) salts. Furthermore, the use of the K^+^ salts gave significantly weaker binding as compared to Na^+^, while in the case of the Cs^+^ salts, no binding was observed at all. This observation is in line with the order of the binding strength of these cations to ester moieties of **7**, which follows: Na^+^ > K^+^ > Cs^+^. Beside using different counter cations, initial treatment of the receptor with NaClO_4_ and subsequent addition of the NBu_4_Br or NBu_4_Cl salt resulted in strong anion binding.

**Figure 6:**
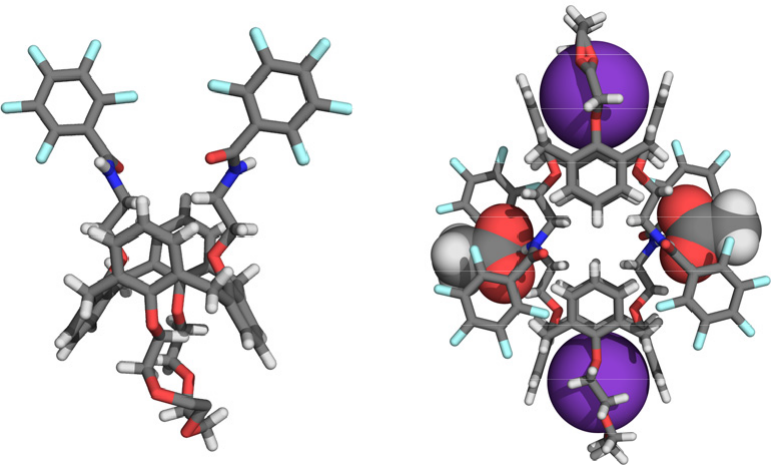
Single-crystal X-ray structures of unbound and bound **8**.

In a similar approach, Casnati, Ugozzoli, and co-workers used calix[4]arene **8** existing in a 1,3-alternate conformation, and having a crown ether bridge on one side and two pentafluorobenzamide residues on the other side (Figure 5).^62^ This compound was found to bind CH_3_CO_2_^–^ (added as NBu_4_^+^ salt) very weakly in chloroform-*d* (*K*_a_ = 35 M^–1^, as determined by ^1^H NMR spectroscopic titration). However, when one equivalent of K^+^ was added, the binding affinity for CH_3_CO_2_^–^ increased by a factor 4 to *K*_a_ = 140 M^–1^, which was ascribed to conformational changes induced by complexation of the crown ether with K^+^, although ion-pairing could also contribute to this enhancement. The authors obtained single crystals suitable for X-ray analysis of both the free receptor and the receptor complexed with KCH_3_CO_2_ (Figure 6). Interestingly, in the solid state, a 2:2:2 receptor/cation/anion complex was found for the latter. The anion-bound calix[4]arene showed a significantly different conformation than the free receptor, i.e., the amide groups were positioned in such a way that the CH_3_CO_2_^–^ anions could bridge between two units. Although this 2:2:2 complexation mode most likely does not correspond to the structure in solution, the solid-state analysis does suggest an enhanced stability of the anion/receptor complex upon complexation with the K^+^ ion, in line with the results from ^1^H NMR titration studies.

Alternatively, the group of Kilburn incorporated both the cation and anion binding site, which respectively consisted of phenoxyacetamide and cyclic bis-thiourea moieties, to the lower rim of calix[4]arene (compound **9**, Figure 5).^63^ Association constants were determined for CH_3_CO_2_^–^, phenylphosphinate, and diphenyl phosphate by ^1^H NMR titration experiments in acetonitrile-*d*_3_ as *K*_a_ = 1.1 × 10^4^ M^–1^, 2.4 × 10^4^ M^–1^, and 1.8 × 10^3^ M^–1^, respectively (by using the NBu_4_^+^ salts). In the presence of Na^+^, the binding affinity for diphenyl phosphate was slightly enhanced to a *K*_a_ value of 2.2 × 10^3^ M^–1^, but surprisingly, binding of CH_3_CO_2_^–^ and phenylphosphinate were negligible. It should be noted, however, that beyond the addition of one equivalent of CH_3_CO_2_^–^ the anion/receptor complex was observed, indicating that the first equivalent of CH_3_CO_2_^–^ precipitated as an ion-pair with Na^+^ to first afford the free receptor, which would then bind the excess CH_3_CO_2_^–^.

Nabeshima and co-workers added a third binding site to calix[4]arene to create the dual-responsive receptor **10** with four separately addressable states exhibiting distinct anion binding affinity (Figure 5).^64^ In their design, the lower rim was functionalized with two methyl acetate groups as well as with two polyether chains that were capped with urea-bipyridine. While the ester and polyether components were capable of binding Na^+^, the two bipyridyl units could complex Ag^+^. The free host displayed weak binding affinity toward NO_3_^–^ (*K*_a_ = 76 M^–1^) and CF_3_SO_3_^–^ (*K*_a_ = 25 M^–1^) in a 9:1 mixture of chloroform-*d*/acetonitrile-*d*_3_, whereas binding of BF_4_^–^ was negligible. The authors attributed this low affinity to the flexibility of the polyether chains (bearing the anion-binding urea groups), in addition to possible intramolecular urea hydrogen bonding. Remarkably, when either of the binary complexes was made using Na^+^ or Ag^+^, the binding affinity toward NO_3_^–^ and CF_3_SO_3_^–^ significantly increased (*K*_a_ ∼ 10^3^ to 10^4^ M^–1^). The ternary complex, formed upon addition of both Na^+^ and Ag^+^, showed an even larger increase in anion binding affinity, which was approximately 1500-fold for NO_3_^–^*(K*_a_ = 1.17 × 10^5^ M^–1^), 2000-fold for CF_3_SO_3_^–^ (*K*_a_ = 5.01 × 10^4^ M^–1^), and also the binding of BF_4_^–^ could now be quantified (*K*_a_ = 1.91 × 10^4^ M^–1^). Presumably, this dual metal cation-controlled increase in binding strength originates from both ion-pair interactions and a decrease in conformational flexibility.

The group of Yamato developed related receptor **11**, which was based on homooxacalix[3]arene instead of calix[4]arene and contained cation binding diethylacetamide substituents at the lower rim and anion-binding aromatic amides at the upper rim (Figure 5).^65^ Owing to preorganization of the binding site, in a similar way as described for the system designed by Reinhoudt and co-workers (*vide supra*), complexation with Li^+^ led to a 15- to 50-fold increase in binding affinity for Cl^–^ and Br^–^ as compared to the free receptor (which displayed association constants in the order of *K*_a_ ∼ 10^1^ to 10^3^ M^–1^ in 10:1 chloroform-*d*/acetonitrile-*d*_3_). In stark contrast, when Na^+^ was used, no significant binding of these anions was observed. The authors reasoned that upon complexation with Na^+^, the anion binding amide substituents are oriented in a more upright position. This positioning facilitates intramolecular hydrogen bonding between the N–H and C=O moieties, which impedes anion binding and has negative allosterism as a consequence.

**Figure 7:**
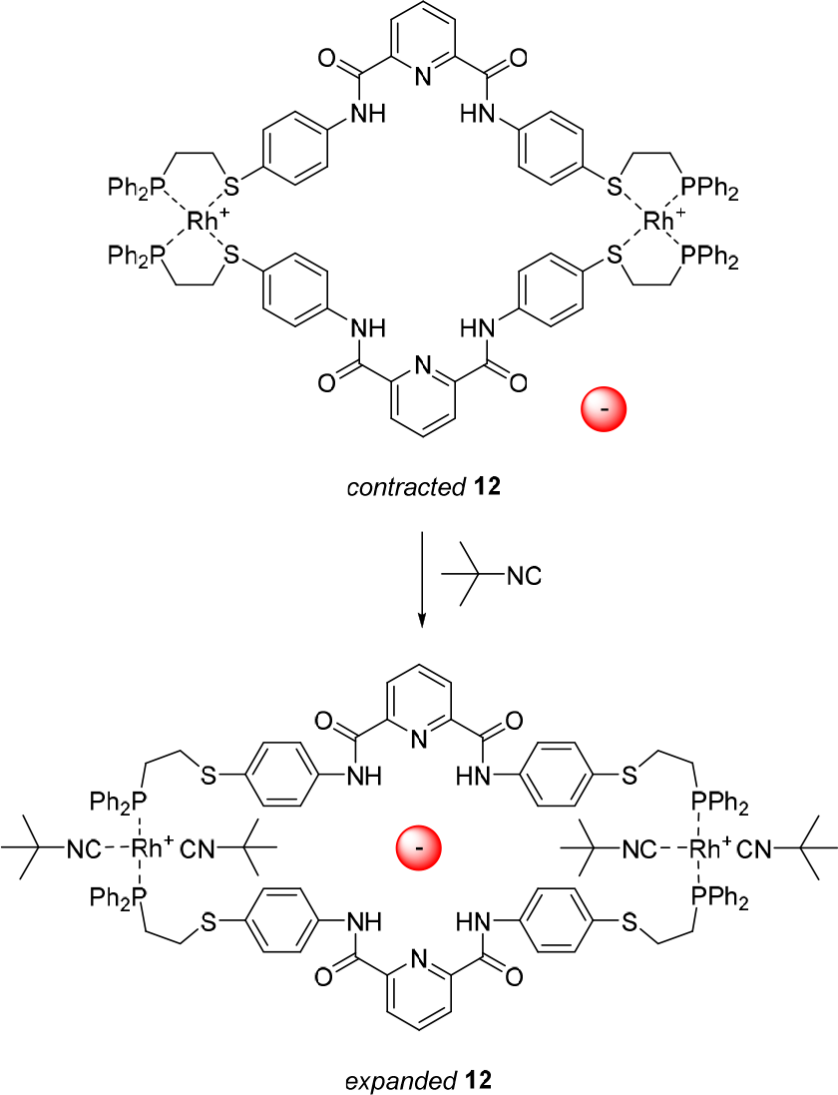
Allosterically controlled macrocycle expansion and contraction.

In contrast to the previous examples, which are all based on the complexation of (alkali) metal cations, Mirkin and co-workers reported a system in which the allosteric effector is a small organic molecule, i.e., *tert*-butylisocyanide.^66^ They developed the macrocyclic bis-rhodium complex **12**, consisting of two bridging hemilabile bis-phosphinothioether ligands bearing a 2,6-pyridinedicarboxamide anion binding motif (Figure 7). This macrocycle existed in a rigid contracted structure, which displayed a binding constant for Cl^–^ of *K*_a_ = 4.2 × 10^4^ M^–1^ in dichloromethane-*d*_2_ as was determined by ^1^H NMR spectroscopic titrations. The *tert*-butylisocyanide effector was able to replace the rhodium-coordinated sulfur atoms of the phosphinothioether ligands, leading to an enlargement and increase in flexibility of the macrocycle. Both these features caused enhanced Cl^–^ binding, as was reflected in a 60-fold increase in affinity constant up to *K*_a_ = 2.5 × 10^6^ M^–1^.

Although the anion binding strength is successfully altered by the allosteric effector in the examples discussed above, in most cases no information is provided with respect to reversibility of this process, and when discussed, it is often found to be irreversible. The multiple components needed to operate these systems seem to hamper reversibility and additionally, the use of cationic allosteric effectors may render the anion-bound complexes too stable to dissociate. This aspect makes it very challenging to find suitable applications, e.g., in biology, which is likely one of the key reasons why developments in this direction stalled in the past decade.

### 2.2 Acid/Base-Responsiveness

**Figure 8:**
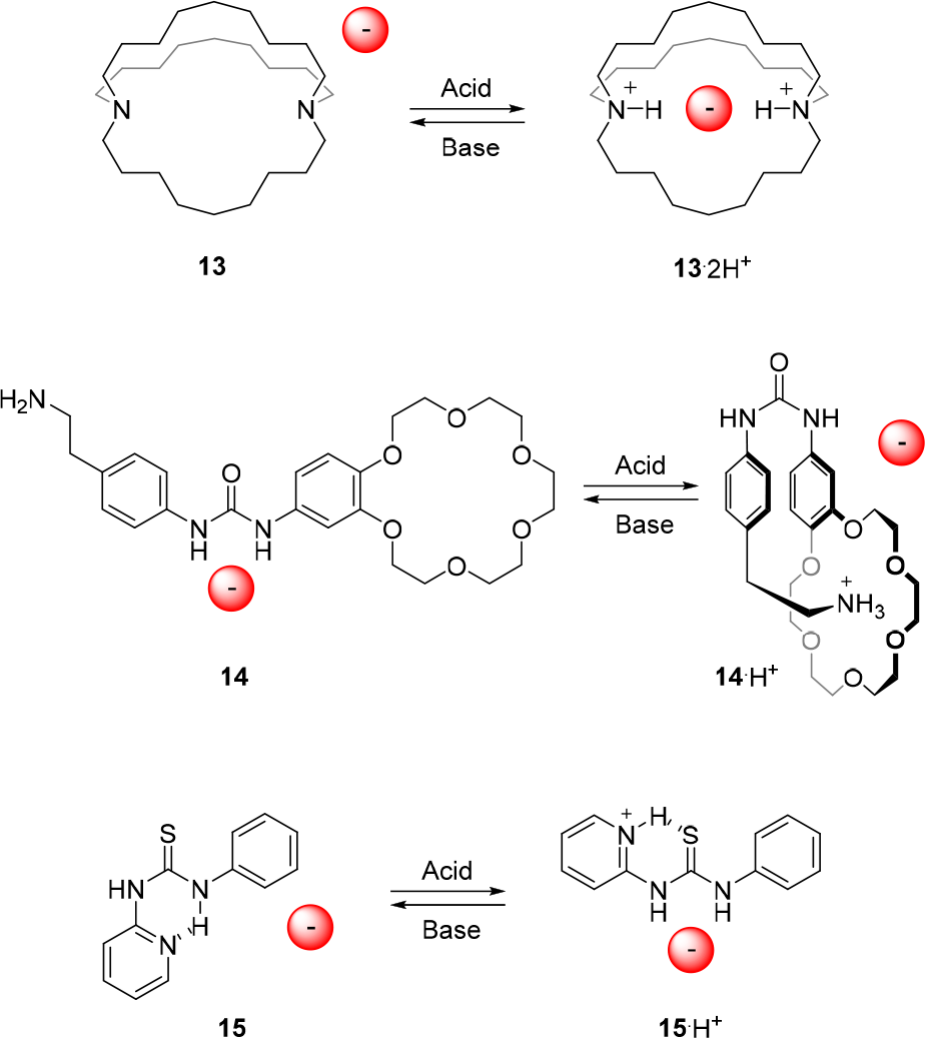
Acid/base-controlled anion binding to ammonium and (thio)urea receptors.

In general, anion receptors that are responsive to pH change show better reversibility than allosterically controlled ones. Importantly, these receptors could potentially respond to acidic pH in biological (tumor) microenvironments.^67^ For this reason, they are highly interesting for application in pH-controlled transmembrane transport, as is discussed in section 3. The first example of acid/base responsiveness we came across was reported by Park and Simmons in 1968.^68^ They observed that diazabicyclo[9.9.9]nonacosane (**13**) and diazabicyclo[10.10.10] dotriacontane encapsulated halide anions upon double protonation by TFA or HCl (Figure 8). This process was reversed by basifying the solution in order to recover the nonprotonated tertiary amines. In the years that followed, in particular, Lehn and co-workers developed various types of related (poly)cyclic amines and guanidines that bound anions in the protonated state, which can thus also be considered as pH-responsive receptors.^69−82^ However, because of the similarity with the example by Park and Simmons, they are not discussed in further detail here.

In 2002, the group of Branda reported an elegant design, which was based on a urea core with an 18-benzocrown-6 and a phenylethylamine substituent (compound **14**, Figure 8).^83^ Upon protonation of the amine functionality using HBF_4_, the resulting ammonium group could bind intramolecularly to the crown ether, thereby fixating the *cis*,*cis*-conformation of the urea moiety. This conformation has a lower binding affinity than the *trans*,*trans*-isomer, as was reflected in the results from ^1^H NMR titration studies. That is, where the neutral receptor showed an association constant for CH_3_CO_2_^–^ of *K*_a_ ∼ 4.4 × 10^3^ M^–1^ in DMSO-*d*_6_, the protonated receptor showed very weak affinity toward the same anion in the same solvent. Nevertheless, in the presence of 2–3 equiv of CH_3_CO_2_^–^, some binding to the protonated receptor could be observed. This means either that anion binding was able to compete with intramolecular ammonium-crown ether complexation to a certain degree or, perhaps, that the receptor was deprotonated by the added excess of acetate.

The group of Kilburn also described a strategy that is based on competitive intramolecular interactions by using pyridyl thiourea **15** (Figure 8).^84^ This receptor was shown highly effective in the acid/base-regulated binding of Cl^–^ and Br^–^. In its neutral form, it preferably exists in a *trans*,*cis*-conformation, which is stabilized by an intramolecular hydrogen bond between the thiourea N–H and the pyridyl N atom. In this conformation anion binding is blocked, but when the pyridyl N atom is protonated, for example using HBF_4_, the now protonated pyridyl ring will hydrogen bond to the thiourea S atom. The latter results in stabilization of the *trans*,*trans*-conformation, and exposes the thiourea binding site. In acetonitrile-*d*_3_, binding constants larger than *K*_a_ = 10^4^ M^–1^ were found for 1:1 complexation of both Cl^–^ and Br^–^ with the protonated receptor.

**Figure 9:**
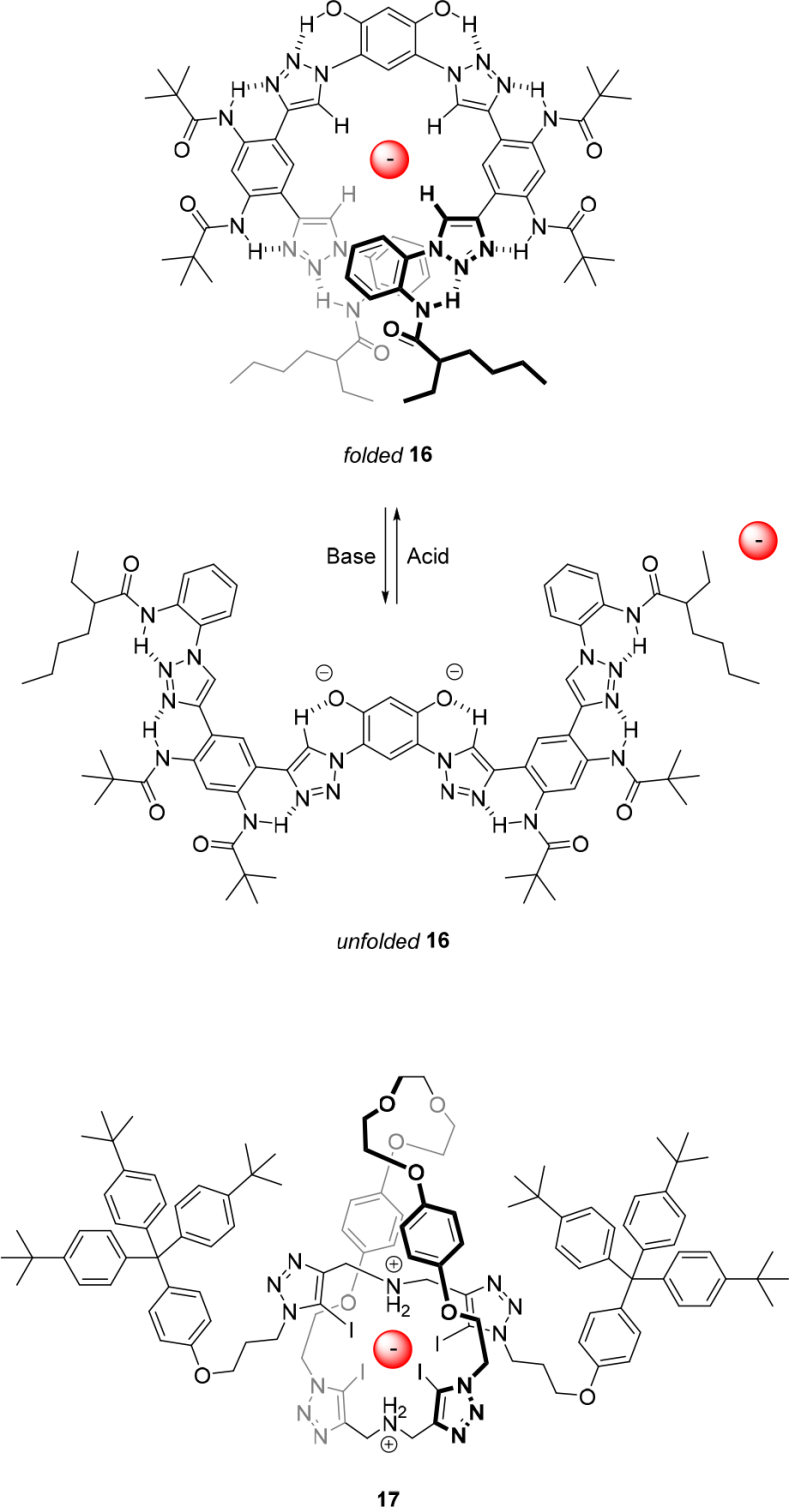
Acid/base-controlled binding to a foldamer and mechanically interlocked molecule.

In another approach described by Zhao et al., halide-selective aryl-triazole foldamer **16** was used (Figure 9).^85^ In this foldamer, the arylhydroxy groups are able to hydrogen bond to the neighboring triazoles, which leads to stabilization of a helical conformation with a cavity suitable for anion binding. Deprotonation of the arylhydroxy groups using DBU led to disruption of the hydrogen bonding interactions to afford a mixture of a monoanionic *S*-shaped and a dianionic *W*-shaped conformer. The latter form exhibited significantly lower anion binding affinity due to the larger distance between the triazole rings as well as electrostatic repulsion. Importantly, neutralization of the solution by addition of picric acid completely reversed this process. An impressively large difference in binding affinity between the neutral and deprotonated state (260-fold for Cl^–^, 67-fold for Br^–^ and 15-fold for I^–^) was found by ^1^H NMR titrations in a 94:6 mixture of chloroform-*d*/DMSO-*d*_*6*_. Furthermore, in the more competitive 85:15 mixture of chloroform-*d*/DMSO-*d*_*6*_ binding to the deprotonated form was negligible, while some binding to the neutral form was maintained.

The group of Beer developed various anion-binding mechanically interlocked structures,^86−88^ and in one of their halogen-bonding [2]rotaxanes, binding affinity could be controlled by protonation.^89^ In its neutral form, [2]rotaxane **17** (Figure 9) displayed minor affinity for Cl^–^ (in a mixture of chloroform-*d*/methanol-*d*_4_ (1:1 v/v)) and for NO_3_^–^ (in acetone-*d*_6_). However, upon protonation of the amine groups in both the macrocycle and the axle using HBF_4_, the iodotriazole units adopted an *endo* conformation facilitating anion–halogen bonding and hence, the proton may be regarded as an orthosteric effector. In the protonated form, the binding affinity for Cl^–^ and NO_3_^–^ was determined by ^1^H NMR titrations as *K*_a_ = 8.1 × 10^2^ M^–1^ and 5.8 × 10^2^ M^–1^, respectively, in a 45:45:10 mixture of chloroform-*d*/methanol-*d*_4_/D_2_O.

Where in this case, somewhat similar to the first example by Park and Simmons described above, protonation is used to create the anion binding site, in the other approaches, it mainly influences intramolecular (electrostatic) interactions. By changing these interactions, the anion binding site can either be blocked or liberated, which is a strategy that has proven successful in the control of anion binding affinity. On the downside, sequential addition of acid/base will generate chemical waste in the system, which may impede prolonged operation.

### 2.3 Switching by Light

The most widely investigated method to reversibly control anion binding affinity is based on the use of photoswitchable scaffolds.^34−36^ Light is a very convenient external stimulus to apply because of its high spatiotemporal precision and noninvasiveness, i.e., no chemical waste is generated in the system. In most of the approaches developed, two isomers of the receptor are interconverted by light of different wavelength, and the receptor design is chosen in such a way that one of the photoaddressable isomers exhibits strong affinity, whereas the other one binds more weakly. A relatively simple strategy to switch binding affinity is based on molecular tweezers,^90^ yet foldamers and macrocycles have also been used. In this section, information on the photoswitching properties is given as far as it has been detailed in the original publication.

#### 2.3.1 Stilbene-Based Tweezers

In the molecular tweezer approach,^91^ a photoswitch is equipped with a pair of anion-binding motifs, being far apart from each other in one state and brought together in the other state. The latter may position them in a way that permits simultaneous substrate binding leading to increased affinity. The use of stiff-stilbene (1,1′-biindanylidene) as the photoswitchable core has proven very successful, owing to its rigidity and large geometrical change upon isomerization.^92,93^ Furthermore, its thermal isomerization barrier is very high,^94^ allowing to synthesize/isolate each isomer and to study its binding properties separately.

**Figure 10:**
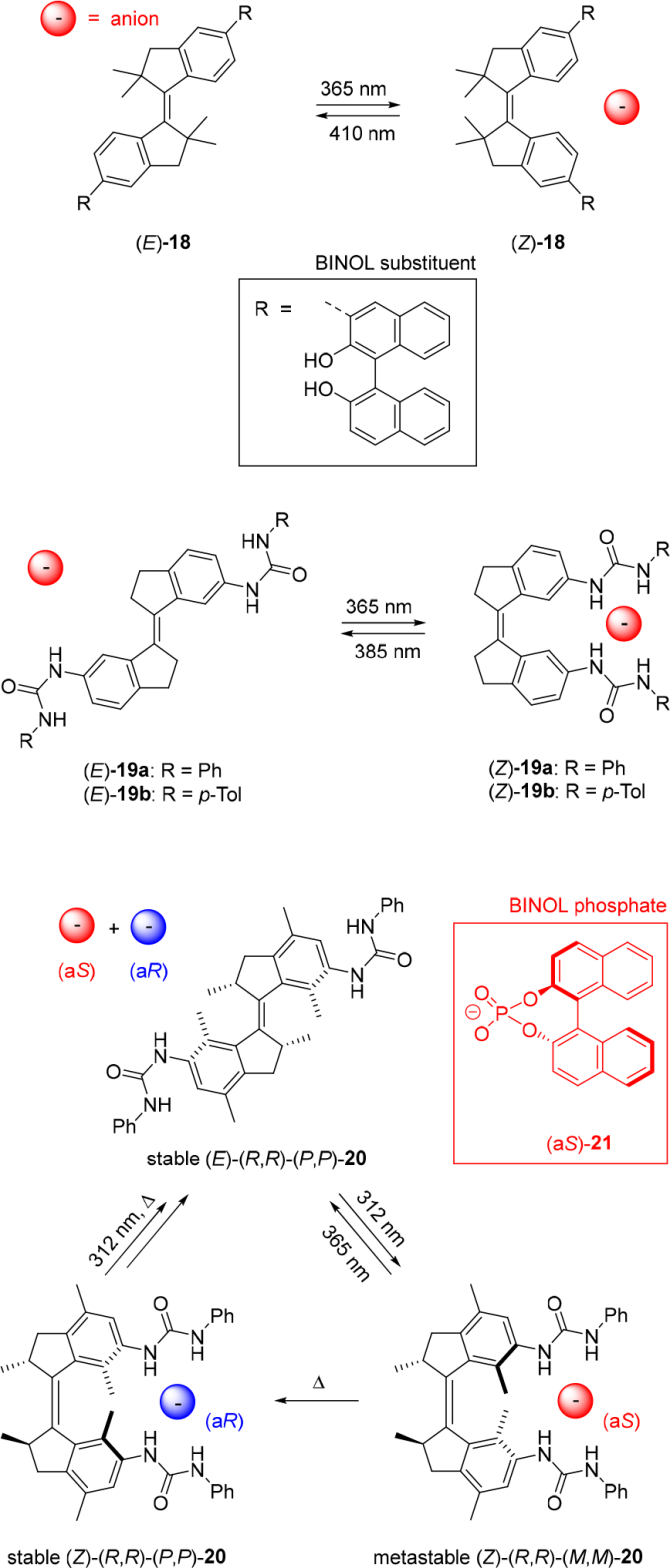
Photoswitchable anion receptors based on stiff-stilbene.

Shimasaki et al. explored this concept by functionalizing stiff-stilbene with two 2,2′-dihydroxy-1,1′-binaphthyl (BINOL) moieties (compound **18**, Figure 10).^95^ Irradiation of the *E*-isomer in benzene using 365 nm light gave a photostationary state (PSS) mixture with a 14:86 (*E*/*Z*) ratio. The reverse isomerization process could be induced with 410 nm light, resulting in a PSS ratio of 77:23 (*E*/*Z*). Examination of the anion binding properties by ^1^H NMR titration with F^–^, Cl^–^, and H_2_PO_4_^–^ in chloroform-*d* unexpectedly showed that, where the binding affinity of both isomers for F^–^ was similar, binding of Cl^–^ to *E*-**19** was 8 times stronger (*K*_a_ = 4.6 × 10^2^ M^–1^) than to *Z*-**19** (*K*_a_ = 59 M^–1^). Unfortunately, the titration data obtained for H_2_PO_4_^–^ and the *E*-receptor could not be fitted to a suitable binding model, implying a multistep equilibrium, whereas the binding strength of this anion to the *Z*-isomer was relatively weak (*K*_a_ = 94 M^–1^). The authors did reason that the H_2_PO_4_^–^ anion could interact with *Z*-**19** through both BINOL substituents and hence, four simultaneous OH-anion hydrogen bonds. However, the cavity surrounded by the four OH hydrogen bond donors was calculated to span 7–8 Å, which is larger than what would be required for simultaneous binding and might offer an explanation for the weak binding.

Our group developed photoresponsive anion receptor **19a** by attachment of the well-known urea anion-binding motif^96−98^ to stiff-stilbene (Figure 10).^99^ Here, irradiation of the *E*-isomer with 365 nm light gave a PSS mixture with a 51:49 (*E*/*Z*) ratio, and irradiation of the *Z*-isomer with 385 nm light resulted in a PSS ratio of 93:7 (*E*/*Z*). ^1^H NMR titration experiments, performed in DMSO-*d*_6_/0.5%H_2_O, showed much stronger anion binding to the *Z*-isomer than to the *E*-isomer, in particular for CH_3_CO_2_^–^ (*K*_a(*Z*)_ = 1.40 × 10^3^ M^–1^ and *K*_a(*E*)_ = 1.04 × 10^2^ M^–1^) and H_2_PO_4_^–^ (*K*_a(*Z*)_ = 2.02 × 10^3^ M^–1^ and *K*_a(*E*)_ = 77 M^–1^). The stronger binding to *Z*-**19a** is in line with the expected simultaneous binding of both urea substituents to a single anion via four hydrogen bonds, as was supported by DFT calculations. For the *E*-isomer, this simultaneous binding is not possible due to geometric constraints, and hence, the anion can be bound only by one urea group via two hydrogen bonds leading to different binding stoichiometry (2:1 vs 1:1 complexation) and much lower affinity.

In a subsequent study, in collaboration with the group of Beves, we demonstrated control over diffusion of the bis-tolylurea derivative **19b** by light in the presence of oligomeric H_2_PO_4_^–^ in DMSO/0.5%H_2_O.^100^ Tolyl substituents were installed to facilitate diffusion NMR measurements using the distinctive CH_3_-signal. The diffusion coefficient of (*E*)-**19b** was slightly lower (∼7%) than that of (*Z*)-**19b** because of its more extended structure. Interestingly, in the presence of H_2_PO_4_^–^, an overall 2–3-fold decrease in the diffusion constant of both isomers was noted. This decrease was ascribed to the formation of large self-assembled structures containing multiple receptors and H_2_PO_4_^–^ oligomers, which were found to exist at millimolar concentration due to antielectrostatic hydrogen bonding. Importantly, the overall decrease in diffusion constant was larger with (*E*)-**19b** than with (*Z*)-**19b**, indicating the formation of larger self-assembled structures with the *E*-isomer. As a result, light-triggered *E*–*Z* isomerization in the presence of H_2_PO_4_^–^ led to a larger change in diffusion constant (∼16%) than in its absence (∼7%), where the difference in effective volume of the assemblies was estimated as 70%.

Later on, our group described an additional set of bis(thio)urea derivatives (compounds **22a**, **76c**, and **76d**), which were tested in membrane transport assays in collaboration with the group of Gale (see section 3.3).^101^ In addition, Song and co-workers incorporated a phenyl linker between the stiff-stilbene core and the urea substituents, giving similar photoswitching and binding properties.^102^

Where all other examples described here are based on bistable switches, in 2014, we reported the bis-urea receptor **20**, which is derived from a molecular motor^103−106^ and could be switched by light and heat between three states with different anion binding affinity (Figure 10).^107^ Irradiation of stable (*E*)-**20** with 312 nm light induced the formation of metastable (*Z*)-**20**, resulting in a 20:80 (*E*/*Z*) PSS ratio. Heating of this PSS_312_ mixture allowed quantitative conversion of metastable (*Z*)-**20** to stable (*Z*)-**20** while, alternatively, irradiation with 365 nm light gave nearly full conversion back to stable (*E*)-**20**. Furthermore, 312 nm irradiation of stable (*Z*)-**20** directly afforded the 20:80 stable (*E*)-**20**/metastable (*Z*)-**20** PSS_312_ mixture. ^1^H NMR titrations in DMSO-*d*_6_/0.5%H_2_O revealed strong and selective binding of H_2_PO_4_^–^ to the stable *Z*-isomer (*K*_a_ = 7.5 × 10^3^ M^–1^), while binding of this anion to the stable *E*-isomer was almost 60 times weaker (*K*_a_ = 1.3 × 10^2^ M^–1^). Interestingly, the metastable *Z*-isomer also had a slightly weaker binding affinity toward H_2_PO_4_^–^ (*K*_a_ = 2.3 × 10^3^ M^–1^). A possible explanation for this came from DFT calculations, which revealed a larger dihedral angle around the central double bond for the metastable *Z*-isomer with respect to the stable one. Consequently, the urea groups must be further apart in the former isomer, presumably causing the weaker H_2_PO_4_^–^ binding. Finally, multistate control of the fraction of bound H_2_PO_4_^–^ was demonstrated *in situ* over several cycles using ^31^P NMR spectroscopy. For a 1:1 anion/receptor mixture (1 mM in DMSO-*d*_6_), it was calculated that this fraction varied between 20%, 48%, and 61% by starting with the *E*-isomer, irradiating the sample with 312 nm light, heating at 60 °C, and then irradiating consecutively with 312 and 365 nm light.

**Figure 11:**
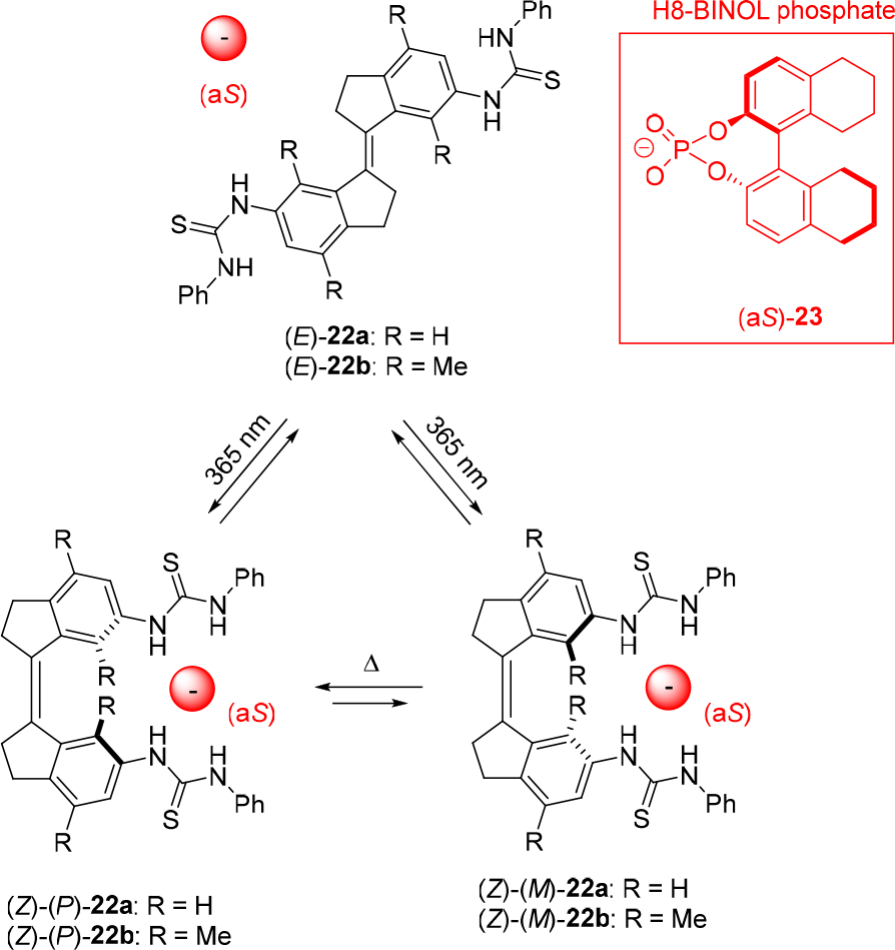
Supramolecularly directed rotational motion in stiff-stilbene.

The photochemical and thermal isomerization steps of **20** are accompanied by an inversion of helical chirality. Where in the previous study the racemate was used, by synthesizing the optically pure isomers, we later demonstrated that the stable and metastable *Z*-isomers of this bis-urea receptor exhibit opposite enantioselectivity for chiral BINOL phosphate anions (Figure 11).^108^ That is, ^1^H NMR titration experiments in DMSO-*d*_6_/0.5%H_2_O revealed that (a*R*)-BINOL phosphate is more strongly bound by stable (*Z*)-(*R*,*R*)-(*P*,*P*)-**20** (*K*_a_ = 415 M^–1^) than its (a*S*)-counterpart (*K*_a_ = 100 M^–1^). The opposite trend was observed for metastable (*Z*)-(*R*,*R*)-(*M*,*M*)-**20**, generated upon irradiation with 312 nm light, which formed a more stable complex with (a*S*)-BINOL phosphate (*K*_a_ = 55 M^–1^) than with (a*R*)-BINOL phosphate (*K*_a_ = 17 M^–1^). As expected, the stable *E*-isomer showed much weaker binding and no enantioselectivity. A similar concept was later reported by Feringa and Qu to invert enantioselective binding of dialkylammonium guests to molecular motor-based crown ether macrocycles.^109^ However, to the best of our knowledge, the system described here still represents the only example in which stereoselectivity toward an anionic substrate is controlled in a dynamic fashion.

While not exploited in these examples, the molecular motor core in **20** undergoes unidirectional rotational motion, which is directed by the chiral methyl substituent (Figure 10).^103−106^ Recently, we demonstrated that such motion can be induced in achiral stiff-stilbene bis-thiourea receptor **22a** via complexation with a chiral phosphate guest (Figure 11).^110^ Where the *E*-isomer of stiff-stilbene is virtually planar, the *Z*-isomer exists in *P* and *M* helical forms. The energy barrier for interconversion between these helical forms was calculated by DFT as 16.7 kJ mol^–1^ at room temperature, meaning that this process is very fast and that the *Z*-isomer will thus exist as a racemic *P*/*M* mixture. Nevertheless, the addition of enantiopure (a*S*)-H8-BINOL phosphate to (*Z*)-**22a** in dichloromethane induced a preference for one of the helical forms, as was demonstrated by the emergence of a positive absorption band in the circular dichroism (CD) spectrum. The exact opposite CD signal was observed when (a*R*)-H8-BINOL phosphate was added, confirming helical chirality induction. Furthermore, in the presence of the optically pure (a*S*)-phosphate guest, the ^1^H NMR spectrum recorded at −55 °C showed two distinct sets of signals (in a ratio of approximately 10:1) that were ascribed to the (*Z*)-(*P*)-**22a** ⊂ (a*S*)-**23** and (*Z*)-(*M*)-**22a** ⊂ (a*S*)-**23** diastereomeric complexes. DFT calculations indicated that the former complex is the lowest in energy by 5.6 kJ mol^–1^ and that the positive CD signal must therefore originate from the (*P*)-helical isomer.

Now, when a sample containing (*E*)-**22a** and one of the H8-BINOL enantiomers is irradiated with 365 nm light, (*Z*)-(*P*)-**22a** and (*Z*)-(*M*)-**22a** will be formed with equal probability. However, complexation of the photogenerated *Z*-isomer with the chiral H8-BINOL guest will lead to preferential formation of one of the helical isomers. Simultaneously, back isomerization to the *E*-isomer will occur (with the same rate as the forward process once the PSS_365_ is reached) as both (*E*)-**22a** and (*Z*)-**22a** absorb at the irradiation wavelength. This *Z* → *E* back isomerization will then predominantly take place from the preferentially formed helical isomer, resulting in a net unidirectional rotation over the central double bond. This work showed for the first time that unidirectional rotary motion can be directed via noncovalent binding of a chiral substrate, while in earlier molecular motor designs intrinsic chirality or specific sequences of reaction steps were always needed.^105,111^

To exclude that enantioenrichment would take place already in the photochemical step, we recently prepared the xylene analogue **22b** of this receptor (Figure 11), which has increased steric crowding and, with that, a higher helix inversion barrier.^112^ This time, the preferred helix formation upon addition of chiral guest could be monitored over the course of multiple hours at room temperature by, e.g., ^1^H NMR and CD spectroscopy. The addition of (a*S*)-H8-BINOL phosphate to a racemic mixture of the *Z*-isomer in dichloromethane eventually gave (*Z*)-(*P*) ⊂ (a*S*) and (*Z*)-(*M*) ⊂ (a*S*) in a 1.46:1 ratio. Here, when (*E*)-**22b** was irradiated with 312 nm light in the presence of (a*S*)-H8-BINOL, 40% of (*Z*)-**22b** was generated. Importantly, the hence formed (*Z*)-(*P*)-**22b** ⊂ (a*S*)-**23** and (*Z*)-(*M*)-**22b** ⊂ (a*S*)-**23** diastereomeric complexes were present in a 1:1 ratio immediately after the PSS_312_ was reached, thus excluding enantioenrichment during the photochemical step and assuring unidirectionality during the proposed rotary cycle.

**Figure 12:**
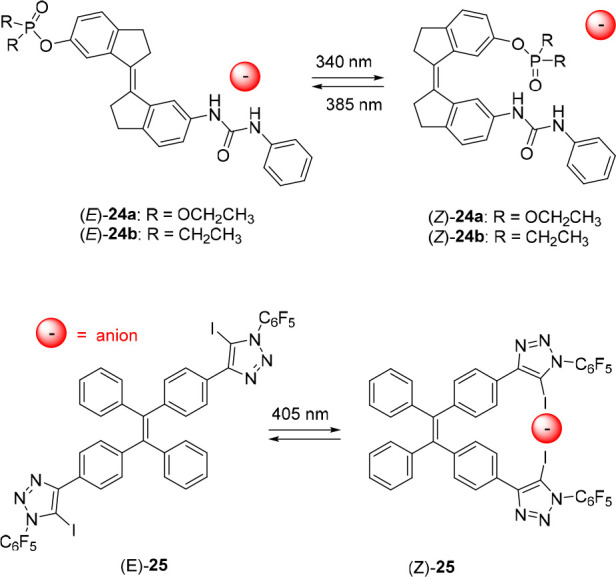
Other receptors based on light-induced C=C bond isomerization.

In the aforementioned cases, the low-affinity forms still binds anions to a certain extent. In an attempt to fully block binding in one of the states, we functionalized the stiff-stilbene scaffold with a urea group on one side and a complementary hydrogen bond accepting diethylphosphate or -phosphinate group on the other side (compounds **24a** and **24b**, Figure 12).^113^ While thus not strictly a molecular tweezer approach, this work is strongly related to the other stilbene-based examples and therefore discussed in this section. Irradiation of (*E*)-**24a** and (*E*)-**24b** with 340 nm light resulted in the formation of PSS mixtures with *E*/*Z*-ratios of 47:53 or 42:58, respectively. The isomerization process could be reversed with 385 nm light, giving PSS ratios (*E*/*Z*) of 94:6 for **24a** and 93:7 for **24b**. Although, as anticipated, intramolecular hydrogen bonding in the *Z*-isomer was found to lower the binding affinity with respect to the *E*-isomer, the difference was moderate (up to 2.5-fold for CH_3_CO_2_^–^, H_2_PO_4_^–^, and Cl^–^ in DMSO-*d*_6_/0.5%H_2_O). Importantly, compound **24b** with the stronger hydrogen bond accepting diethylphosphinate group did show a larger difference in binding strength than its diethylphosphate counterpart **24a**, corroborating the competitive effect of intramolecular hydrogen bonding.

Beer, Langton, and co-workers reported tetraphenylethene-based receptor **25** appended with two halogen-bonding aryl-iodotriazole motifs (Figure 12).^114^ Irradiation of either isomer with 405 nm light was shown to result in a 48:52 (*E*/*Z*) mixture, however, most likely due to poor absorption band separation of (*E*)-**25** and (*Z*)-**25**, no reversible switching was demonstrated. Nevertheless, in the presence of 10 equiv of halide anion, the PSS_405_ ratio of the *Z*-isomer to *E*-isomer increased (up to 32:68 *E*/*Z* in the case of Cl^–^) and correlated with the binding strength Cl^–^ > Br^–^ > I^–^. Titration studies by ^1^H NMR spectroscopy in tetrahydrofuran-*d*_8_ illustrated that halide binding to the *Z*-isomer is stronger than to the *E*-isomer (e.g., for Cl^–^*K*_a,1:1(*Z*)_ = 2.3 × 10^4^ M^–1^ and *K*_a,1:1(*E*)_ = 5.0 × 10^3^ M^–1^), which led to the conclusion that the bias toward the *Z*-isomer is based on the enhanced binding affinity.

Prior to this study, our group also observed that the amount of *Z*-isomer in the PSS mixture of stiff-stilbene bis-urea **19a** and bis-thiourea **22a** (Figures 10 and 11) is elevated in the presence of CH_3_CO_2_^–^ and H8-Binol phosphate, respectively.^99,110^ In the latter case, the PSS_365_ (*E/Z*) ratio in the absence of anionic guest was determined by ^1^H NMR spectroscopy as 42:58 and in its presence as 24:76. Photoisomerization quantum yield determination showed no significant influence on the forward *E* → *Z* isomerization process (Φ_*E*→*Z*_ = 18.2%) by the presence of guest (Φ_*E*→*Z*_ = 20.1%). Because the molar absorptivities (ε) at the irradiation wavelength were roughly the same for bound and unbound species, it indicated that guest binding lowers the quantum yield of backward *E* → *Z* isomerization [note that PSS (*E/Z*) = Φ_*Z*→*E*_ ε_Z_/Φ_*E*→*Z*_ ε_E_]. However, further investigation is needed to fully explain these effects. In summary, the use of stiff-stilbene has proven very successful toward the development of photoswitchable anion receptors. A drawback for biological application, however, is that they are operated by harmful UV light and hence, methods to operate them with visible light are being developed.^115^

#### 2.3.2 Azobenzene-Based Tweezers

Azobenzene derivatives^116−118^ are by far the most widely used molecular photoswitches in the development of photoresponsive receptors. Their synthesis and functionalization is straightforward compared to stiff-stilbene, and various strategies to red-shift their excitation wavelength have already been developed.^119,120^ On the other hand, azobenzene is not as rigid as stiff-stilbene and usually reverts back to its *E*-isomer in the dark at room temperature, which makes analysis of the properties of the photogenerated *Z*-isomer more challenging. Therefore, average binding constants of the PSS mixtures are given in most of the examples discussed below.

**Figure 13:**
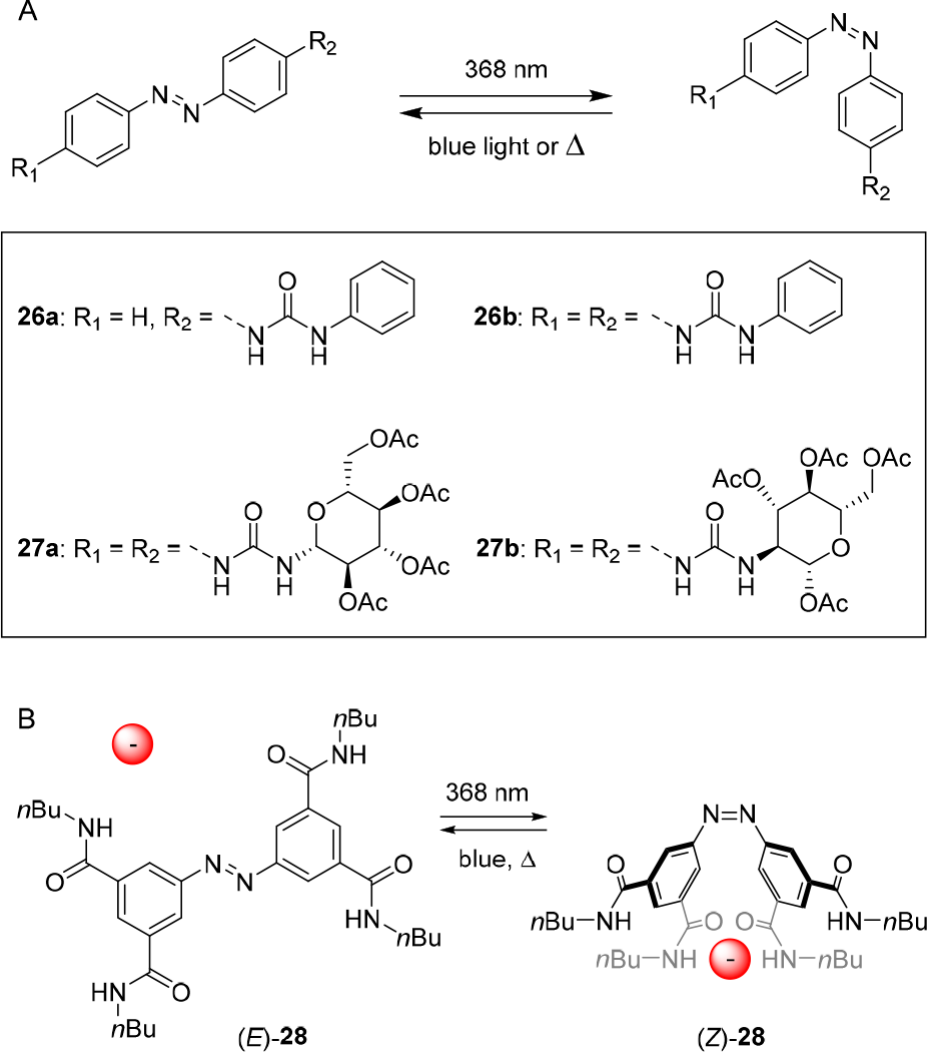
Light-controlled binding to azobenzene-based bis-urea (A) and tetra-amide (B) receptors.

The group of Jurczak prepared the mono- and bis-urea functionalized azobenzenes **26a** and **26b** shown in Figure 13A.^121^ Starting with the *E*-isomer, the *Z*-isomer could be generated by irradiation with 368 nm light, whereas the reverse *Z* → *E* isomerization process occurred spontaneously in the dark (thermally activated) or by exposure to 410 nm light. Binding of halides, carboxylates, HSO_4_^–^, and H_2_PO_4_^–^ led to a bathochromic shift of the π–π* absorption band in the UV–vis spectrum and, interestingly, shortened the half-life of the photogenerated *Z*-isomers. Moreover, the half-life decreased with increasing anion basicity and, thus, most likely relates to an increase in electron density and nitrogen (azobenzene) lone pair repulsion. ^1^H NMR titrations in DMSO/0.5%H_2_O using the benzoate anion revealed similar binding affinities for the monourea isomers (*E*)-**26a** and (*Z*)-**26a**, while the binding constant determined for bis-urea (*E*)-**26b** (*K*_11_ = 990 M^–1^) was about 4 times larger than its respective *Z*-isomer (*K*_11_ = 230 M^–1^). This observation is opposite to what is described for the stiff-stilbene based bis(thio)ureas above, in which anion binding to the *Z*-isomers was stronger than to the *E*-isomers. In this particular case, the authors suggested that the phenylurea rings of the receptor and the benzoate anion repel each other, leading to a lowering of the association constant.

In a later stage, the phenyl substituents were replaced for chiral β-d-glucopyranose rings to obtain receptors **27a** and **27b** (Figure 13A), which were designed to discriminate between chiral carboxylate guests.^122^ Both compounds showed almost identical binding affinity for a range of anions. Initial binding studies with CH_3_CO_2_^–^ and benzoate showed no significant difference in affinity constant between the *E*-isomers and the *Z*-isomers (around 10^3^ M^–1^ in DMSO/0.5%H_2_O). For benzoate, similar to the previous phenylurea substituted derivative, binding to the *Z*-isomers was up to three times weaker than to the *E*-isomers, which also here was attributed to unfavorable steric interactions with the (glucopyranose) substituents. When chiral BOC-protected phenylalanine and tryptophan were used, moderate selectivity for the d-enantiomers was observed for both, and again, binding affinities were up to three times smaller for the *Z*-isomers. Like in the previous example, enhancement of the rate of thermal *Z* → *E* isomerization upon substrate binding was observed in this study, which was more pronounced for the stronger binding d-enantiomers than for their weaker binding l-antipodes.

Expanding on this work, Jurczak and co-workers developed receptor **28**, bearing four hydrogen bond donating amide moieties (Figure 13B).^123^ In this design, anion binding to the *Z*-isomer occurred via four concurrent hydrogen-bonding interactions. As a result, binding of Cl^–^, CH_3_CO_2_^–^, benzoate, H_2_PO_4_^–^, and H_2_AsO_4_^–^ to (*Z*)-receptor **28** was up to three times stronger than binding to (*E*)-receptor **28**, as apparent from ^1^H NMR titration studies in DMSO-*d*_6_/0.5%H_2_O. Irradiation of the *E*-isomer with 368 nm light gave the *Z*-isomer in 22% yield. Interestingly, the rate of thermally activated *Z* → *E* isomerization slightly decreased in the presence of CH_3_CO_2_^–^ and H_2_PO_4_^–^ as compared to the receptor alone, whereas for the other azobenzene-based receptors described above, this rate was enhanced by the binding of anions. The authors reasoned that bridging of the amide substituents on either side of the molecule by the anion stabilizes the *Z*-isomer, leading to an increase in activation energy.

Similarly, the groups of Jeong and Langton developed azobenzene-based anion receptors with (thio)urea and squaramide groups in the benzylic position (compounds **71a**–**g** and **72a**–**i**).^124−126^ In addition to a detailed study of their switching and binding properties, these compounds were tested in transmembrane transport assays and are therefore discussed in section 3.3.

**Figure 14:**
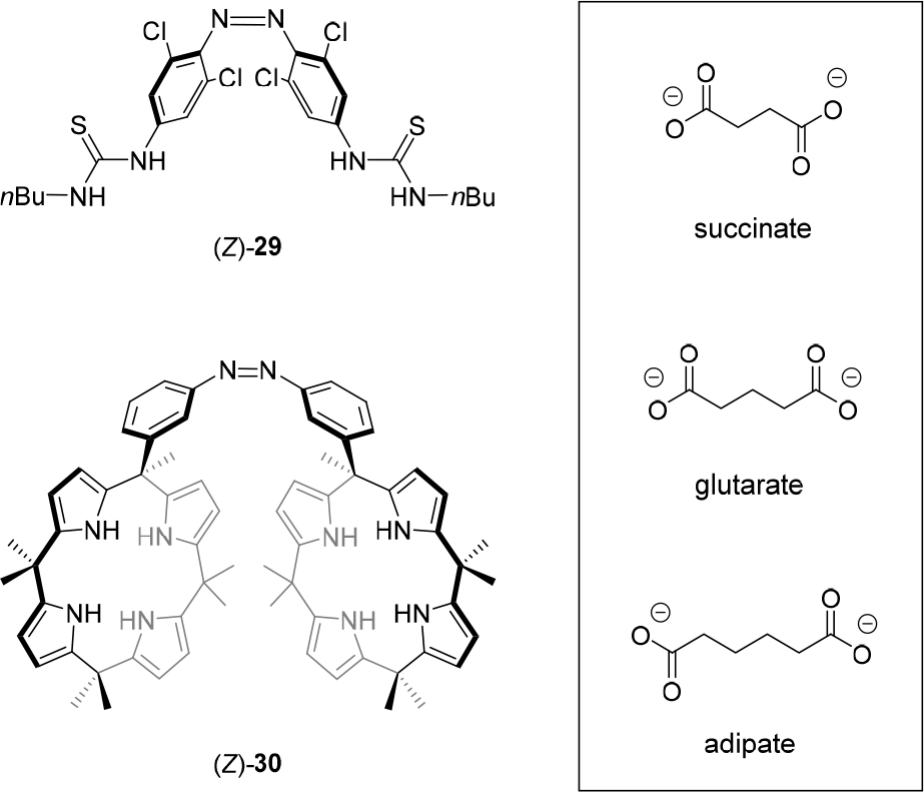
Azobenzene-based receptors that bind bis-carboxylate guests.

In a related study, the group of Wang prepared bis-thiourea receptor **29** based on *ortho*-chloro-substituted azobenzene, which can be switched by visible light (Figure 14).^127^ The use of red light to induce photoisomerization afforded a PSS (*Z*/*E*) ratio of 31:69. The binding of dicarboxylate anions with different spacer lengths was studied in DMSO using UV–vis spectroscopy. Overall, they bound slightly stronger to (*Z*)-**29** than to (*E*)-**29**, presumably because of their ability to bridge both thiourea moieties only in the former isomer. The difference in binding strength between isomers was most pronounced for the adipate dianion, for which the average binding constant of the PSS mixture (*K*_a_ = 3062 M^–1^) was two times higher than that of *E*-**29** alone (*K*_a_ = 1517 M^–1^).

In addition to bis-urea compounds, calix[4]pyrroles^128−131^ are widely used anion receptors, and they were therefore selected by the group of Kohnke to functionalize azobenzene with.^132^ The bis-calix[4]pyrrole **30** was, similar to the study by Wang and co-workers, used to modulate dicarboxylate binding (Figure 14). In this case, by ^1^H NMR titrations in DMSO-*d*_6_, a roughly 80-fold higher affinity constant, was found for succinate binding to the *Z*-isomer as compared to the *E*-isomer (*K*_a_ = 5.3 × 10^5^ M^–1^ and 6.4 × 10^3^ M^–1^, respectively). Adipate, in contrast, bound nearly 4 times weaker to *Z*-**30** than to *E*-**30** (*K*_a_ = 2.2 × 10^4^ M^–1^ and 8.6 × 10^4^ M^–1^, respectively). Apart from binding affinity, selectivity for dicarboxylate guests with different spacer length could thus be altered by photoswitching. That is, where the guests with the longer alkyl spacer bound the strongest to the *E*-isomer (adipate > glutarate > succinate) because of the larger distance between calix[4]pyrrole substituents, exactly the opposite trend was observed for the *Z*-isomer having these substituents closer together. Interestingly, in the presence of the guests, the rate of photochemical *E* → *Z* isomerization decreased with increasing association constant, which the authors state is in line with a concomitant increase in activation energy for N=N isomerization upon binding. Additionally, an influence on the thermal *Z* → *E* back isomerization was noted, albeit without apparent correlation between rate and the association constant.

**Figure 15:**
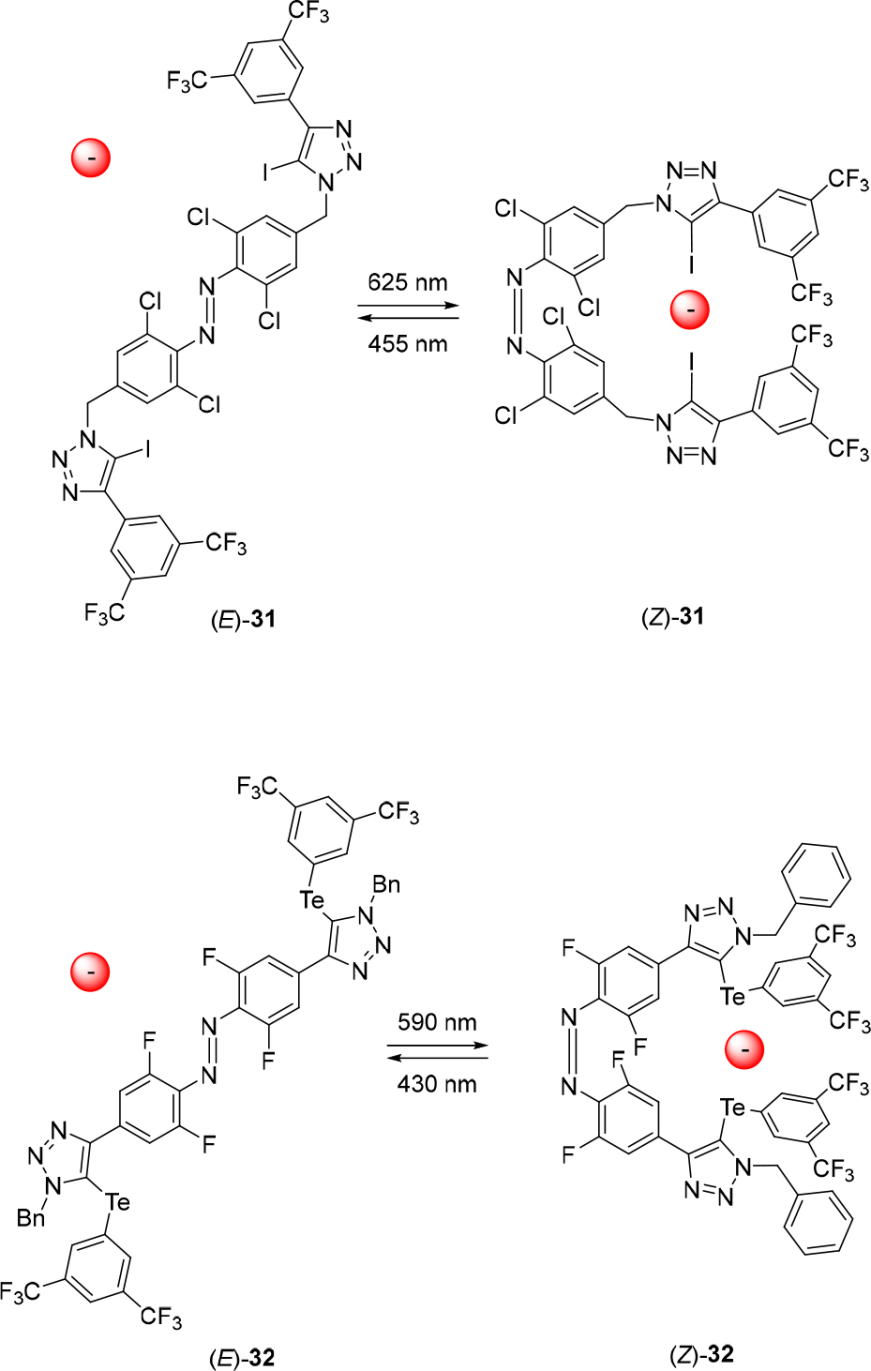
Photoswitchable halogen bonding and chalcogen bonding receptors.

The group of Langton functionalized *ortho*-chlorinated azobenzene with two iodotriazole moieties to develop a halogen-bonding anion receptor that could be switched with visible light (Figure 15).^133^ Irradiation of (*E*)-**31** with 625 nm light in acetone resulted in an (*E*/*Z*)-ratio of 17:83 at the PSS, as was determined using ^1^H NMR and UV–vis spectroscopy. The *E*-isomer could be partially regenerated upon subsequent irradiation with 455 nm light to give a PSS-ratio of 87:13 (*E*/*Z*) or by heating to 60 °C for 1 h to obtain almost quantitative amounts of (*E*)-**31**. ^1^H NMR binding studies in acetone-*d*_6_ revealed that Cl^–^, Br^–^, and I^–^ were bound strongly by (*Z*)-**31** via cooperative 1:1 binding, and weakly to (*E*)-**31** via noncooperative 1:1 and 1:2 complexation. The largest affinity difference was found for the binding of Cl^–^, which was 53-fold higher for the *Z*-isomer (*K*_a_ = 3.2 × 10^4^ M^–1^) than for the *E*-isomer (*K*_a(1:1)_ = 5.9 × 10^2^ M^–1^). Upon chloride binding, the authors noted that the *Z* → *E* thermal half-life increased 3-fold, while no changes were observed when noncoordinative PF_6_^–^ was added instead. In the same work, a methylated triazolium version of **31** was used as a catalyst for a Friedel–Crafts alkylation, Mukaiyama aldol reaction and Michael addition, where the *Z*-isomer was a more potent catalyst than the *E*-isomer for the former two of the three reaction types.

Simultaneously, Han, Zhang, and co-workers developed a tellurium-based chalcogen-bonding receptor based on visible-light-responsive *ortho*-fluorinated azobenzene (Figure 15).^134^ Photoswitching was studied by ^1^H NMR and UV–vis spectroscopy in acetone, which showed that irradiation of (*E*)-**32** with 590 nm light gave an (*E*/*Z*)-ratio of 8:92 at the PSS. The formed isomer (*Z*)-**32** exhibited high thermal stability with a half-life of 48.1 days. Subsequent irradiation of the PSS_590_-mixture with 430 nm light resulted in reformation of (*E*)-**32**, with an (*E*/*Z*)-ratio of 85:15. A ^1^H NMR titration experiment indicated that both the *E*- and the *Z*-isomer bound Cl^–^ and Br^–^ via a combination of 1:1 and 1:2 binding modes. However, binding in (*Z*)-**32** occurred with significant cooperativity between the two tellurium binding residues, while (*E*)-**32** did not show any signs of cooperative binding. Binding constants were obtained for Cl^–^ and Br^–^ in acetone, which were both higher for (*Z*)-**32** (*K*_a(1:1)_ = 3769 M^–1^ and 1249 M^–1^, respectively) than for (*E*)-**32** (*K*_a(1:1)_ = 807 M^–1^ and 324 M^–1^, respectively). Importantly, reversible photoswitching remained possible in the presence of the anions. Furthermore, methylation on the triazole position remarkably enhanced the binding affinity compared to that of the original receptor, while its photoresponsive behavior was similar. This triazolium analogue was used as a halide abstraction agent to catalyze a Friedel–Crafts alkylation reaction between benzhydryl chloride and 1,3,5-trimethoxybenzene. Here, conversion toward the alkylated product was low in the presence of (*E*)-**32**, but upon irradiation with 590 nm light and generation of (*Z*)-**32**, the conversion was strongly enhanced, which is in line with their respective chloride binding affinity and concomitant halogen abstraction ability.

**Figure 16:**
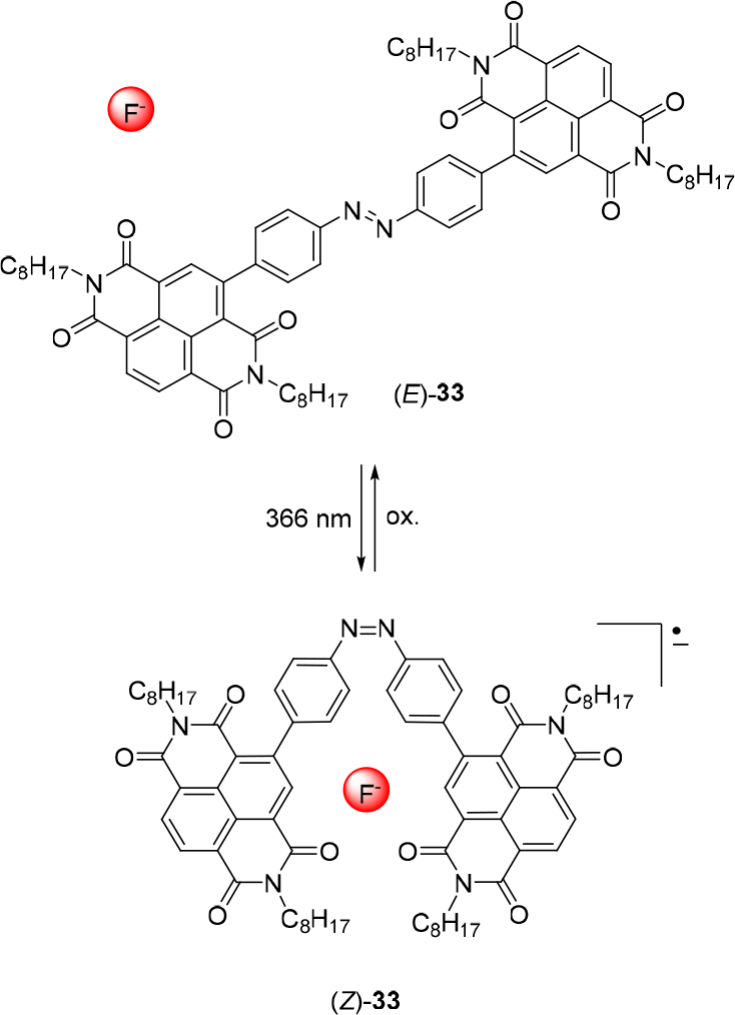
Light-controlled fluoride binding to a bis-NDI receptor.

While the photogenerated *Z*-isomer of azobenzene usually converts thermally to the thermodynamically more stable *E*-form, Bandyopadhyay, Bhosale, and co-workers developed naphthalenediimide (NDI) appended receptor **33**, of which the *Z*-isomer was stabilized by fluoride binding via anion−π interactions (Figure 16).^135^ Starting with (*E*)-**33**, the respective *Z*-isomer was produced by irradiation with 366 nm light, and the reverse Z → *E* isomerization could be induced using 500 nm light. However, irradiation of (*E*)-**33** in the presence of F^–^ led to quantitative and irreversible formation of the *Z*-isomer. Spectroscopic characterization of the 1:1 sandwich-like (*Z*)-**33** ⊂ F^–^ complex revealed a radical anionic NDI species,^136^ while 1:2 (*Z*)-**33** to F^–^ complexation upon addition of excess fluoride gave rise to a dianionic NDI form. Both complexes are the result of electron transfer between the fluoride anions and the *Z*-receptor, which did not occur when other anions were used instead. Thermal *Z* → *E* isomerization in the presence of fluoride was only observed upon oxidation of the stable radical complexes, which causes dissociation of the anion, further indicating that binding stabilizes the *Z*-isomer.

Where in some examples, an effect of anion binding on the photoisomerization process was observed, as was also described for stiff-stilbene (*vide supra*),^99,110,114^ most studies only report an influence on the rate of thermal isomerization and half-life. This influence is either ascribed to electronic effects or to stabilization of the photogenerated *Z*-isomer by a substrate that is able to bridge the binding motifs. Further research will be necessary to dissect these contributions and to improve our understanding of how substrate binding affects isomerization behavior.^90^

#### 2.3.3 Encapsulation by Foldamers

**Figure 17:**
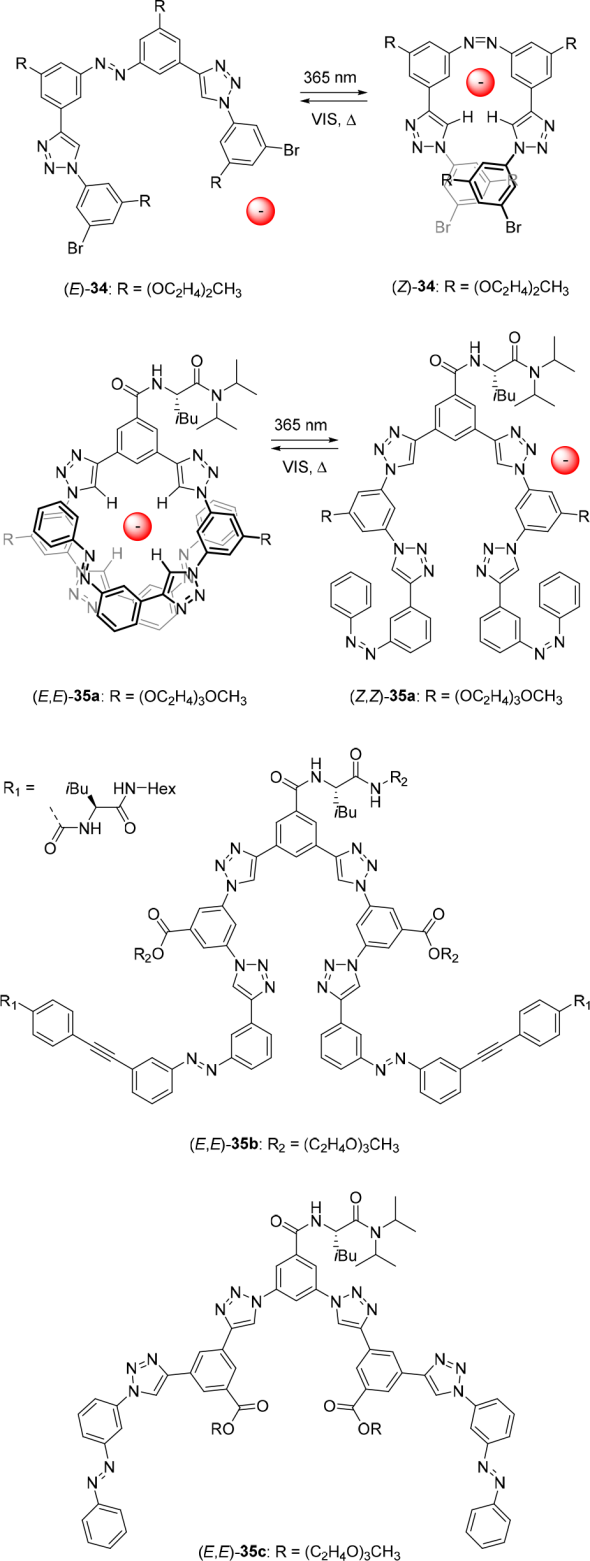
Photoswitchable foldamers and examples of their anion encapsulation.

Besides molecular tweezers, foldamers^137^ have been developed into photoswitchable anion receptors. So far, these have all been based on azobenzene. For example, the group of Jiang functionalized azobenzene with two phenyl-1,2,3-triazole motifs to obtain the halide-binding foldamer **34** (Figure 17).^138^ Exposure to 365 nm light afforded a PSS (*E*/*Z*) ratio of 18:82, while the use of visible light gave a ratio of 77:23. In addition, full thermal conversion from the photogenerated (*Z*)-**34** to (*E*)-**34** was shown to occur in the dark at room temperature. In acetone solution, the *Z*-isomer bound Cl^–^ (*K*_a_ = 290 M^–1^) and Br^–^ (*K*_a_ = 87 M^–1^) anions approximately four times stronger than the *E*-isomer (*K*_a_ = 70 M^–1^ for Cl^–^ and 22 M^–1^ for Br^–^). Slightly smaller differences (2–3-fold) were found for I^–^, NO_3_^–^ and HSO_4_^–^, which the authors attributed to the larger size of these anions. In similarity to the molecular tweezer approach, a possible explanation for the difference in affinity is that in the *Z*-isomer the hydrogen bond donors, i.e., the polarized C–H protons of both triazole moieties, participate in hydrogen bonding to a single anion, while in the *E*-isomer, only one of these units is able to take part in this.

The group of Flood developed triazole-based foldamer **35a** containing two peripheral azobenzene photoswitches (Figure 17).^139^ This foldamer is able to adopt a helical conformation in its *E*,*E*-form, which is stabilized by intramolecular π–π stacking interactions. However, irradiation with 365 nm light in acetonitrile, giving a mixture of (*Z*,*E*)-**35a** and (*Z*,*Z*)-**35a** in a in 2:1 ratio, led to helix destabilization. Subsequent irradiation at 436 nm afforded a mixture containing the (*E*,*E*)-**35a**, (*Z*,*E*)-**35a**, and (*Z*,*Z*)-**35a** in a 67:30:3 ratio. UV–vis titration experiments performed in acetonitrile revealed a Cl^–^ binding affinity for the *E*,*E*-isomer of *K*_a_ = 3.0 × 10^3^ M^–1^ and an average constant for the PSS_365_ mixture of *K*_a_ = 3.8 × 10^2^ M^–1^. Furthermore, from ^1^H NMR titrations using mixtures of (*E*,*E*)-**35a**, (*Z*,*E*)-**35a**, and (*Z*,*Z*)-**35a** in dichloromethane-*d*_2_, it was concluded that the association constant of the *Z*,*E*-isomer lies in between that of the other two isomers, in line with the authors’ hypothesis that a positive correlation exists between binding strength and the degree of preorganization of the binding site of the foldamer. Importantly, by measuring the conductivity of a foldamer/Cl^–^ solution in acetonitrile, it was demonstrated *in situ* that the unbound Cl^–^ concentration could be controlled by light.

Flood and co-workers further optimized this design with phenylacetylene substituents bearing hydrogen bond donating and accepting groups toward foldamer (*E*,*E*)-**35b** (Figure 17).^140^ These substituents improved the stability of the helical conformation of (*E*,*E*)-**35b** and, hence, led to stronger Cl^–^ binding. In this case, irradiation of (*E*,*E*)-**35b** with 365 nm light gave a PSS mixture containing the *E*,*E*-isomer, *Z*,*E*-isomer, and *Z*,*Z*-isomer in a 8:28:64 ratio. Binding studies with Cl^–^ by UV–vis spectroscopy in acetonitrile/tetrahydrofuran (1:1 v/v) revealed an affinity constant for (*E*,*E*)-**35b** of *K*_a_ = 9.70 × 10^5^ M^–1^, whereas the average constant for the PSS_365_ mixture was again lower and determined to be 1.16 × 10^4^ M^–1^. An 84-fold affinity difference was thus achieved, which is the highest reported to date with a foldamer design.

More recently, the same group described structurally similar foldamer (*E*,*E*)-**35c** (Figure 17), which could form single and double helical structures depending on the size of the anionic guest.^141^ In short, addition of anions with a volume smaller than 45 Å^3^ (i.e., Cl^–^, Br^–^, I^–^, NO_2_^–^, and NO_3_^–^) to the *E*,*E*-isomer led to a single helical 1:1 complex, which like the unbound foldamer was stabilized by intramolecular π–π stacking interactions. Irradiation of this *E*,*E*-isomer with 365 nm light in acetonitrile gave a PSS mixture of (*E*,*E*)-**35c**, (*Z*,*E*)-**35c**, and (*Z*,*Z*)-**35c** in a 16:29:55 ratio. In line with previous studies, the PSS_365_ mixture exhibited an overall anion binding affinity lower than that of the *E*,*E*-isomer alone, with the largest 8-fold difference found for Br^–^ (*K*_a(*E*,*E*)_ = 1.6 × 10^4^ M^–1^ and *K*_a(PSS)_ = 2.0 × 10^3^ M^–1^). Remarkably, addition of anions with a volume larger than 45 Å^3^ (i.e., SCN^–^, BF_4_^–^, ClO_4_^–^, ReO_4_^–^, PF_6_^–^, and SbF_6_^–^) to the foldamer preferentially gave a double helix containing this isomer and the anion in a 2:1 ratio. Energy minimizations by DFT of possible 1:1 complexes showed that accommodation of these larger anions would require the foldamer to adopt a more extended conformation, which would reduce intramolecular π–π stacking interactions. Furthermore, it would result in unfavorable torsional angles, thereby decreasing helix stability. DFT optimization of the 2:1 complex illustrated that double helix formation is indeed energetically favored in this case. Although for all anions larger than 45 Å^3^ a mixture of 1:1 and 2:1 complexes was observed in solution, the stability of the double helices increased with anion size across series with the same geometry. Interestingly, UV–vis studies showed that when the larger anions were added to the PSS_365_ mixture, 1:1 complexation was preferred. Apart from controlling anion binding affinity, as shown in previous reports, here with the larger anions binding stoichiometry as well as secondary structure could thus be altered by light using the larger anions.

#### 2.3.4 Macrocycle Contraction

Another approach toward photoswitchable anion binding is based on incorporating molecular photoswitches into macrocyclic receptors. Where cyclization itself may improve binding site preorganization and therefore enhance affinity, photoinduced contraction of the macrocyle will usually make the binding site less accessible. Potentially, macrocycle contraction/expansion can lead to large differences in binding affinity between photoaddressable states, however, this approach may go at the cost of switching efficiency due to strain that is built up.^142,143^

**Figure 18:**
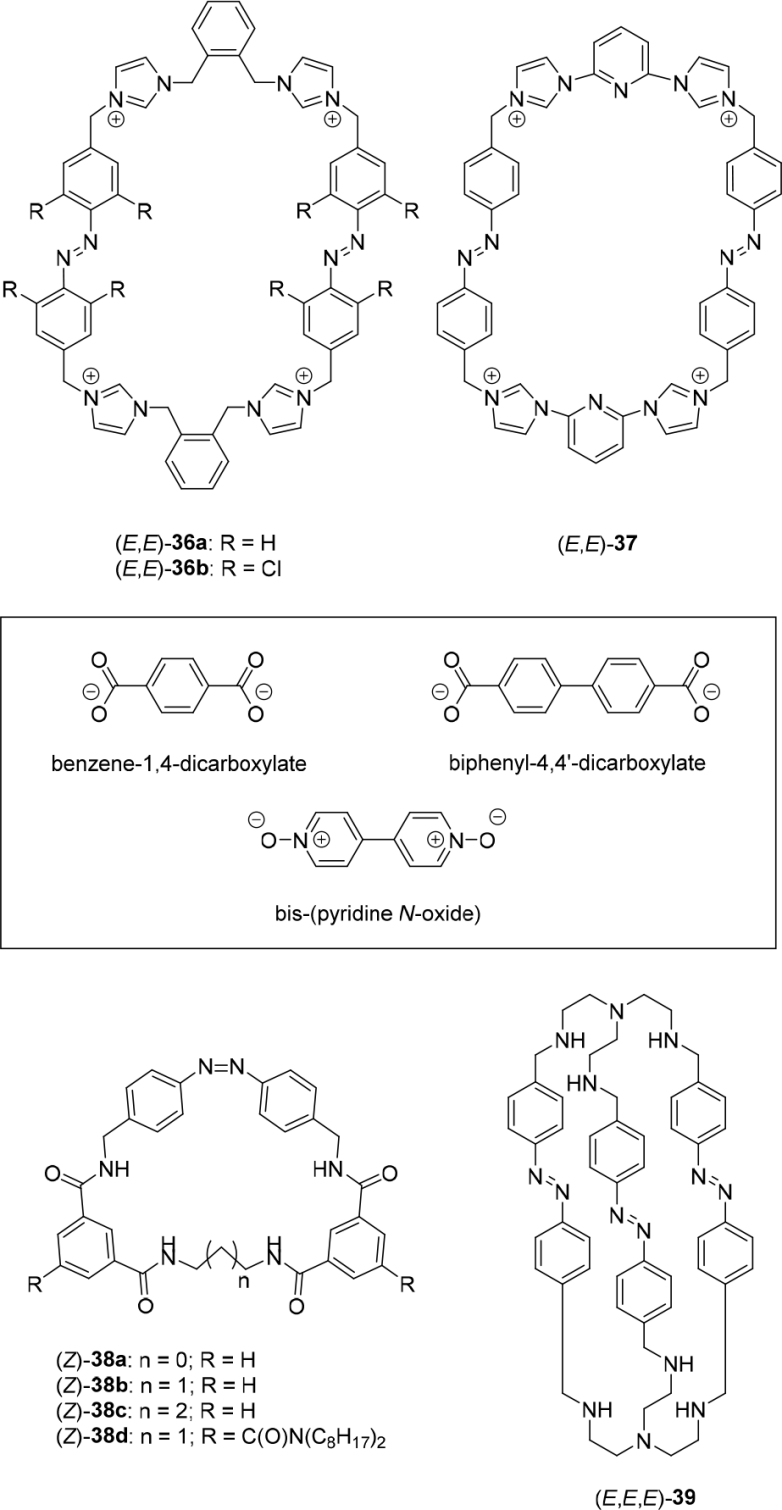
Azobenzene-containing macrocycles that can be expanded and contracted by light.

In 2016, Scherman and co-workers developed macrocyclic receptor **36a** containing two azobenzene units and four hydrogen-bond-donating imidazolium groups (Figure 18).^144^ In the expanded *E*,*E*-isomer, the macrocycle was shown capable of encapsulating bis(pyridine *N*-oxide) and biphenyl-4,4′-dicarboxylate guests in a 1:1 stoichiometry. The binding constant of the bis-*N*-oxide guest was determined by ^1^H NMR titrations in acetonitrile-*d*_3_ to be in the order of *K*_a_ ∼ 10^3^ M^–1^. These titrations, as well as DFT calculations, supported that binding occurs in a 1:1 fashion through four simultaneous hydrogen-bonding interactions between both oxygen atoms of the guest and the imidazolium protons of the macrocycle. Excitation of the *E*,*E*-isomer with 350 nm light led to a PSS mixture containing (*E*,*E*)-**36a**, (*Z*,*E*)-**36a**, and (*Z*,*Z*)-**36a** in a 18:38:44 ratio. Subsequent irradiation with 420 nm light led to a ratio of 64:28:9, and furthermore, the initial *E*,*E*-isomer could be recovered in quantitative yield via thermal back isomerization in the dark. No significant guest binding to the photogenerated *E*,*Z*- and *Z*,*Z*-isomers was observed, most likely because of the reduced binding cavity size. By irradiation of a host/guest mixture with 350 nm followed by 420 nm light, release and uptake of the guest was successfully demonstrated *in situ* using ^1^H NMR spectroscopy.

The group of Sessler developed structurally related macrocycle **37** with two bridging pyridyl groups (Figure 18).^145^ Here, 365 nm irradiation of the *E*,*E*-isomer gave a PSS mixture containing (*E*,*E*)-**37**, (*Z*,*E*)-**37**, and (*Z*,*Z*)-**37** in a 15:41:44 ratio. The former isomer strongly bound biphenyl-4,4′-dicarboxylate (*K*_a_ = 1.63 × 10^6^ M^–1^) in DMSO-*d*_6_, as was determined by ^1^H NMR titrations, while the *Z*,*E*- and *Z*,*Z*-isomers did not display significant binding. On the contrary, benzene-1,4-dicarboxylate could bind to all isomers of the receptor owing to its smaller size. In a clever experiment, addition of a mixture of biphenyl-4,4′-dicarboxylate and benzene-1,4-dicarboxylate to (*E*,*E*)-**37** showed preferential encapsulation of the larger biphenyl guest. Upon irradiation with 365 nm light to reach the PSS_365_ mixture, this larger guest was released and binding of the smaller benzene-1,4-dicarboxylate was favored. Hence, this study represents a means to control binding selectivity by light, somewhat similar to the work by Kohnke (see section 2.3.2). In addition, it was demonstrated that the biphenyl-4,4′-dicarboxylate guest could be released from (*E*,*E*)-**37** by addition of an excess of Cl^–^ as a competitive guest or, alternatively, by protonation using trifluoroacetic acid, which could be reversed by adding triethylamine.

Liu, Zhang, and co-workers introduced tetra-*ortho*-chloro substituents to the azobenzene motifs in the macrocycle developed by the group of Scherman, enabling photoisomerization using red light.^146^ Excitation of the *E*,*E*-isomer could now be performed with 650 nm light and gave (*E*,*E*)-**36b**, (*E*,*Z*)-**36b**, and (*Z*,*Z*)-**36b** in a 4:36:60 ratio. The reverse process could be triggered by subsequent irradiation with 410 nm light, resulting in a mixture containing (*E*,*E*)-**36b** and (*E*,*Z*)-**36b** in a 87:13 ratio. Competitive binding experiments using biphenyl-4,4′-dicarboxylate and benzene-1,4-dicarboxylate as guests, before and after irradiation, had a similar outcome as described by the group of Sessler. Furthermore, the authors quantified the binding affinity of benzene-1,4-dicarboxylate to (*E*,*E*)-**36b** and (*Z*,*Z*)-**36b** in DMSO-*d*_6_ by ^1^H NMR titrations, giving *K*_a_ = 455 M^–1^ and *K*_a_ = 6527 M^–1^, respectively. For the larger biphenyl-4,4′-dicarboxylate guest, only considerable binding to (*E*,*E*)-**36b** was observed (*K*_a_ = 466 M^–1^). These results indicate that the contracted macrocycle is capable of forming four concerted hydrogen bonds with the smaller guest, whereas the expanded macrocycle is not, which was corroborated by DFT calculations.

Recently, Xiong and He reported monoazobenzene based macrocycles **38a-d**, containing four amide hydrogen bond donors (Figure 18) to which SO_4_^2–^ binding could be controlled.^147^ The photoisomerization behavior was studied using (*E*)-**38a**, and when this compound was irradiated with ∼365 nm light, (*Z*)-**38a** formed in quantitative yield. The reverse process was studied in the presence of SO_4_^2–^ and could be induced with ∼420 nm light, resulting in a 62:38 (*E*/*Z*) PSS mixture. For the *Z*-isomers, a high binding affinity and selectivity for SO_4_^2–^ was determined by ^1^H NMR titrations in DMSO-*d*_6_, with the largest *K*_a_ values of 2.3 × 10^4^ M^–1^ found for (*Z*)-**38b**. For (*E*)-**38b**, a 32-fold lower affinity constant of *K*_a_ = 7.1 × 10^2^ M^–1^ was observed. DFT geometry optimization of (*Z*)-**38a**–**c** corroborated that the binding site in this isomer is preorganized and has a high complementarity for SO_4_^2–^, allowing the formation of multiple receptor–anion hydrogen bonds. On the other hand, the solid-state structures of (*E*)-**38a** and **38b** showed adoption of a bow-shaped conformation, in which simultaneous amide hydrogen bonding to the dianion is less likely. Additionally, analogue (Z)-**38d** appended with solubilizing aliphatic chains was used in a solid–liquid (i.e., chloroform-*d*) extraction of (Me_4_N)_2_SO_4_, where photoirradiation toward the *E*-isomer successfully resulted in release of the guest.

In a different approach, the group of Kataev prepared bicyclic macrocycle **39** consisting of two tris(2-aminoethyl)amine (tren) moieties that were connected by three azobenzenes (Figure 18).^148^ Irradiation of (*E*,*E*,*E*)-**39** with 365 nm light in acetate buffer at pH 3.6 gave rise to a mixture of (*E*,*E*,*E*)-**39**, (*E*,*E*,*Z*)**-39**, (*E*,*Z*,*Z*)**-39**, and (*Z*,*Z*,*Z*)**-39** in a 45:35:13:7 ratio, while in chloroform-*d*/methanol-*d*_4_, the latter (*Z*,*Z*,*Z*)**-39** was the main isomer formed upon irradiation (60%). The photogenerated isomers underwent thermally activated isomerization back to (*E*,*E*,*E*)-**39**, which could be accelerated by irradiation with visible light. Binding studies performed using ^1^H NMR spectroscopy in acetate buffer (in D_2_O at pH 3.6 at which the tren moieties are protonated), showed slight differences in the binding of the sodium salts of F^–^ and ClO_4_^–^ to each isomer. That is, the former anion was bound most strongly by (*E*,*E*,*E*)-**39** (*K*_a_ = 5.0 × 10^3^ M^–1^), slightly weaker by (*E*,*E*,*Z*)-**39** (*K*_a_ = 2.0 × 10^3^ M^–1^), and the weakest by (*E*,*Z*,*Z*)-**39** (*K*_a_ = 1.4 × 10^3^ M^–1^). For the latter ClO_4_^–^, this trend was opposite, with the binding being the strongest by (*E*,*Z*,*Z*)-**39** (*K*_a_ = 8.3 × 10^2^ M^–1^), weaker by (*E*,*E*,*Z*)-**39** (*K*_a_ = 1.7 × 10^2^ M^–1^), and the weakest by (*E*,*E*,*E*)-**39** (*K*_a_ = 5.9 × 10^1^ M^–1^). Furthermore, these anions stabilized the *Z*-containing isomers under irradiation and remarkably, isomerization could be directed toward the *E*,*E*,*Z*-isomer upon encapsulation of the macrocycle by cucurbit[8]uril, while F^–^ addition led to release of the macrocycle as the *E*,*E*,*E*-isomer.

**Figure 19:**
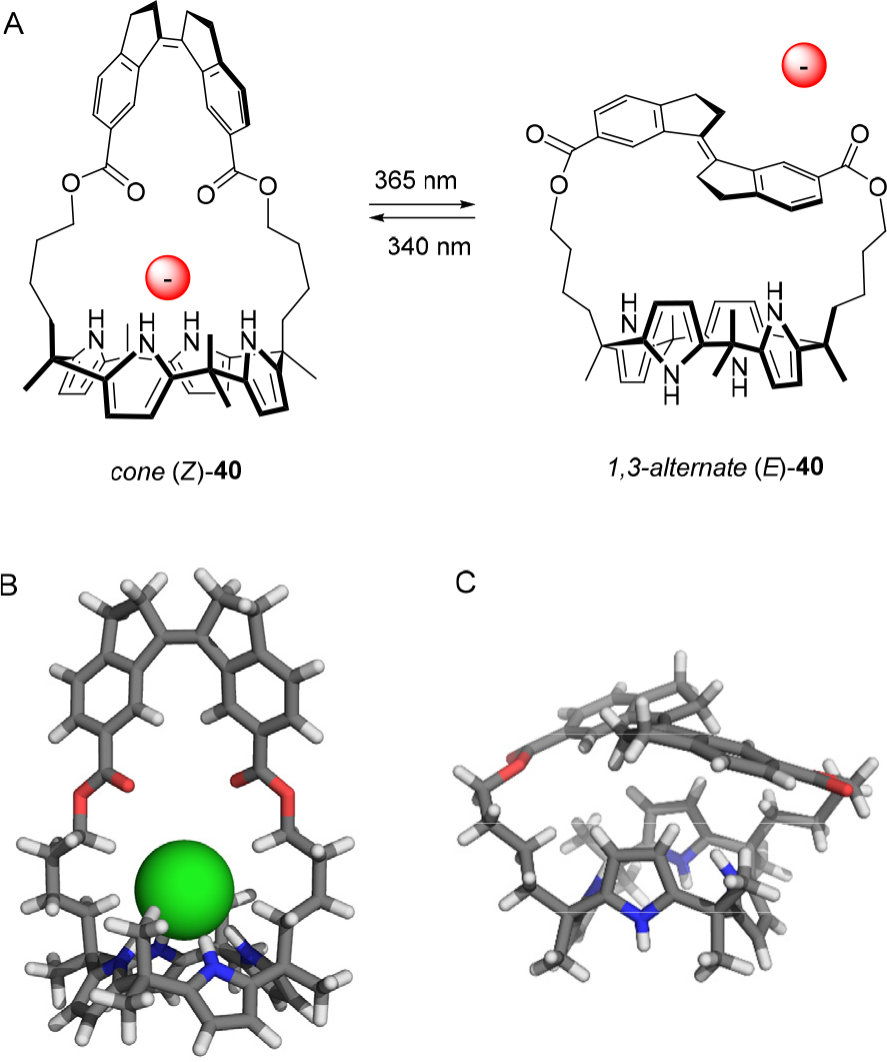
Control of binding affinity to strapped calix[4]pyrrole receptor (A), DFT-calculated structure of bound (*Z*)-**40** (B), and single-crystal X-ray structure of unbound (*E*)-**40** (C).

Recently, our group developed bicyclic receptor **40** (Figure 19A), which is based on calix[4]pyrrole containing a stiff-stilbene photoswitch as bridging unit.^149^ Irradiation of (*Z*)-**40** with 365 nm light gave (*E*)-**40** in virtually quantitative yield, whereas subsequent irradiation with 340 nm light afforded a PSS mixture with a 73:27 (*E*/*Z*) ratio. In DMSO-*d*_6_, ^1^H NMR titration with Cl^–^ revealed strong binding to (*Z*)-**40**, while addition to (*E*)-**40** did not give any noticeable spectral changes. Further binding studies by isothermal titration calorimetry (ITC) in acetonitrile revealed an association constant for the *Z*-isomer of *K*_a_ = 1.6 × 10^6^ M^–1^, similar to what has been reported for structurally related strapped calix[4]pyrroles.^130,150−152^ With the *E*-isomer, on the other hand, no significant heat release was observed in the ITC titrations. Nevertheless, the binding constant was determined by ^1^H NMR titrations in acetonitrile-*d*_3_ as *K*_a_ = 2.0 × 10^2^ M^–1^. Thus, an 8000-fold difference in binding affinity was found between the two photoaddressable states and, to our best knowledge, this is the highest difference achieved for photoswitchable anion receptors so far.

DFT optimization of the (*Z*)-**40** ⊂ Cl^–^ complex indicated that the cavity provided by the strap is of suitable size for encapsulation of this anion, which is held in place by four hydrogen bonds to the N–H atoms of calix[4]pyrrole in the *cone* conformation (see Figure 19B). The structure of the *E*-isomer was elucidated by single-crystal X-ray analysis, revealing a substantially compressed cavity and a *1,3-alternate* calix[4]pyrrole conformation (see Figure 19C). This conformation is known to be energetically favored^153^ but converts to the cone conformation upon anion binding. Attempts to optimize the geometry of a possible (inverted) cone geometry for the (*E*)-**40** ⊂ Cl^–^ complex by DFT calculations were unsuccessful and hinted at a very high energy cost for adopting the cone conformation because of the ring-strain generated in the *E*-isomer.

**Figure 20:**
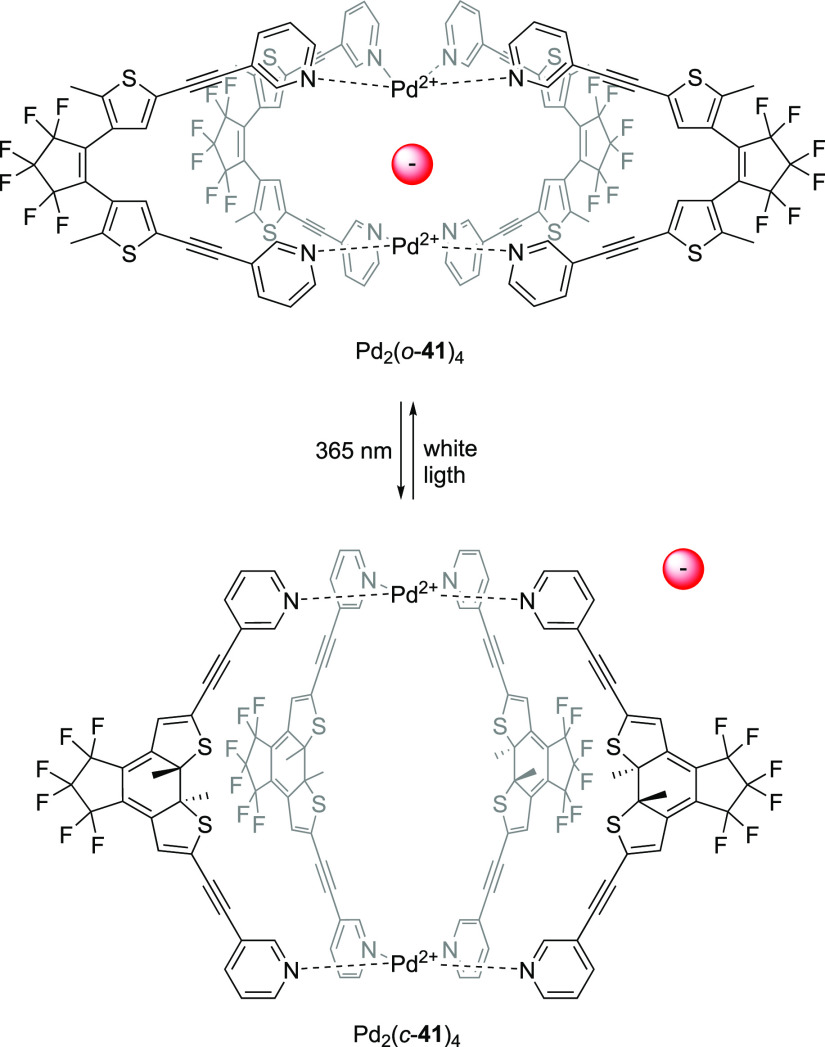
Photoswitchable anion binding to a metal–organic cage.

The group of Clever developed metal–organic cage Pd_2_(**41**)_4_, consisting of four photoresponsive bis-monodentate pyridyl-substituted dithienylethene ligands, able to coordinate to the Pd^2+^ metal centers (Figure 20).^154^ With the dithienylethene ligands in their *open* form, the cage structure was somewhat compressed. However, ring-closing by 365 nm irradiation rigidified the ligands, causing the cage to adopt a more expanded geometry. This expansion could be reverted by irradiation with white light to regenerate the *open*-form cage. Remarkably, the association constant for encapsulation of spherical dianionic [B_12_F_12_]^2–^ guest, determined by ^1^H NMR titrations in acetonitrile-*d*_3_, was significantly larger (*K*_a_ = 3.2 × 10^4^ M^–1^) for the compressed *open*-form cage than for the extended *closed*-form cage (*K*_a_ = 6.7 × 10^2^ M^–1^). Presumably, the more flexible Pd_2_(*o*-**41**)_4_ is capable of adapting its structure to the guest and allows a smaller distance between the positively charged metal centers than rigidified Pd_2_(*c*-**41**)_4_. Reversible photoswitching was additionally demonstrated in the presence of [B_12_F_12_]^2–^, thereby allowing control over the unbound/bound guest concentration in solution.

**Figure 21:**
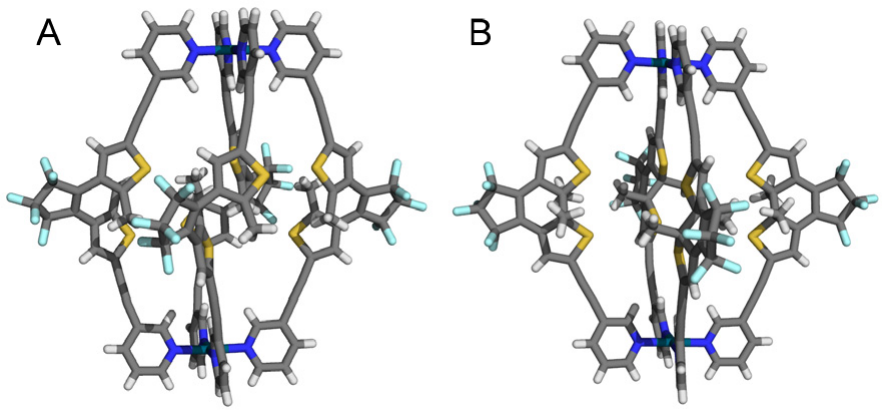
Single-crystal X-ray structures of (A) ring-open and (B) ring-closed Pd_2_(**41**)_4_ cages.

In a later stage, Clever and co-workers further examined this cage, as well as derivatives having differently substituted linkers.^155^ Additional binding studies using other anionic guests revealed that a light-induced affinity change could also be achieved with [1-H-*closo*-1-CB_11_F_11_]^−^, of which the association constants in acetonitrile-*d*_3_ were *K*_a_ = 258 M^–1^ for Pd_2_(*o*-**41**)_4_ and *K*_a_ = 6 M^–1^ for Pd_2_(*c*-**41**)_4_. Although other borate anions, i.e., [*closo*-B_10_Cl_10_]^2–^, [12-HC≡C-*closo*-1-CB_11_H_11_]^−^ and [Hg(*closo*-1-CB_11_F_11_)_2_]^−^, also bound to Pd_2_(*o*-**41**)_4_ with constants in the range of *K*_a_ ∼ 10^2^–10^3^ M^–1^, their addition to Pd_2_(*c*-**41**)_4_ led to precipitation. The results of the ^1^H NMR titration studies, including the earlier reported binding of [B_12_F_12_]^2–^ to cages Pd_2_(*o*-**41**)_4_ and Pd_2_(*c*-**41**)_4_, were corroborated by ITC. Interestingly, where in their previous work the authors reported the solid-state structure of the *open*-form cage, this time they managed to crystallize the *closed*-form cage (see Figure 21), as well as intermediate structure Pd_2_(*o*-**41**)_2_(*c*-**41**)_2_. Single crystals of the latter were obtained by starting from a solution of Pd_2_(*o*-**41**)_4_ and exposing it to sunlight. This intermediate cage structure indicated that photoisomerization from Pd_2_(*o*-**41**)_4_ to Pd_2_(*c*-**41**)_4_ is a stepwise process.

The *open*-form isomer of dithienylethene is able to adopt opposite helical conformations (*P* and *M*), which interconvert rapidly at room temperature. This means that the Pd_2_(*o*-**41**)_4_ cage will exist as a dynamic statistical mixture of six isomers with different ratios of *P* and *M* helical ligand conformers. Ring-closing by irradiation with UV-light gives *closed*-ligand enantiomers with either *R*,*R* or *S*,*S* configuration, which is governed by the helical chirality of the preceding *open*-form ligand. Hence, it potentially leads to six different configurational isomers of the *closed* cage having different (*R*,*R*)-**41** and (*S*,*S*)-**41** ligand ratios. In a recent study, the group of Clever investigated the photoswitching process using a structurally related cage in the presence of a chiral 1*R*-(−) camphor sulfonate guest.^156^ This encapsulation induced a preferred helical conformation in the *open*-form ligand as was demonstrated by CD spectroscopy. In addition, chiral HPLC analysis revealed that the obtained enantiomeric excess for the *closed*-form ligand was approximately 25%, leading to the suggestion that only one out of the four ligands in the Pd_2_(*o*-**41**)_4_ cage could undergo stereoselective ring-closing in the presence of chiral guest. This result led the authors to conclude that ring-closing of the first ligand already triggers guest release.

In 2021, Clever and co-workers developed a system responsive to multiple stimuli, where reversible photoswitching between open and closed dithienylethene ligands resulted in the assembly of either cage or bowl-shaped structures.^157^ Complexation of an additional ligand to the unsaturated bowl-shape structures led to the formation of heteroleptic cages, of which the encapsulation properties depended on the size of the added ligand. The binding behavior of the bowls and cages could be regulated by light or a chemical stimulus to control the binding and release of propane-1,3-bis-sulfonate.

#### 2.3.5 Anthracene Dimerization

**Figure 22:**
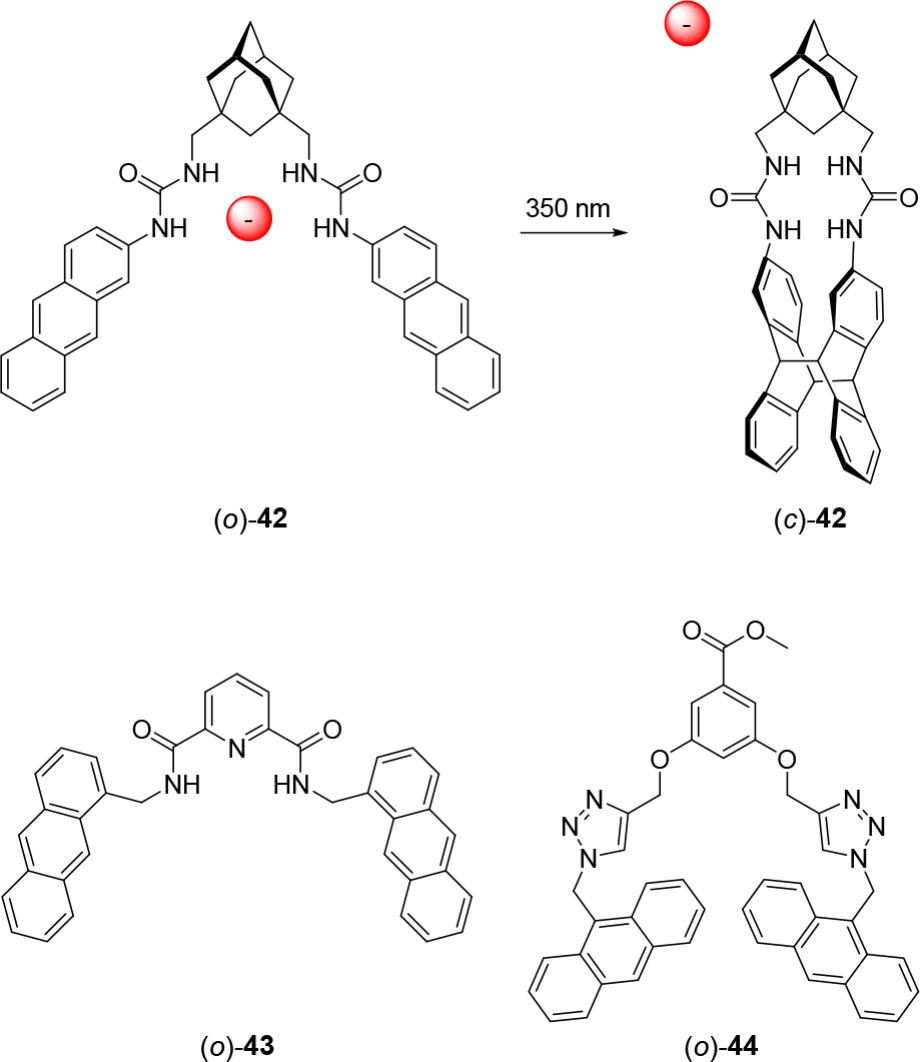
Photoinduced anthracene dimerization to control binding affinity.

Another method for controlling anion binding by light is based on macrocyclization through anthracene dimerization.^158−161^ In 2015, Mlinarić-Majerski and co-workers described adamantine-functionalized bis-urea receptor **42**, appended with two anthracene units (Figure 22).^162^ The nondimerized form (*o*)-**42** bound Cl^–^, H_2_PO_4_^–^, and CH_3_CO_2_^–^, whereas the addition of F^–^ led to deprotonation. While the binding of Cl^–^ in DMSO was relatively weak (*K*_a_ ∼ 10^1^ M^–1^), the more basic H_2_PO_4_^–^ and CH_3_CO_2_^–^ anions bound significantly stronger with *K*_a_ values in the order of 10^3^–10^4^ M^–1^. When (*o*)-**42** was irradiated with 350 nm light, an intramolecular [4 + 4] cycloaddition occurred between the two anthracene moieties, affording (*c*)-**42** in 30% yield. Generally, such cyclization products can thermally convert back to the starting anthracenes, however, in this case, it was found to be stable at 100 °C for over 4 h, impeding reversible operation. Interestingly, while H_2_PO_4_^–^ bound slightly weaker to (*c*)-**42** than to (*o*)-**42**, for CH_3_CO_2_^–^ this was the opposite [*K*_a_ > 10^6^ M^–1^ for (*c*)-**42**]. Photocyclization in the presence of these anions was hampered, which the authors ascribed to an increase in the distance between the anthracene units upon anion binding. This would be slightly surprising though, considering the high stability constant of the (*c*)-**42** ⊂ CH_3_CO_2_^–^ complex.

Likewise, Ulatowski and Melaniuk developed anthracene-appended pyridinedicarboxamide **43** (Figure 22).^163^ Irradiation of (*o*)-**43** with 365 nm light gave nearly quantitative formation of (*c*)-**43** and, although also here thermal back conversion did not occur, irradiation with 254 nm light was used to partially revert cycloaddition. ^1^H NMR titrations with Cl^–^ and benzoate in acetonitrile-*d*_3_ displayed a higher binding affinity to (*c*)-**43** (*K*_a_ = 147 M^–1^ and 198 M^–1^, respectively) than to (*o*)-**43** (*K*_a_ = 95 M^–1^ and 80 M^–1^, respectively). The authors reasoned that the increase in binding affinity upon photocyclization is due to preorganization of the binding site as a result of structure rigidification. It is interesting that binding of Cl^–^ is of the same strength as benzoate because the latter is more basic. A possible explanation came from theoretical calculations and ^1^H NMR analysis, which indicated anthracene-CH···Cl^–^ hydrogen bonding in the (*o*)-**43** ⊂ Cl complex, while similar hydrogen bonding interactions were not observed with benzoate as guest.

The group of Bandyopadhyay developed anthracene-appended receptor **44** with two triazole groups as anion binding motifs (Figure 22).^164^ The photocyclization properties of this receptor were similar to those of the other anthracene-containing receptors described above. UV–vis and fluorescence titration experiments in acetonitrile/DMSO (99:1) using F^–^, ClO_4_^–^, CH_3_CO_2_^–^ and benzoate revealed association constants in the order of 10^4^ M^–1^ in the case of (*o*)-**44**. In contrast, addition of these anions to (*c*)-**44** did not cause noteworthy spectral changes, illustrating insignificant binding. The authors ascribed this to reduced flexibility and cavity size in the cyclized form as well as dismantling of the π-system, lowering π–π interactions in the case of benzoate. The binding of benzoate to the *open*-form was studied further by a ^1^H NMR titration and DFT calculations, which confirmed that the triazole protons are the most important in binding of the anion, whereas π–π interactions between the benzoate phenyl group and the anthracene units may also have a contribution. Interestingly, reversal of the photocyclization reaction via irradiation with 254 nm light was enhanced from 43% in absence to 63% in the presence of benzoate, but further investigation is still needed to fully explain this observation.

#### 2.3.6 Miscellaneous Approaches

**Figure 23:**
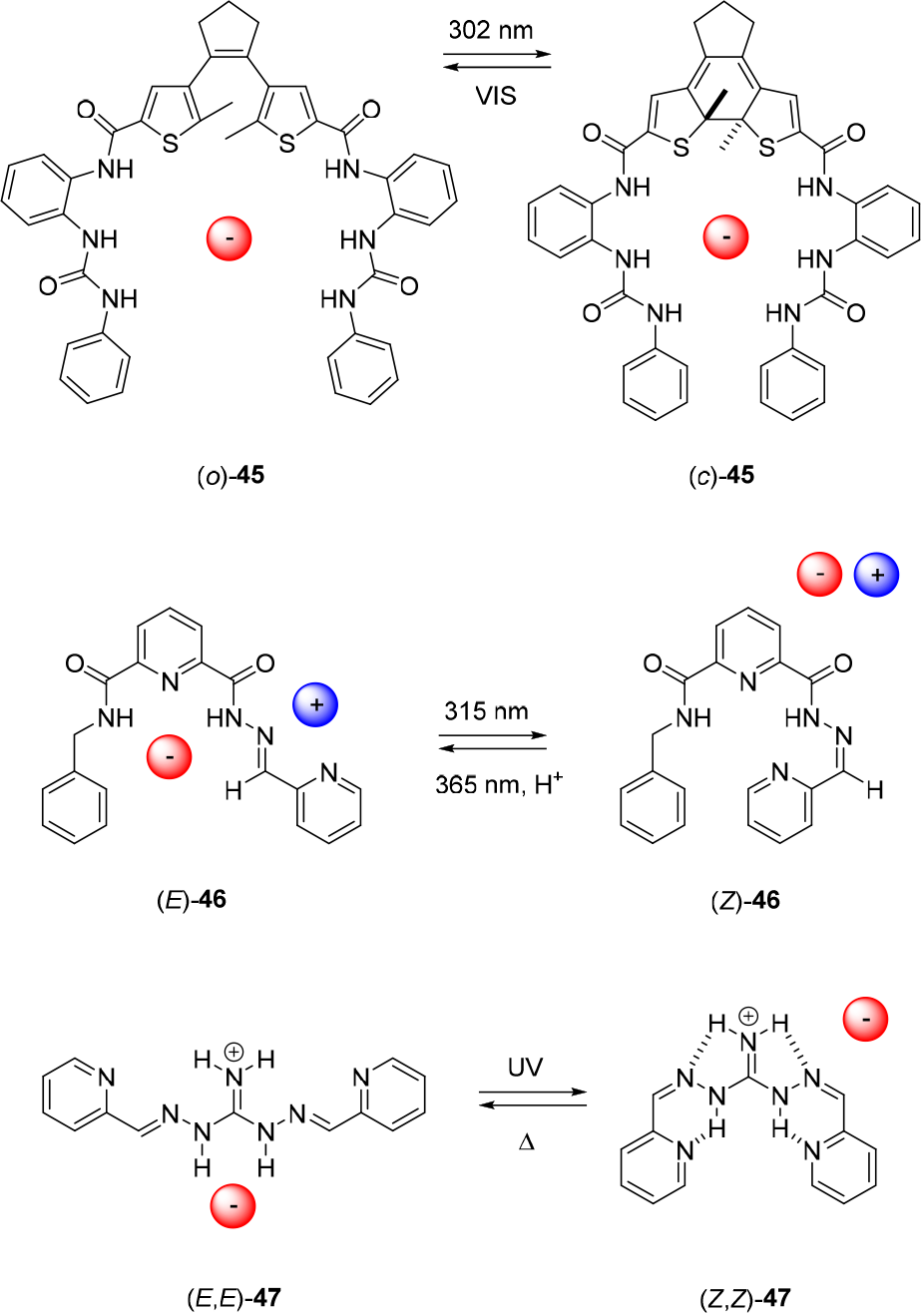
Control of anion binding by dithienylethene- and hydrazone-based receptors.

A small number of other photoswitchable receptors have been developed that do not fall within the other light-based categories. For example, the group of Liu functionalized dithienylethene with amidourea groups to obtain receptor **45** (Figure 23).^165^ Irradiation of the *open*-isomer with 302 nm light afforded the *closed*-isomer in 34% yield and the reverse ring-opening process could be triggered with visible light (>402 nm) to fully reobtain the open-isomer. ^1^H NMR titrations carried out with (*o*)-**45** and (*c*)-**45** in DMSO-*d*_6_ indicated that both isomers were involved in 1:1 complexation with halide anions, but that the former had a slightly higher affinity for Cl^–^ and Br^–^ (*K*_a_ = 68 M^–1^ and 14 M^–1^, respectively) than the latter (*K*_a_ = 58 and 13 M^–1^, respectively), while I^–^ binding was negligible for both isomers. Geometry optimization illustrated that rigidification upon ring-closing enforces the amidourea groups to be slightly further apart, which might explain the somewhat weaker binding to the *closed*-form than to the *open*-form isomer. Nevertheless, the differences in affinity are very small when compared to the receptors based on stilbene or azobenzene, which can be ascribed to the less pronounced geometrical change upon photoisomerization. On the upside, diarylethenes are known to have good fatigue resistance, which is advantageous in the design of robust photoswitchable systems.^166−168^

Kokan and Chmielewski based their design of heteroditopic ion pair receptor **46** on a 2-pyridyl acylhydrazone switch.^169,170^ This receptor could be operated orthogonally by light and pH-change and besides, its anion binding affinity was countercation-dependent Figure 23).^171^ It underwent *E* → *Z* isomerization by irradiation with 315 nm light, and the reverse process could be induced with 365 nm light. In the *E*-isomer, the receptor has a binding site for both an anion and a cation and resultantly, it strongly bound halide/alkali metal ion pairs. In acetonitrile-*d*_3_, the association constants of the ions followed the order: Cl^–^ > Br^–^ > I^–^ and Li^+^ > Na^+^ > K^+^, ranging from 1.58 × 10^3^ M^–2^ for KI to 1.74 × 10^6^ M^–2^ for LiCl. These trends are in agreement with the preference of the *E*-isomer to bind small anions as well as cations with high charge densities. The anion binding site was blocked in the *Z*-isomer by an intramolecular hydrogen bond between the pyridyl nitrogen and the hydrazone N–H atom. Interestingly, the authors found that treatment of (*Z*)-**46** with triflic acid led to nearly quantitative *Z* → *E* back isomerization due to protonation of the hydrazone core to facilitate rotation around the N=C double bond. Where photoisomerization with 365 nm light only gave 42% of (*E*)-**46** at the PSS, addition of triflic acid, then followed by DIPEA as a base to neutralize the solution, could thus be used to recover the (*E*)-isomer. The 315 nm/acid/base switching cycle could be repeated several times in the presence of LiBr going from 100% (*E*)-**46** to 81% (*Z*)-**46**. It is to be expected that this work will stimulate further development of systems that can be operated by multiple, orthogonal stimuli and are targeted at ion-pair binding.^172^

Moyer, Custelcean, and co-workers developed positively charged sulfate-binding 2-pyridyl-diiminoguanidinium receptor **47** that could be switched between its (*E*,*E*)- and (*Z*,*Z*)-isomer.^173^ Photoisomerization from (*E*,*E*)-**47** to (*Z*,*Z*)-**47** with UV light was found to be concentration dependent, resulting in PSS-mixtures of 4:96 (*E*,*E*/*Z*,*Z*) at 1.5 mM and 32:68 (*E*,*E*/*Z*,*Z*) at 10 mM. The (*Z*,*Z*)-**47** enriched mixture thermally relaxed back toward (*E*,*E*)-**47** with an (*E*,*E*/*Z*,*Z*)-ratio of 71:29 at equilibrium. Sulfate binding to the (*E*,*E*)-isomer was investigated by ^1^H NMR and UV–vis spectroscopy in DMSO, which seemed to indicate a mixture of 1:1, 1:2 and 2:1 host/guest binding equilibria with high affinity constants. However, the sulfate binding affinity for the (*Z*,*Z*)-isomer was suggested to be 10^5^-fold lower. Interestingly, the thermal *Z*,*Z* → *E*,*E* equilibrium was influenced by the presence of sulfate, generating an increased amount of 91% (*E*,*E*)-**47**. On the other hand, the photoisomerization behavior was not altered. It was found that an excess of sulfate could be precipitated by the addition of (*E*,*E*)-**47**, while photoisomerization toward (*Z*,*Z*)-**47** led to resolvation of sulfate. The authors show a sulfate binding-release cycle using this concept, which they note could potentially be exploited for sulfate extractions.

Recently, Łukasik et al. reported a photoswitchable receptor based on an embonic acid core, to which two azobenzenes were attached.^174^ Irradiation of the *E*,*E*-isomer with 365 nm light resulted in an estimated total amount of 16% *Z*-isomer (with respect to azobenzene) at the PSS, having a half-life of 22 min and thus converting back to *E*-isomer in the dark. UV–vis titration studies in DMSO using the *E*,*E*-isomer indicated binding of H_2_PO_4_^–^, CH_3_CO_2_^–^, and benzoate in a 2:1 (receptor/anion) fashion, while the PSS mixture enriched with *Z*-isomer was predicted to bind the same anions in a 1:1 stoichiometry.

### 2.4 Other Stimuli Responses

**Figure 24:**
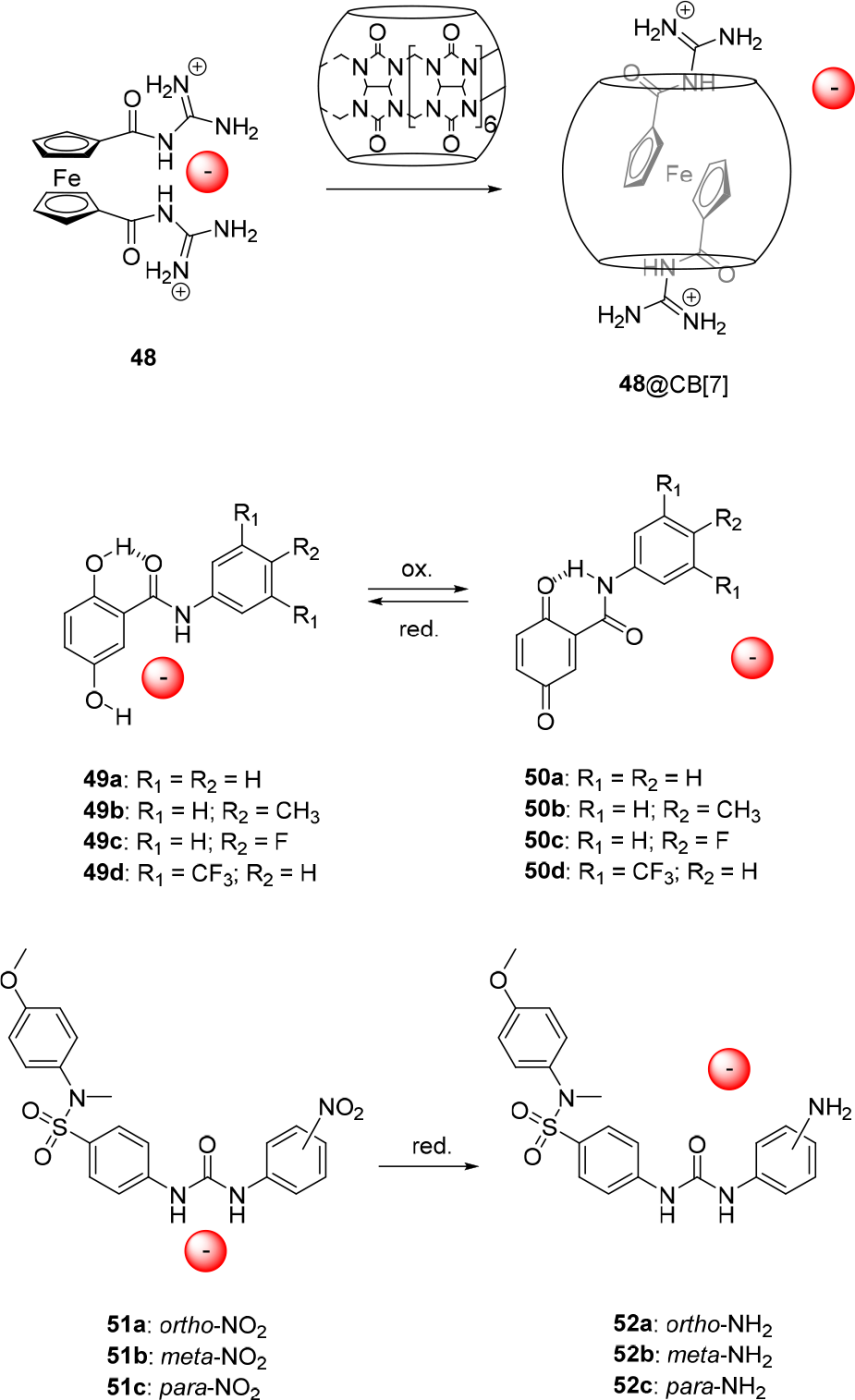
Other stimuli-responsive anion receptors.

A few other strategies, based on different stimuli, have been used to control anion binding affinity. Beck and Winter, for example, designed a system based on cucurbit[7]uril and bis(acetylguanidinium)ferrocene dication **48** (Figure 24).^175^ UV–vis titration experiments showed that the dicationic receptor interacts with a number of dicarboxylate anions in water, among which were phthalate and maleate (*K*_a_ ≤ 10^4^ M^–1^). However, upon the addition of cucurbit[7]uril, which forms an inclusion complex with the receptor that is much more stable than the anion–receptor complex, the anion dissociated.

Other successful responsive systems have been based on redox-control. For instance, the group of Astruc functionalized gold nanoparticles with amidoferrocenyl groups functioning as anion binders.^176,177^ The oxidized amidoferrocenyl groups were able to bind H_2_PO_4_^–^ with high selectivity and an apparent binding affinity in dichloromethane that is 5200 times larger than that of its neutral counterpart. The enhanced binding to the oxidized species was ascribed to charge–charge interactions.

As also seen in acid/base-responsive systems, Gale and co-workers exploited intramolecular hydrogen bonding in redox-responsive receptor **49**, consisting of a hydroquinone motif with various benzamide substituents (Figure 24).^178^ This receptor was envisioned to bind Cl^–^ via two hydrogen bonds, i.e., one with the amide and one with the hydroxyl group. Upon oxidation of the hydroquinone to quinone, anion binding affinity would be reduced due to absence of the hydroxyl hydrogen bond donor and the potential formation of an intramolecular hydrogen bond between the amide and the most nearby quinone oxygen atom. As expected, ^1^H NMR titrations with Cl^–^ showed significantly stronger binding to the hydroquinone receptors (*K*_a(2:1)_ ∼ 336–680 M^–2^ and *K*_a(1:1)_ ∼ 31–191 M^–1^ in acetonitrile-*d*_3_/1%DMSO-*d*_6_) than the quinone analogue (*K*_a(1:1)_ ∼ 11–68 M^–1^ in acetonitrile-*d*_3_. In addition, cyclic voltammetry experiments were carried out, showing good compound stability and reversibility of the oxidation and reduction process.

A related approach was reported by the group of Curõínová using nitro-substituted diarylurea receptor **51** (Figure 24).^179^ Binding studies by UV–vis spectroscopy in DMSO showed that complexation of H_2_PO_4_^–^ and benzoate were strongest to the receptor with the nitro group in *para*-position (*K*_a_ = 3.3 × 10^4^ M^–1^ and 1.7 × 10^4^ M^–1^, respectively). When the electron-withdrawing nitro-group was reduced to the electron-donating amine by reaction with tin(II) chloride in ethanol, the binding affinity for H_2_PO_4_^–^ was reduced 30-fold and for benzoate 19-fold, and this difference could be enlarged to 80-fold by using acetonitrile as the solvent.

The group of Beer very recently reported a redox-responsive telluroviologen and bis(ferrocenyltellurotriazole) anion receptor.^180^ In the neutral or singly oxidized state, anion binding was very weak to negligible for a range of anions (Cl^–^, Br^–^, I^–^, H_2_PO_4_^–^, HSO_4_^–^, and NO_3_^–^) in acetonitrile/water. However, cyclic- and square-wave voltammetry experiments showed that the binding affinities remarkably increased upon oxidation toward the doubly oxidized state, especially for the halides and dihydrogen phosphate, via enhanced chalcogen bonding due to the improved sigma-hole donor ability. The authors note that the voltammetric response in the presence of anions allows these receptors to be used for anion sensing.

### 2.5 Summary

So far, different strategies have been used to develop stimuli-responsive anion receptors, among which allosteric regulation, pH-responsiveness, photoswitching, and redox control are the most thoroughly investigated. Allosteric effects (either positive or negative) are often induced by metal cation binding to a coordinating ligand backbone or calixarene scaffold. It should be noted that here ion pairing may play an additional role in the enhancement of binding affinity. Current examples of redox-controlled receptors are scarce and based on either direct reduction of anion-binding residues, or changes in electron density of attached motifs. Both allosterism and redox control have resulted in significant differences in binding affinity between states, however, in most cases the added effector or the reductant/oxidant causes generation of waste and compromises reversibility, which complicates application in a biological setting.

Better reversibility is achieved in systems that respond to pH change, which was used in the earliest examples of stimulus-controlled anion binding. Here, the anion binding motifs are directly (de)protonated, or acid/base addition causes a structural change in the receptor resulting in a change in affinity. While reversible, the repeated addition of acid and base results in the buildup of waste products. Nevertheless, there is great potential for implementation in transmembrane transport as an endogeneous response to a microenvironment with different pH becomes viable with these systems.

Light-responsive receptors have been developed most extensively and can be operated without the generation of chemical waste. They cover a broad range of structures, among which are molecular tweezers, macrocycles, and foldamers. Several types of molecular photoswitches have been incorporated, including azobenzene, stiff-stilbene, and diarylethene. Furthermore, anthracene dimerization has been exploited. A current drawback is that most of the designs are operated by high energy UV light, which is damaging. Further investigation of receptors that are responsive to visible light would thus be beneficial for advancing the field.

## 3 Controlling Transport Activity

A significant number of (nonswitchable) anion receptors has been shown capable of mediating transmembrane transport by a carrier mechanism, i.e., they act as anionophores.^14−20^ Yet, despite the large number of stimulus-controlled anion receptors available today, their application to control transport processes is still limited. Is there large unexplored potential? The fact that the reported examples that are discussed here have more similarity with nonresponsive transporters as opposed to the receptors from section 2 could indicate that many new designs fail to show transport activity. Nevertheless, not all scientists working on responsive receptor motifs are familiar with the studies that assess transmembrane transport properties, and possible transport activity may therefore have been neglected. We therefore start this section with an overview of the most commonly used transport assays, as an introduction to the nonexpert, and to stimulate further efforts in this direction as stimulus control of anion transport remains a very challenging task.

Where available, we have included half-maximal effective concentration (EC_50_) values as a quantification of transport activity. These values are normally obtained by Hill analysis,^181^ i.e., by measuring the activity as a function of concentration. It should be noted, however, that these values vary with the experimental conditions and methods of data processing chosen. It is thus not always possible to directly compare the activities of compounds between articles.

### 3.1 Commonly Used Assays

To investigate if an anion receptor is able to facilitate transmembrane transport, vesicle-based assays can be performed in which anion efflux (or influx) is monitored using fluorescence spectroscopy or ion-selective electrodes. Typically, large unilamellar vesicles (LUVs, 100–200 nm) are prepared using commercially available phospholipids that are most abundant in biological membranes, i.e., POPC or EYPC.^182,183^ Transport studies using artificial receptors have focused mainly on chloride owing to its important role in biology, and furthermore, it has a relatively low dehydration penalty that allows it to be transported across the hydrophobic bilayer. In general, transport experiments are bound by a couple of simple rules. The transporter will mediate the passive diffusion of ions along a pre-established concentration gradient over the membrane to reach equilibrium. When anions or cations would be moved in only one direction, a charge gradient is built up (which polarizes the membrane), inhibiting further transport before an appreciable change in concentration can be measured.^184^ To maintain the charge balance, either two identically charged ions need to move in opposite directions (antiport), or oppositely charged ions have to be translocated in the same direction (symport). In certain cases, the transporter is selective for taking a single (an)ion across the membrane (uniport) and a second transporter is required to have overall neutral charge transport.

**Figure 25:**
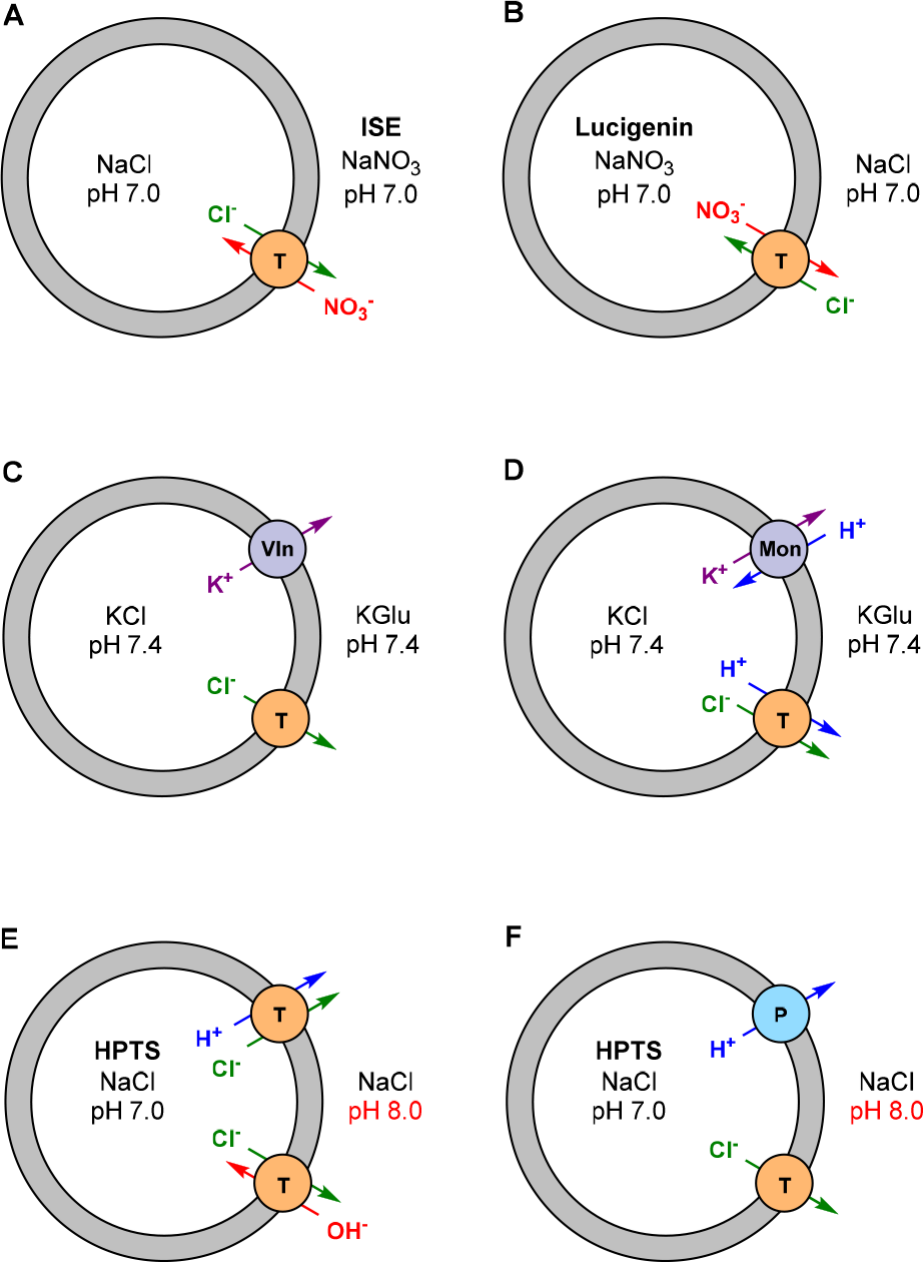
Overview of vesicle-based transport assays: (A) Cl^–^/NO_3_^–^ exchange ISE assay, (B) lucigenin assay, (C) valinomycin-coupled, and (D) monensin-coupled KCl efflux assays, (E) HPTS base-pulse, and (F) protonophore-coupled HPTS base-pulse assays.

Originally, ion-selective electrodes (ISE) were primarily used to measure an increase in concentration of a specific ion during the transport process in the extravesicular solution.^17,185,186^ One of the most widely known methods to monitor chloride transport is the Cl^–^/NO_3_^–^ exchange assay (Figure 25A). Here, the intravesicular solution contains a chloride salt, while a nitrate salt is present outside of the vesicles. Addition of a solution of transporter (commonly in DMSO) to the aqueous vesicle solution can result in incorporation into the hydrophobic membrane, allowing equilibration of both anion gradients via a Cl^–^/NO_3_^–^ antiport process. Because nitrate is more lipophilic than chloride, and thus easier to transport across the bilayer membrane, chloride transport is rate-limiting.^17^ Nevertheless, it has been demonstrated in specific cases, where the binding of the former anion to the transporter is weak, that nitrate transport can become rate-limiting, giving incorrect results when studying chloride transport.^187^

When the group of Davis studied cholapod-based transporters using this assay, they observed no activity because the compounds precipitated when added to the vesicle solution before being delivered to the membrane. To circumvent this problem, they developed the lucigenin assay, which is a variation of the Cl^–^/NO_3_^–^ exchange assay where the transporters are preincorporated in the bilayer during the vesicle preparation (Figure 25B).^188^ Here, LUVs with encapsulated fluorescent dye lucigenin are prepared, which contain a nitrate salt on both the interior and exterior. The transport experiment is started by addition of a chloride salt to the vesicle solution. The resulting chloride influx can be monitored by fluorescence spectroscopy as the emission of lucigenin undergoes collisional quenching with halide ions via formation of a transient charge-transfer complex.^189^

With these Cl^–^/NO_3_^–^ exchange assays, the amount of anion that is transported is measured, but no information with respect to the mechanism by which transport occurs can be deduced. To evaluate whether the compound operates by an electrogenic (i.e., translocation of net charge) or an electroneutral transport process,^190^ cationophore-coupled ISE assays were developed (Figure 25C,D).^191^ Here, the vesicles are loaded with KCl, while the external solution contains a large, hydrophilic anion that cannot pass the membrane, e.g., gluconate or sulfate. In the electrogenic case, the transporter carries only a single ion across the membrane, with concomitant built-up of charge. When it operates through an electroneutral mechanism, the charge is balanced by cotransport of a proton (H^+^/Cl^–^ symport) or exchange with hydroxide (OH^–^/Cl^–^ antiport), affecting both the chloride and pH gradients simultaneously. Transporters are able to operate through one of these mechanisms, or often very likely through both.^192^ By using valinomycin (Vln) and monensin (Mon) as cotransporters, which are naturally occurring cationophores that strictly operate through a different mechanism, one can deduce a preference for electrogenic or electroneutral transport. Valinomycin is a cyclic depsipeptide that is selective for K^+^ transport through an electrogenic mechanism. In combination with an electrogenic anion transporter, this leads to net KCl efflux. Electroneutral transporters, on the other hand, are not able to couple to Vln as the pH gradient generated by the transporter cannot be dissipated. Instead, they can couple to Mon, which is a polyether antibiotic containing a carboxylic acid that is deprotonated upon formation of a pseudomacrocyclic complex with metal cations.^193^ Hence, it is an electroneutral cationophore that strictly operates through a M^+^/H^+^ antiport mechanism, and together with an electroneutral transporter it can give net KCl transport. Electrogenic transporters are not capable of coupling with Mon as there would be no dissipation of a charge gradient. By comparing the rate of transport in the presence of either cationophore, it is possible to determine a preference for one of the possible mechanisms.

A variation on this cationophore-coupled ISE assay is the osmotic assay.^194^ The efflux of KCl decreases the ionic strength of the intravesicular solution and to compensate for this effect, some water is expelled from the vesicle, causing it to shrink and elongate.^195^ In the osmotic assay, this shrinkage is monitored by the increase in 90° light scattering in a fluorimeter and can be directly related to the transport activity of the compound studied.^196^

Probably the most widely used assay to determine transport activity and selectivity is based on the pH-sensitive 8-hydroxypyrene-1,3,6-trisulfonicacid (HPTS) dye, which is encapsulated by the vesicle. In a typical example, NaCl is present in equal amounts in and out of the vesicle, and with the use of a base-pulse (NaOH addition), a pH gradient is created (Figure 25E,F).^197^ In the case of anion transport, this gradient can be dissipated through X^–^/H^+^ symport or X^–^/OH^–^ antiport, causing a change in emission of the fluorescent HPTS dye. This assay is highly versatile as it is can also be used to study cation transport (through M^+^/OH^–^ symport or M^+^/H^+^ antiport), and it can be performed with different types of salt solutions, making it possible to determine selectivity toward a specific anion or cation by comparing transport rates. The process that is being followed is that of electroneutral transport, but to identify possible electrogenic transport, a protonophore can be added, which will couple with the anion transporter leading to net HCl efflux. For this purpose, often the proton carriers carbonyl cyanide-*p*-trifluoromethoxyphenylhydrazone (FCCP) or carbonyl cyanide *m*-chlorophenylhydrazone (CCCP) are used, or alternatively the naturally occurring proton channel gramicidin.^192^ In theory, electrogenic transporters are inactive in absence of a protonophore, however, another possible mechanism to mediate proton transport has to be considered. That is, commercially available lipids contain fatty acid impurities, which by themselves are not capable of proton transport as they cannot pass through the membrane when deprotonated. However, electrogenic transporters that are capable of binding the carboxylate head groups can assist the transport (flip-flop) of a deprotonated fatty acid, resulting in proton transport.^198^ Therefore, to assess the selectivity for electrogenic chloride transport it is recommended to perform the assays in vesicles from which the fatty acids have been removed by treatment with bovine serum albumin (BSA).

### 3.2 pH-Dependent Transport

One of the most commonly used methods to modulate anion transport activity is by changing the pH value. It can either influence the transport mechanism or induce structural changes in the transporter. Given that many artificial anion transporters contain a binding site consisting of hydrogen bond donors,^14−20^ deprotonation and reprotonation of the hydrogen bonding motifs offers control over binding and likely also has an effect on partitioning into the membrane. The main motivation to develop a pH-responsive approach is to enhance transport at pathological sites with different pH profiles. In cancer cells, for example, the extracellular pH is lower compared to healthy cells, generally residing around a value of pH 6.0–6.8, although the whole range between pH 5.6–7.6 can be found.^67,199,200^ Anion influx mediated by artificial receptors may disrupt cellular ion homeostasis leading to apoptosis.^11−13,201^ Hence, the activation of transport in this pH range could potentially be used in anticancer therapies.

A naturally occurring example of an extremely potent chloride transporter is prodigiosin, which has been studied extensively and was shown to operate strictly via an electroneutral H^+^/Cl^–^ symport mechanism.^202^ Moreover, when protonated it can efficiently exchange chloride for other anions such as nitrate and carbonate.^203,204^ Protonation is required for anions to bind, and therefore, it is not surprising that prodigiosin is more active at lower pH values.^205^ Several synthetic analogues, called prodigiosenes, have been developed and showed similar pH dependence, however, for details, we refer to the original papers.^192,205−208^ Interestingly, these synthetic analogues were shown to possess antibacterial and antitumor activity, and while one of the prodigiosenes (i.e., obatoclax) reached phase III clinical trials for cancer treatment, the specific mechanism and role of H^+^/Cl^–^ symport in its antitumor behavior remains unclear.^209^

#### 3.2.1 Binding Site (De)protonation

**Figure 26:**
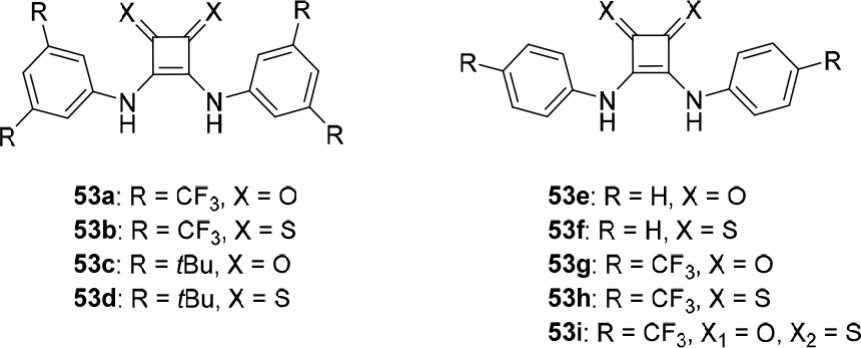
pH-responsive thio- and oxosquaramide transporters.

The groups of Gale and Jolliffe described the first example of pH responsive transport in which deprotonation of the hydrogen bonding motif played a role.^210^ They compared the transport properties of thiosquaramides and oxosquaramides **53a**–**i** (Figure 26), of which the latter had earlier been found to generate higher anion fluxes than, for example, commonly used urea and thiourea-based transporters.^211^ These higher anion fluxes were attributed to the larger binding strength, which in thiosquaramide should be further enhanced as compared to oxosquaramide owing to the more acidic NH protons. Chloride titrations using ^1^H NMR spectroscopy in DMSO-*d*_6_/0.5%H_2_O revealed that **53d** and **53f** had similar or higher binding affinities than their squaramide analogues **53c** and **53e**. However, for CF_3_-substituted **53b** and **53h** the binding constants were surprisingly lower than for oxosquaramides **53a** and **53g** (Table 1). Likely these receptors partially exist in a deprotonated state in DMSO solution, leading to the apparent lower binding affinity. It was determined by a pH-spectrophotometric titration that the p*K*_a_ values for the thiosquaramides lie in between the range of pH 4.9–7.7. It was therefore reasoned that at pH 7.2, the majority of these thiosquaramides exist as negatively charged species, which are unable to bind and, hence, transport anions due to charge repulsion. Indeed, when these compounds were tested in a Cl^–^/NO_3_^–^ ISE assay, limited transport activity was observed at pH 7.2, in contrast to the oxosquaramide analogues, being more active at this pH value. When the same ISE assay was repeated at pH 4.0, transport was enhanced up to 52 times for thiosquaramides **53f** and **53h**, but not for **53b** and **53d**. For compound **53b**, a lower pH value seemed to be necessary because of the high NH proton acidity. However, the assay could not be performed at lower pH because the POPC vesicles that were used become unstable at pH < 4.0. The behavior of compound **53d** was explained by possible aggregate formation, although another reason for the low transport activity of **53b** and **53d**, and likewise for **53c**, was suggested to be their higher lipophilicity with respect to the other compounds tested.

**Table 1:** Chloride Binding Affinity (*K*_a_) and Transport Activity (EC_50_) of Compounds **53a**–**i**^210,212^

compd	*K*_a_ (M^–1^)^a^	EC_50_ at pH 7.2 (mol %)	EC_50_ at pH 4.0 (mol %)
**53a**	643	0.010	0.011
**53b**			
**53c**	145		
**53d**	402		
**53e**	260	1.38	1.42
**53f**	270		<0.013
**53g**	458	0.065	0.077
**53h**	60	0.68	<0.013
**53i**	470	0.22	0.027

a Association constants were determined in DMSO/0.5% H_2_O using ^1^H NMR spectroscopic titrations.

To assess the biological relevance of this work, transport studies were repeated at a wide range of pH values to investigate whether activation could occur around physiological pH (in the range of pH 6–8). In these studies, thiosquaramide **53h** showed better transport activity than oxosquaramide analogue **53g** below pH < 6.0. Furthermore, these studies afforded ‘apparent p*K*_a_’ values (under the conditions of the anion transport experiments), which for thiosquaramides **53f** and **53h** were around p*K*_a_ = 6, while for oxosquaramide compounds they were around 10–11. To obtain a value in between these two extremes, which would be favorable in a biological context, the same groups subsequently developed oxothiosquaramide **53i**.^212^ It was expected to have a p*K*_a_ value lying in between that of analogues **53g** and **53h**, and indeed, its apparent p*K*_a_ was determined as 6.9, indicating that transport could now be switched “ON“ at pH < 7. In a later study, a cationophore-coupled ISE assay provided insight into the transport mechanism. This assay revealed that these anionophores, in their neutral form, are nonspecific and can either act as electrogenic chloride transporter or facilitate H^+^/Cl^–^ symport (or OH^–^/Cl^–^ antiport), depending on the cation transporter.^192^

The group of Elmes also observed pH-responsive behavior in structurally similar amidosquaramide compounds. Protonation of these transporters at a lower pH led to an increase in transport activity.^213^

In a similar study by the group of Chmielewski, pH dependent transport was demonstrated using 1,8-diamidocarbazole transporters with electron-withdrawing nitrosubstituents. In their case, the transporters displayed an apparent p*K*_a_ of 6.4.^214^

**Figure 27:**
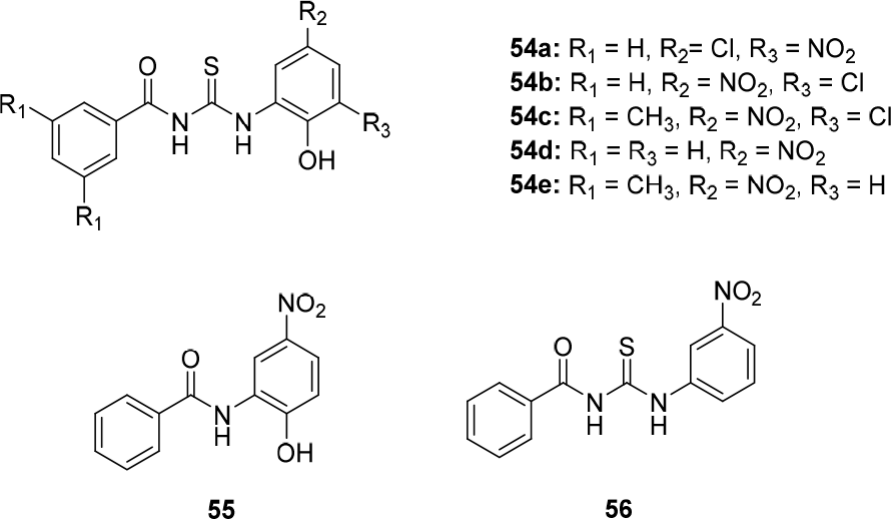
PITENIN-derived pH-responsive transporters and control molecules **55** and **56**.

By high-throughput screening of possible phosphatidylinositol-3,4,5-trisphosphate antagonists, the group of Degterev discovered that a class of chemical compounds named PITENINS had significant antitumor activity.^215^ The group of Manna realized that besides antagonism, an explanation for the observed activity could be their anion transport capability.^216^ Therefore, the PITENINs **54a**–**e** (Figure 27), which are based on acylthiourea containing a phenol substituent, were synthesized, as well as control compounds **55** and **56** that lack the acylthiourea and phenol moieties, respectively. A ^1^H NMR titration experiment with chloride in DMSO-*d*_6_ using **54b** and **54e** revealed that both the acylthiourea and the phenol hydrogen atoms were involved in binding, which overall was found to be weak (*K*_a_ = 22–23 M^–1^). An HPTS assay showed the highest activity for compound **54c** (EC_50_ = 1.25 μM), while also **54b** and **54e** were capable of facilitating transport (EC_50_ = 1.34 μM and 3.26 μM, respectively). The compounds without the acylthiourea and phenol moieties displayed poor activity, highlighting their importance in chloride binding and transport. Additional studies using **54b** and **54e** in the HPTS assay showed an electroneutral H^+^/Cl^–^ symport mechanism. The responsivity to pH change was investigated in a Cl^–^/NO_3_^–^ ISE assay performed at pH 7.2 and 5.5. Remarkably, transporter **54e** was approximately 21 times more active at the more acidic pH, most likely due to protonation of the phenolic oxygen atom (p*K*_a_ = 6.43). Compound **54b** has a more acidic phenol group (p*K*_a_ = 5.02) and did not display any significant difference in transport activity at pH 5.5.

In an MTT cell viability assay, the more potent transporter **54b** displayed more pronounced cell death than **54e**, in accordance with the results reported by the group of Degterev.^215^ When the assay was repeated with buffer solutions that did not contain chloride ions, cell death was still observed, albeit to a lesser extent. This result implies that chloride transport is a factor in the anticancer activity of these compounds, although the binding of these PITENINS to other components of cancer cells may provide an additional mode of action.

**Figure 28:**
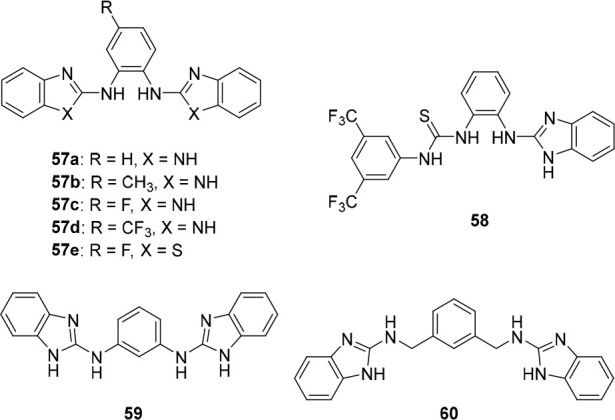
pH-dependent bis(aminobenzimidazole) transporters.

Toward a similar goal, the group of Manna developed bis(aminobenzimidazole) transporters **57**–**60** (Figure 28), which were expected to have increased anion binding affinity after protonation.^217^ Chloride binding studies in DMSO displayed similar 1:1 binding constants for the compounds investigated (*K*_a_ = 16–46 M^–1^) and at pH 7.2, moderate transport activity was observed in HPTS and Cl^–^/NO_3_^–^ ISE assays. The best performing compounds were those with the most acidic N–H protons, i.e., **57c**, **57d**, and **58** having p*K*_a_ values in the range of 6.09–6.18. In addition to proton acidity, the higher activity was suggested to originate from the optimal lipophilicity of these compounds (cLogP values ranging from 5.87 to 6.71) and the high structural rigidity of the substituted *ortho*-diaminobenzene core. When the Cl^–^/NO_3_^–^ ISE assay was performed at lower pH values (pH 5.5 and 6.2), the transport activity for compounds **57b** and **57c** increased. Compared to the measurement at pH 7.2, at pH 5.5 the EC_50_ values of **57b** and **57c** decreased 9-fold from 0.776 and 0.762 mol % to 0.086 and 0.087 mol %, respectively. The results from additional HPTS assays in which FCCP and valinomycin were used as cation transporters led the authors to deduce a H^+^/Cl^–^ symport mechanism at pH 5.5 and an OH^–^/Cl^–^ antiport mechanism at pH 7.2. Interestingly, cell viability studies with a dose-dependent MTT assay showed that both **57c** and **57d** had a 2-fold higher toxicity toward human breast cancer cell lines (MCF-7 and T-47D) than to normal cell lines (BHK-21). A decrease in toxicity was observed when the authors repeated the MTT assay in absence of chloride ions, highlighting the role of H^+^/Cl^–^ symport.

In a similar study, the group of Chen used a structurally related transporter consisting of a central squaramide unit, which was flanked by two benzimidazole substituents. Also in this case, protonation of the substituents at lower pH gave rise to an increase in transport activity, and a higher cytotoxicity was observed toward cancer cells as compared to normal cells.^218^

**Figure 29:**
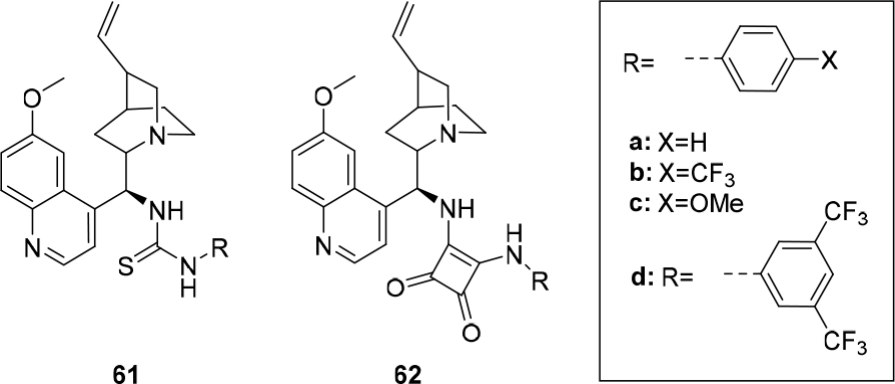
Protonatable quinine-based transporters.

In addition, the group of Manna observed enhanced chloride transport upon protonation of quinine-based receptors **61** and **62**, which were appended with thiourea or squaramide groups (Figure 29).^219^ In the nonprotonated form, squaramide **61d**, having a 3,5-bis(trifluoromethyl) electron-withdrawing substituent, was found to bind chloride the strongest (*K*_a_ = 1033 M^–1^), as was determined by ^1^H NMR titrations in DMSO-*d*_6_. Among the thiourea variants, compound **61** and **62d** with the same electron-withdrawing substituent bound the strongest (*K*_a_ = 80 M^–1^). It was envisioned that protonation of the quinuclidine nitrogen atom (p*K*_a_ = 6.8) would generate a third hydrogen bond donor. Already at pH 7.2, these compounds were efficient transporters, with compound **61a** being the most active (EC_50_ = 1.6 μM), as was demonstrated using a Cl^–^/NO_3_^–^ ISE assay. Lowering to pH 5.5 further increased the transport activity, with the largest increase observed for **61b** and **62b** (5.3 and 8.9 times, respectively). In an HPTS assay, addition of the FCCP protonophore did not affect the transport rate, suggesting an electroneutral H^+^/Cl^–^ symport mechanism. The viability of several types of cancer cells was tested using an MTT assay revealing high activity for **61b** and **62b**, whereas the latter showed lower activity toward normal cells. This activity related directly to the more acidic microenvironment of cancer cells and the pH-dependent transport of these compounds. By additional *in vitro* mechanistic studies, it was demonstrated that the chloride transport plays a crucial role in inducing cell death.

**Figure 30:**
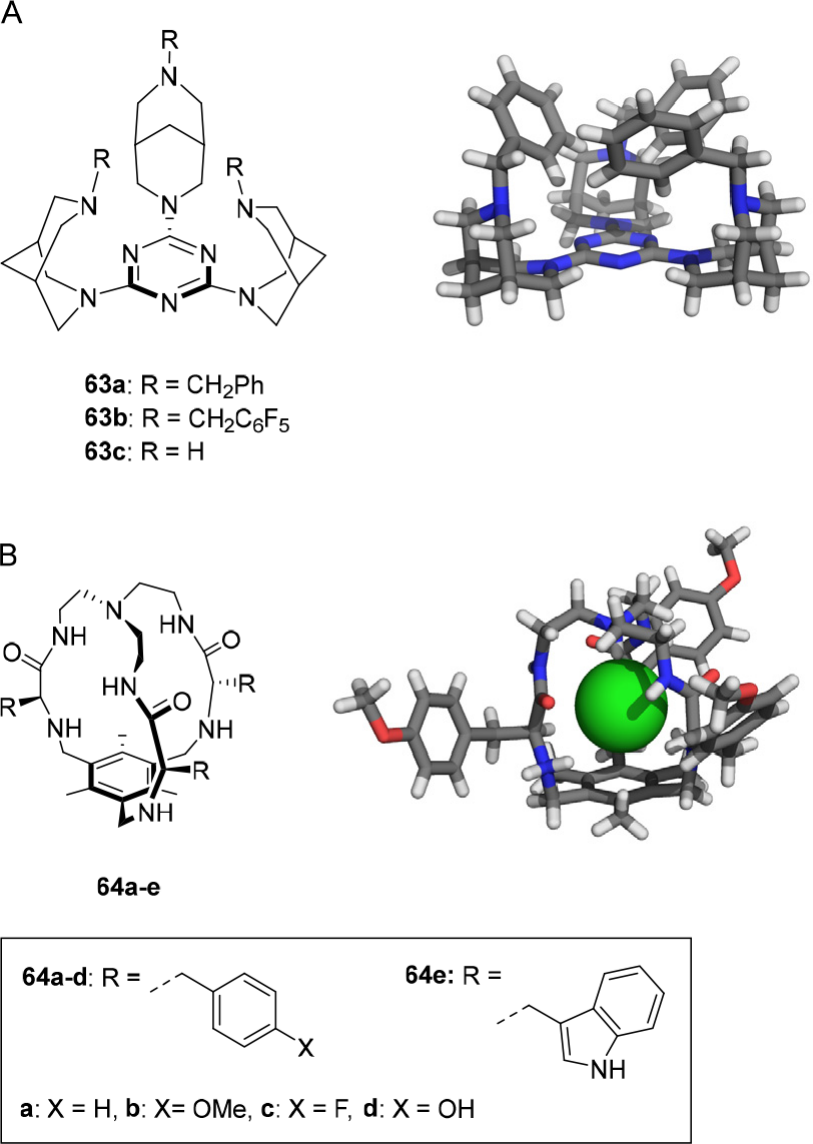
Tripodal (A) semicages and (B) cages and their solid-state structures.

Deviating from the more common bipodal designs, the group of Talukdar designed tripodal semicages **63a**–**c** based on a triazine core with three rigid bispidine arms, which also displayed pH dependent transport activity (Figure 30A).^220^ The hydrophobicity was varied using benzyl (log *P* = 7.44 for **63a**) and pentafluorobenzyl (log *P* = 9.58 for **63b**) substituents and the poorly lipophilic unsubstituted bispidine **63c** was used as negative control compound (log *P* = 1.12). The rigid bispidine arms were envisioned to be preorganized for binding chloride, aided by anion−π and C–H-anion hydrogen bonding interactions with the pentafluorobenzyl unit of **63b** and the benzyl substituents of **63a**, respectively. This preorganization was indeed observed for the benzyl-substituted semicage in the solid state (Figure 30A), however, the pentafluorobenzyl semicage **63b** did not show such preorganization, as instead one of the bispidine arms was located on the opposite side of the triazine core. For the benzyl-substituted semicage, a mass corresponding to the [M + HCl + H]^+^ adduct was observed by ESI-MS analysis, indicating that chloride binding is associated with protonation.

By an HPTS base-pulse assay, the authors demonstrated selectivity for chloride transport over other anions. The benzyl-tethered semicage proved to be the better transporter (EC_50_ = 0.25 μM for **63a** and EC_50_ = 7.14 μM for **63b**), which was explained by the less hydrophobic nature and the higher degree of preorganization. As expected, the unsubstituted semicage **63c** was inactive due to its poor membrane permeability. Further investigation using FCCP and valinomycin illustrated that these semicages operate through a X^–^/OH^–^ antiport mechanism, with preferential transport of Cl^–^ over OH^–^. Assessment of the pH dependence of transport activity using a Cl^–^/NO_3_^–^ ISE assay (at pH values ranging from pH 8.8 to 5.3) showed an optimum for the benzyl semicage at pH 7.0. In contrast, the activity of the pentafluorobenzyl semicage kept increasing with decreasing pH value. To explain this behavior, the p*K*_a_ values of dimethyl benzylamine, dimethyl (pentafluorobenzyl)amine and hexamethyl triazine as model compounds were predicted by calculations (p*K*_a_ = 8.9, 6.7 and 7.0, respectively). On the basis of these calculations, the benzylic amines of semicage **63a** are expected to be predominantly protonated at pH 7.0, where it shows the highest transport activity. The authors reasoned that additional protonation of the triazine core at lower pH values makes the semicage less permeable to the lipid bilayer, resulting in decreased transport activity. With the lower p*K*_a_ value of the protonated amines in the pentafluorobenzyl cage, this optimum must be shifted to lower pH. Interestingly, with these semicages the authors thus demonstrated that pH change can create an anion-binding motif, but that the additional effect on permeability can become detrimental for transport.

In a related approach, the group of Alfonso created tripodal cages **64a**–**e** consisting of tris(2-aminoethyl)amine, appended with amino acids and capped with mesitylene (Figure 30B).^221^ In their protonated form, these tripodal cages all exhibited strong and similar binding affinities (*K*_a_ ∼ 10^4^ M^–1^) for chloride, as was demonstrated by ^1^H NMR titrations in MeCN-*d*_3_/5% H_2_O. Furthermore, the initially formed 1:1 complex was found to interact weakly with a second chloride ion (*K*_a_ ∼ 10^2^ M^–1^). Single-crystal X-ray analysis of the tetra-HCl salts of cages **64b** and **64d** showed that one of the chloride ions tightly bound inside the cage through hydrogen bonding with the protonated 2-aminoethyl unit, among others (Figure 30B). Chloride transport studies using a Cl^–^/NO_3_^–^ ISE assay revealed that the more hydrophobic 4-fluoro-phenylalanine derived cage **64c** is the most efficient transporter and displays the highest increase in transport rate when changing the pH value from 7.2 to 6.2. Further studies with HPTS and lucigenin assays were in line with these results and suggested an acid medium facilitated H^+^/Cl^–^ symport mechanism for cages **64a**–**c**. Interestingly, ^1^H NMR studies with **64c** in micelles of deuterated DPC showed distinct sets of signals for bound and unbound species at pH 7.2 and pH 2.5, where at the more acidic pH the signal intensity for the bound species was much larger, demonstrating the necessity of protonation for chloride binding in the lipid phase. The signal splitting allowed for determination of the exchange rate by EXSY NMR studies, which revealed that the exchange is more than 10 times faster at acidic pH than at neutral pH. Finally, the cytotoxicity of these cages was tested in a MTT assay using a human lung adenocarcinoma cell line (A549). In this assay, a DMEM medium was used with standard (pH 7.5) and slightly more acidic (pH 7.2 and 6.2) conditions. The 4-fluoro-phenylalanine cage **64c**, being the most potent transporter, gave the best results with a 5-fold increase in cytotoxicity when going from pH 7.5 to 6.2, which again demonstrates the potential of pH-responsive transporters as cancer therapeutics.

**Figure 31:**
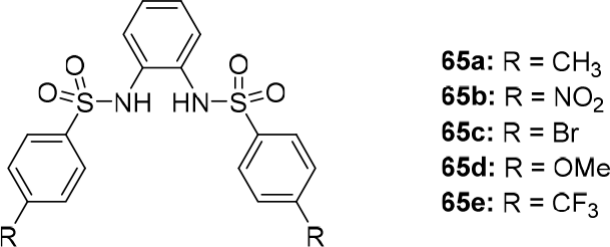
Bis(sulphonamide) transporters with pH-dependent anion/cation selectivity.

Where in the previous examples protonation and deprotonation led to altered transport activity, the group of Talukdar took this concept one step further using bis(sulphonamide) transporters **65a**–**e** (Figure 31),^222^ which were shown to be capable of pH-induced switching between selective cation and anion transport.^223^ Transporters **65c** and **65e**, with log *P* values above 5 and having the highest N–H proton acidity, proved superior at dissipating a pH gradient (interior pH 7.0 and exterior pH 7.8) in an HPTS assay. A similar assay, performed with the most active CF_3_-substituted transporter **65e** (EC_50_ = 10 μM), in which the vesicles were loaded with NaCl and the extravesicular salt was varied (NaX salts: X^–^ = F^–^, Cl^–^, Br^–^, and I^–^; or MCl salts: M^+^ = Li^+^, Na^+^, K^+^, Rb^+^, and Cs^+^), showed minor differences in transport activity for the various anions. However, the activity was strongly influenced by changing the extravesicular cation, being the highest when the potassium, rubidium, and cesium salts were used at pH 7.0 and pH 8.0. Furthermore, from Hill analysis, it appeared that two bis(sulphonamides) bind one potassium ion, and a possible geometry of the (**65e**)_2_@K^+^ complex was optimized by DFT calculations. On the other hand, when the pH was lowered to 5.5, significant selectivity for chloride over other anions was observed, while in this case, a change of the extravesicular cation had no significant effect. This cation/anion selectivity inversion was corroborated by a Cl^–^/NO_3_^–^ ISE assay, showing a marked increase in chloride efflux at pH 5.8 (EC_50_ = 14 μM). This phenomenon was explained by the stronger binding of cations in the deprotonated form at high pH, whereas anion binding is facilitated in the protonated form at low pH.

#### 3.2.2 pH-Induced Structural Changes

**Figure 32:**
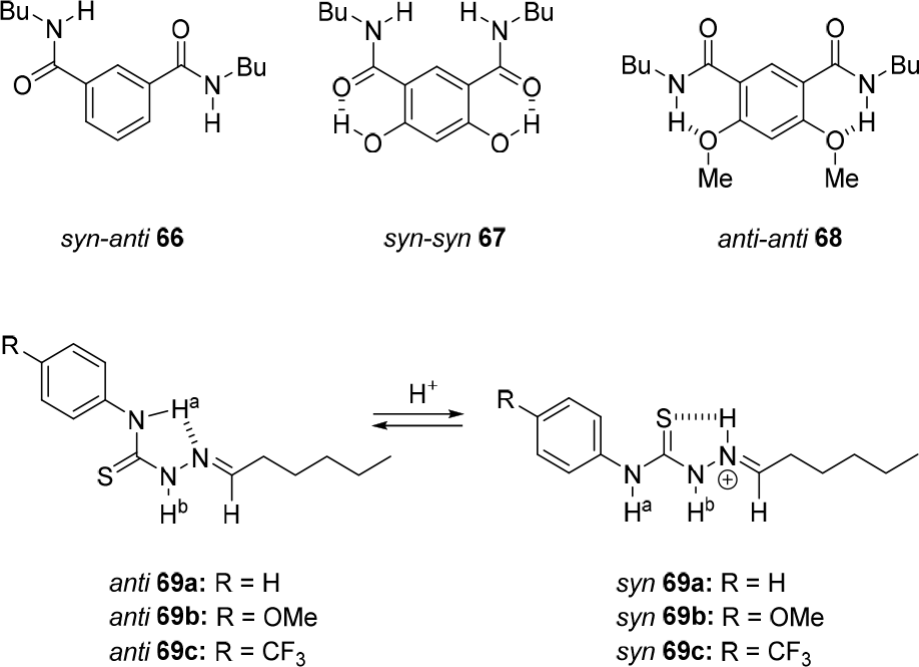
pH-controlled conformations of isophthalamides (top) and thiosemicarbazones (bottom).

In addition to alteration of the anion binding site, protonation and deprotonation can result in conformational changes and thereby influence transport activity. The earliest example of such conformational control, as well as of pH-responsive transporters in general, was reported by the groups of Gale, Davis, and Quesada, who designed isophthalamide-based transporters **66**–**68** (Figure 32).^224^ Importantly, the unsubstituted compound **66** prefers a *syn*–*anti* conformation, but the incorporation of two OH-groups in **67** leads to stabilization of the *syn*–*syn* conformation through intramolecular hydrogen bonding to the amide oxygen atoms. Conversely, methylation of these phenol groups makes the compound adopt a preferred *anti*–*anti* conformation through hydrogen bond formation between the O-ether and amide N–H protons. In the *syn*–*syn* conformation, the amide NH hydrogen bond donors are exposed and have the best orientation to simultaneously bind a chloride ion, which was reflected in the binding constants. These constants were determined by ^1^H NMR titrations in acetonitrile as *K*_a_ = 195 M^–1^ for **66** and *K*_a_ = 5230 M^–1^ for **67**, while binding to **68** was too weak to be quantified.

A lucigenin Cl^–^/NO_3_^–^ assay, initially performed at pH 6.4, demonstrated moderate transport activity for **67** while the other isophthalamides were inactive. When the experiment was repeated at higher pH values, the activity of **67** diminished and no transport was observed anymore at pH 9.1. Based on the p*K*_a_ of the phenol of similar compounds (p*K*_a_ ∼ 8), the authors assumed OH-deprotonation at this pH value. This deprotonation would shift the conformational equilibrium from *syn* to *anti* because of a change in intramolecular hydrogen bonding pattern, which was suggested to be the main cause of the diminished transport activity.

In a later stage, the group of Gale described transporters **69a**–**c** based on a thiosemicarbazone motif that also relied on intramolecular hydrogen bonding to lock the active conformation (Figure 32).^190^ The single-crystal X-ray structures showed the thiourea moiety in the expected *anti*-conformation, stabilized by an intramolecular hydrogen bond between the thiourea N–H^α^ and the imine nitrogen atom. As in the previous case, this intramolecular hydrogen bonding was reflected in very weak chloride binding (*K*_a_ = 16–31 M^–1^ in acetone), much weaker than, for example, thiourea analogues reported by the same group (*K*_a_ = 9.2 × 10^3^ – 2.5 × 10^4^ M^–1^).^225^ The authors reasoned that imine protonation would disrupt the N–H^α^ imine hydrogen bond to give rise to a preferred *syn*-conformation, which in turn is stabilized by an intramolecular hydrogen bond between the iminium N–H and the thiourea sulfur atom. This conformational change was monitored by ^1^H NMR spectroscopy, which upon the addition of HBF_4_ showed signals belonging to the neutral and to the protonated species being in slow exchange on the NMR time scale. In the *syn*-conformation, these thiosemicarbazones will bind chloride more strongly than in the *anti*-conformation, and an additional hydrogen bond with the imine C–H proton may be formed in an overall neutral complex.

A Cl^–^/NO_3_^–^ ISE assay revealed that unsubstituted **69a** and methoxy-substituted **69b** were the most effective transporters at pH 7.2 (EC_50_ = 4.7 and 1.8 mol %, respectively), and an enormous increase in transport activity was observed at pH 4.0 (EC_50_ = 0.0074 and 0.0073 mol %, respectively), i.e, 640-fold in case of unsubstituted thiosemicarbazone **69a**. The trifluoromethyl-substituted transporter **69c** showed similar responsive behavior but was overall less efficient in transport. The differences in activity were attributed to the distinct acidities of the thiourea N–H protons, as well as the basicity of the imine nitrogen, both influencing the strength of the intramolecular hydrogen bond. Finally, a cationophore-coupled ISE assay, in which either monensin or valinomycin was used as cation transporter, revealed strict electroneutral H^+^/Cl^–^ symport behavior. This has similarities with the mechanism by which prodigiosin operates. Hence, this work described the first pH-responsive anion transporter shown to exclusively follow a symport mechanism. The pH-responsive conformational change thus seems a more effective way to control transport activity than (de)protonation of a binding site as was discussed in the previous section, where the largest increase in activity achieved was 52-fold with the thiosquaramide transporter reported Gale and Jolliffe.^210^

At a later stage, the group expanded this class of thiosemicarbazone transporters by introducing various new substitutions. These transporters demonstrated similar pH-activation behavior and also acted through a strict H^+^/Cl^–^ symport mechanism.^226^

**Figure 33:**
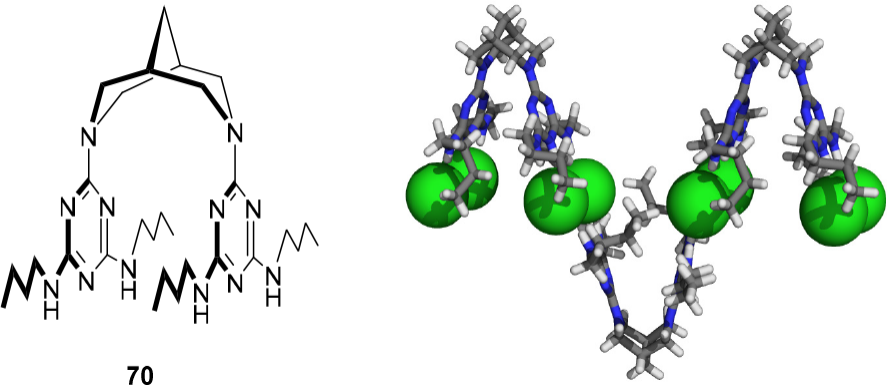
Bis(melamine) bispidine transporter **70** and its X-ray structure obtained upon crystallization with chloride.

Somewhat similar to their tripodal semicage described in the previous section, the group of Talukdar reported bis(melamine) substituted bispidine **70** (Figure 33).^227^ Like the semicage, this compound responded to a pH decrease as a result of protonation of the triazine units, yet the effect on the transport behavior is different. X-ray analysis of single crystals grown in the presence of HCl displayed a zigzag chain of this V-shaped molecule, connected by two chloride atoms at each edge bound to protonated triazine motifs (Figure 33). On the basis of this solid-state structure, the authors proposed transport to occur via a homodimeric [**70**]_2_[HCl]_4_ complex, in line with a Hill coefficient of *n* = 1.91 derived from an HPTS base-pulse assay. Furthermore, Hill analysis revealed that this compound is an effective transporter (EC_50_ = 0.045 mol %). Subsequent cationophore-coupled HPTS assays, using FCCP and valinomycin as cation transporters, indicated a H^+^/Cl^–^ symport mechanism, like in the previous case.

Responsivity to pH change was demonstrated using a Cl^–^/NO_3_^–^ ISE assay carried out at pH 5.8, 7.0, and 8.0. The highest activity was observed at the intermediate pH of 7.0 and according to the authors, a possible explanation is that only at this pH value protonation of the triazine core creates a suitable binding site. At basic pH, the triazine rings are not protonated, rendering the molecule less capable of binding chloride. At acidic pH, on the other hand, a second protonation event of the transporter will widen the V-shape of the molecule due to charge repulsion between the triazine units, and this widening distorts the cooperative binding in the dimeric transporter complex, which was supported with molecular modeling. The found optimum at pH 7.0 is thus ascribed to a combination of the creation of a binding site by protonation and conformational aspects related to charge repulsion.

### 3.3 Photoresponsive Carriers

**Figure 34:**
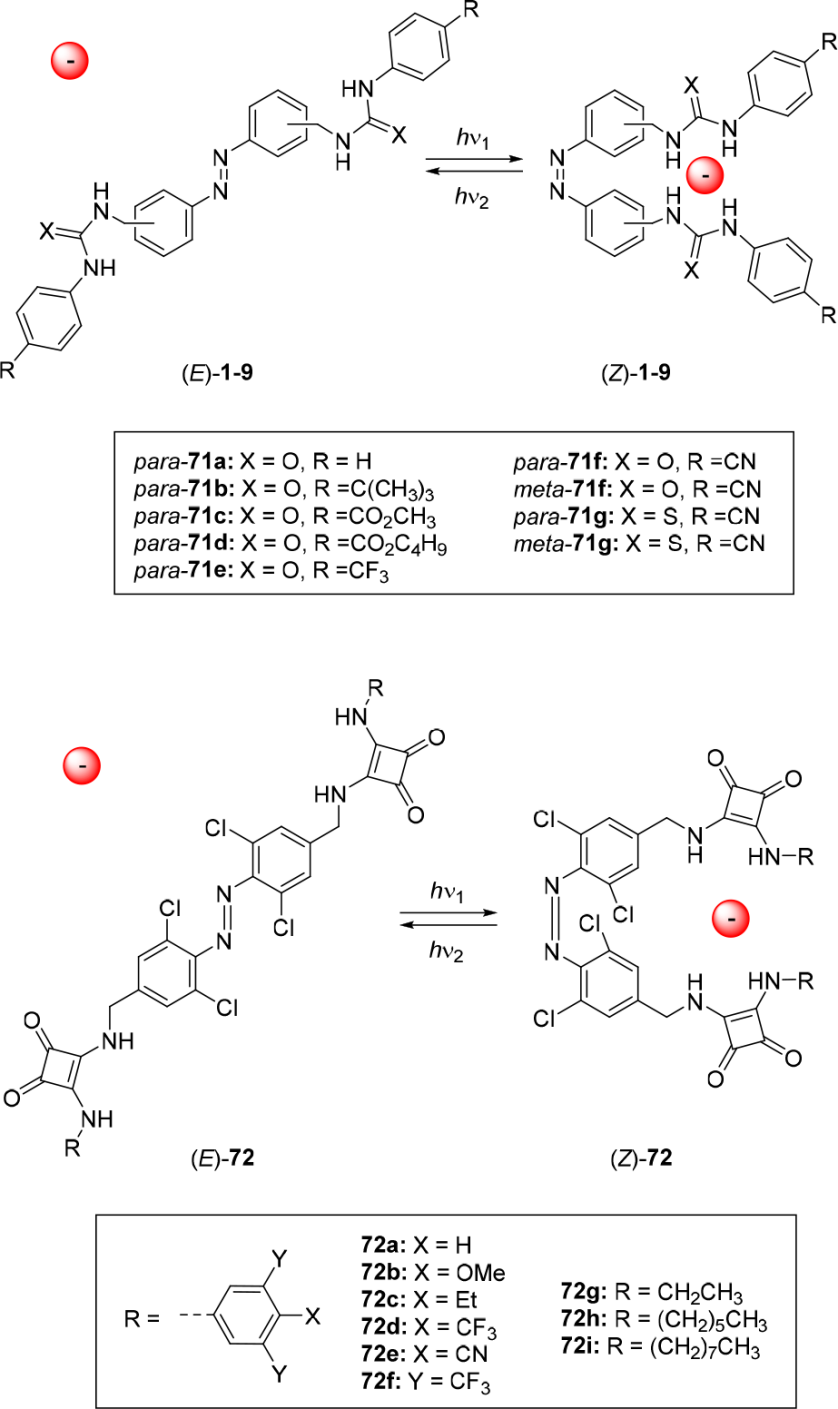
Azobenzene-based bis(thio)urea and bis-squaramides.

Despite the fact that many light-switchable anion receptors have been successfully developed (see section 2.3), relatively few studies in which they have been applied to modulate transmembrane transport are available. In 2014, Jeong and co-workers were the first to report such a study making use of azobenzene derivatives **71a**–**g** with two (thio)urea anion-binding substituents in the benzylic positions (Figure 34).^124^ The highest transport activity was expected for the *Z*-isomer as the ability to simultaneously bind an anion by both (thio)ureas groups would result in a larger binding affinity than in case of the *E*-isomer (see also section 2.3.2). Already in earlier work, the same group had found significantly different chloride transport activities for the *E*- and *Z*-isomers of benzanilide-based bis-urea compounds.^228^ The azobenzene derivatives used here could be converted from the *E*- to the *Z*-isomer by irradiation with UV light (365 nm) in 90–96% yield as was determined by ^1^H NMR studies in DMSO-*d*_6_. The chloride binding affinities, which were determined by ^1^H NMR titrations in 1:9 (v/v) DMSO/CDCl_3_, were the highest for meta-substituted *Z*-azobenzene derivatives **71f** (*K*_a_ = 8400 M^–1^) and **71g** (*K*_a_ > 10^4^ M^–1^), having electron-withdrawing cyano-phenylurea and cyano-phenylthiourea substituents, respectively. Overall, affinity constants calculated for the *Z*-isomers were approximately 1 order of magnitude larger than for the respective *E*-isomers.

Investigation of the transport properties using a Cl^–^/NO_3_^–^ ISE assay revealed very low activities for the *E*-isomers and moderate to good transport activities for the *Z*-isomers, with the cyano-substituted phenyl thioureas *para***-71g** and *meta***-71g** being the most efficient transporters (EC_50_ = 0.19 mol % and 0.15 mol %, respectively). The difference in transport activity between the isomers was too large to be explained by the difference in binding affinity only, and the authors therefore speculated that variance in membrane partitioning, contact area with the lipid surface, and shuttling rates may play an additional role. Importantly, transport could be activated *in situ* by addition of the *E*-isomer to a vesicle solution followed by 365 nm irradiation. To investigate if transport could also be mediated across the cell membrane, Fischer rat thyroid epithelial (FRT) cells were transfected with halide-sensitive fluorescent protein YFP-F46L/H148Q/I152L. Surprisingly, in contrast to what was observed in the liposomal model, only the *meta*-substituted azobenzenes **71f** and **71g** displayed chloride transport in these studies. The discrepancy with the liposomal model studies could either be due to intrinsic differences in cell membrane permeability or interaction of some of the compounds with membrane-embedded proteins.

Recently, the group of Langton reported a similar design, but they used a visible-light-switchable *ortho*-chloro-substituted azobenzene core and squaramide substituents instead of (thio)ureas (Figure 34).^125^ The *E* → *Z* isomerization process was investigated for the parent BOC-protected bis(benzylamino) azobenzene and could be triggered by 625 nm light, affording a PSS mixture with a 20:80 (*E*/*Z*) ratio in DMSO-*d*_6_ as was determined by ^1^H NMR spectroscopy. The *Z-*isomer was found to have a high thermal stability (*t*_1/2_ ∼ 7 days), nevertheless, the reverse *Z* → *E* isomerization process could be induced with 455 nm light, affording 86% of the *E*-isomer. ^1^H NMR titrations in DMSO-*d*_6_ revealed an approximately 3 times larger chloride binding affinity to the *Z*-isomer than to the *E*-isomer of the compounds (*K*_a_ = 142–402 M^–1^ and *K*_a_ = 47–139 M^–1^, respectively), with the strongest binding observed for 3,5-bis(trifluoromethyl)phenyl-substituted **72f**. Investigation of the transport properties using an HPTS base-pulse assay showed a higher activity for the PSS mixtures than for the *E*-isomers, with the largest 8-fold difference for the phenyl-substituted bis-squaramide **72a** [EC_50_ = 0.58 mol % for (*E*)-**72a** and EC_50_ = 0.07 mol % for (*Z*^PSS^)**-72a**], even though this compound did not display the largest difference in binding affinity [*K*_a_ = 90 M^–1^ for (*E*)-**72a** and *K*_a_ = 267 M^–1^ for (*Z*^PSS^)-**72a**]. Therefore, the differences in transport activity were additionally ascribed to a change in mobility as well as chloride encapsulation ability upon *E*/*Z* photoswitching. As in the study by Jeong, also here chloride transport was activated *in situ* in a Cl^–^/NO_3_^–^ ISE assay, however, now reversible control was additionally demonstrated. That is, after addition of (*E*)-**72a** to a solution of POPC vesicles, irradiation with 625 nm light activated transport (50% of the rate obtained upon direct addition of the PSS mixture) and subsequent irradiation with 455 nm light greatly diminished the transport activity.

In a recent study by the same group, synthetic modification of this type of transporter revealed that neither increasing the spacer length between the azobenzene core and the squaramide motif nor changing from *para*- to *meta*- substitution enhanced chloride binding affinity.^126^ On the other hand, an overall increase in transport activity was observed, however, with less differentiation between the two photoaddressable isomers. Additionally, an analogue of **72a** with an *ortho*-fluorinated azobenzene core was made, which by irradiation with 530 nm light afforded a PSS (*E*/*Z*) ratio of 23:77. The transport activity of this variant was enhanced for both isomers by a factor 2 (EC_50_ = 0.25 mol % for the *E* and EC_50_ = 0.03 mol % for the *Z*-isomer) as compared to the nonfluorinated analogue, likely because its lipophilicity is more optimal.

Inspired by previous work from the group of Smith,^229^ Langton and co-workers connected *ortho*-fluorinated azobenzene having only one urea substituent to a phospholipid tail.^230^ In the *E*-isomer, two of these functionalized lipids can facilitate anion transport by a relay mechanism. In the *Z*-form, the tail is shortened, which prevents the relaying action in the bilayer interior. Irradiation with 530 nm yielded a PSS mixture with a ratio of 16:84 (*E*/*Z*), as determined by a ^1^H NMR spectroscopy in DMSO-*d*_6_, and the reverse isomerization is triggered by 405 nm light, giving 95% of the *E*-isomer. An HPTS assay revealed that the *E*-isomer is capable of transport when preincorporated in the bilayer (EC_50_ = 4.5 mol %), and the Hill coefficient confirmed that two molecules are indeed involved, as expected for the proposed relay transport mechanism. Isomerization of the transporter to the *Z*-isomer led to diminished activity, with a ratio of *k*_ini,*E*_/*k*_ini,*Z*_ = 3.0 between the initial transport rates of the two isomers.

**Figure 35:**
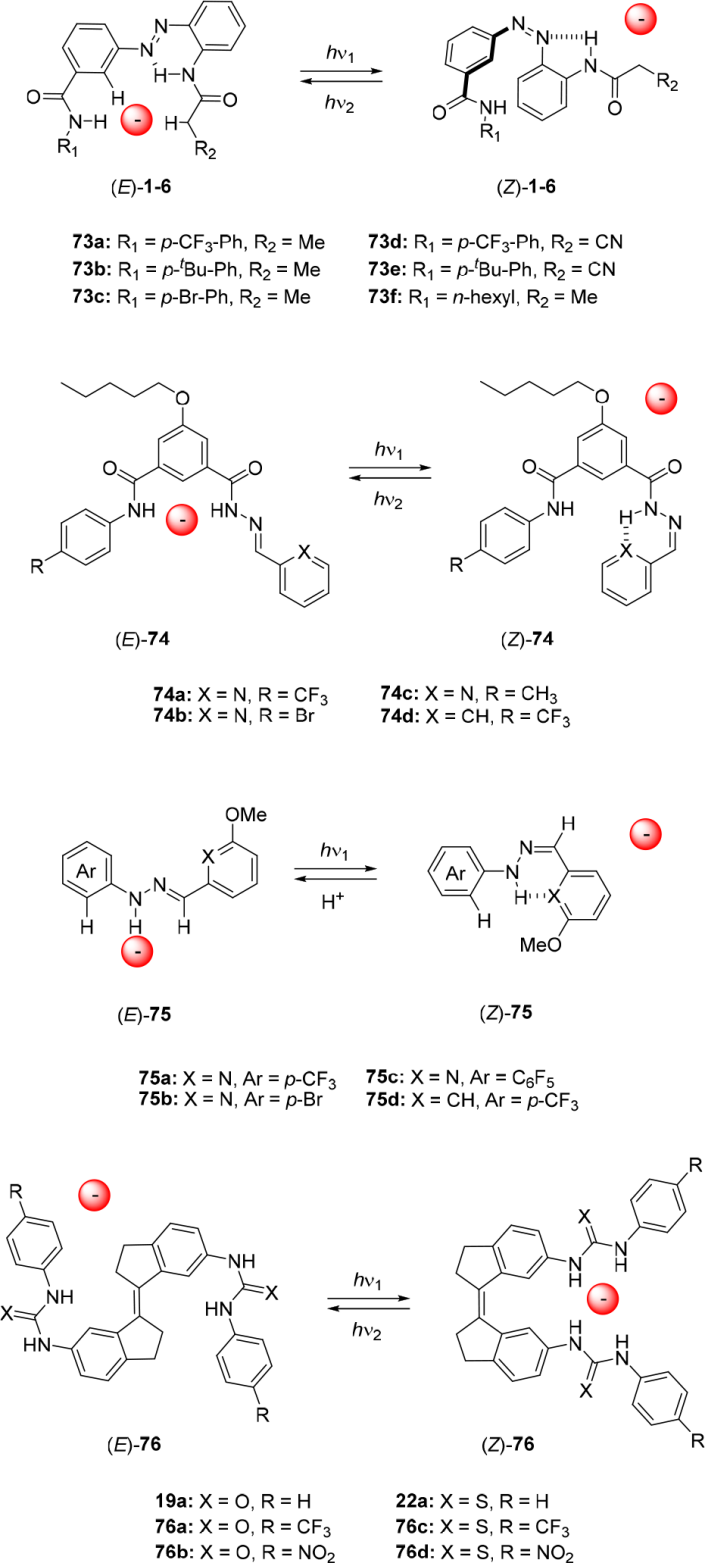
Photoresponsive transporters based on azobenzene, hydrazone, and stiff-stilbene switches.

The group of Talukdar developed azobenzene based transporters **73a**–**f**, which contained an amide substituent in the *ortho*-position on one side and in the *meta*-position on the other side (Figure 35).^231^ Irradiation of the *E*-isomers with 365 nm light in DMSO-*d*_6_ gave 83–90% of the respective *Z*-isomers as was determined by ^1^H NMR spectroscopy and the reverse *Z → E* isomerization occurred thermally (*t*_1/2_ = 32 h) and could be accelerated by irradiation with 450 nm light, as was demonstrated by UV–vis spectroscopy. Opposite to the previously discussed designs, here the *E*-isomer was expected to have the best binding and transport properties, as only in this isomer the amide groups would be able to simultaneously bind the anion. A ^1^H NMR titration in MeCN-*d*_3_ of (*E*)-**73b** and (*E*)-**73e**, as representative examples of the set revealed weaker binding of chloride to the *ortho*-propionamide-substituted compounds than to the *ortho*-cyanoacetamide-substituted ones. That is, (*E*)-**73b** (*K*_a_ = 110 M^–1^) has a lower binding affinity than (*E*)-**73e** (*K*_a_ = 515 M^–1^), which can be explained by the higher NH proton acidity of the latter. The affinity constant for the *Z*-isomer was determined for compound **73b** as *K*_a_ = 88 M^–1^, which is thus slightly lower than for the respective *E*-isomer. By mass analysis (ESI-MS), beside the mass corresponding to the chloride bound 1:1 complex, one corresponding to a dimeric receptor–chloride complex (**73b**)_2_@Cl^–^ was observed, indicating the possibility of additional 2:1 complex formation. Based on DFT calculations, this 2:1 complex was proposed to be of a sandwich-type having the chloride ion hydrogen bonded between two receptors.

In HPTS base-pulse and lucigenin assays, compounds (*E*)-**73a** and (*E*)-**73d** showed the best performance (EC_50_ = 0.198 μM and 0.277 μM, respectively). A Hill coefficient of *n* = 2 was observed for all compounds tested, which indicated that indeed a 2:1 transporter/chloride complex is involved. When the lucigenin assays were performed using the PSS *E*/*Z* mixtures, reduced transport activity was observed, with the largest 17-fold decrease for compound **73c**, even though it is less active in its *E*-form (EC_50_ = 0.419 μM) than **73a** and **73d**, which showed a 6-fold and 3-fold decrease in activity after irradiation, respectively. Given the very small difference in binding affinity between (*E*)-**73b** and (*Z*)-**73b**, also here a change in membrane permeability and mobility should play an important role, as was already suggested for the previously described photoswitchable transporters. Finally, *in situ* control of transport activity was demonstrated in an HPTS assay, where (*E*)-**73a** was added to the vesicle solution, after which applying a base pulse and irradiation with 365 nm light showed diminished transport activity. This activity was (partially) regained when the vesicles were irradiated with 450 nm light prior to applying the base pulse, thus demonstrating reversible isomerization inside the lipid bilayer.

In a later stage, the same group reported 2-pyridyl acylhydrazone-appended isophthalamide transporters **74a**–**d** (Figure 35),^232^ reminiscent of the ion-pair receptor developed by Kokan and Chmielewski (see section 2.3.6).^171^ Similarly, the *E*-isomer was envisioned to bind chloride via the amide and acylhydrazone NH protons, but after isomerization, the binding site would be blocked because of intramolecular hydrogen bonding of one of these protons with the pyridyl group. Irradiation of the *E*-isomer with 312 nm light generated the *Z*-isomer in quantitative yield. Due to stabilization of this photogenerated isomer by intramolecular hydrogen bonding, no thermal back isomerization was observed. Compound **74d**, having a phenyl instead of 2-pyridyl acylhydrazone substituent, lacks this stabilization making *E* → *Z* photoconversion less favorable [PSS_312_ (*E*/*Z*) = 62:38] and leading to completion of thermal back isomerization within a day. Nevertheless, the *E*-isomers of compounds **74a**–**c** could be regenerated by protonating the pyridyl moiety using TFA. Chloride binding was quantified by ^1^H NMR titrations in MeCN-*d*_3_, and the strongest binding was observed for compound (*E*)-**74a** (*K*_a_ = 1.1 × 10^4^ M^–1^), while binding to (*Z*)-**74a** was about 210 times weaker (*K*_a_ = 53 M^–1^).

It resulted from an HPTS assay that **74d** was the most active transporter (EC_50_ = 0.11 μM), followed by **74a** (EC_50_ = 0.62 μM). A Hill coefficient of ∼2 indicated involvement of two molecules in the transport process, in line with the observation in the solid state, where chloride was bound in a **74d**@Cl_2_ complex. When (*Z*)-**74a** was studied in the same assay, an 83% reduction in transport activity was observed. By a lucigenin Cl^–^/NO_3_^–^ assay, it was shown that changing the used extravesicular cation had no effect on the transport rate, which is a striking result given the cooperative cation/anion ion-pair binding observed in the structurally similar receptor reported by Kokan and Chmielewski. Finally, a cationophore coupled Cl^–^/NO_3_^–^ ISE assay revealed that the transport process with **74a** is electrogenic because chloride efflux was enhanced in the presence of valinomycin.

Subsequently, phenylhydrazone-based derivatives **75a**–**d** were investigated (Figure 35),^233^ which function in similar way as the acylhydrazone transporters described above. Chloride can be bound to the *E* isomer through the hydrazone N–H and adjacent protons, as was observed in a ^1^H NMR titration experiment in MeCN-*d*_3_ (*K*_a_ ranging from 30–160 M^–1^). With 365 nm light, *E* → *Z* isomerization was triggered, which leads to blocking of the binding site by intramolecular hydrogen bonding with the adjacent pyridyl group. This hydrogen bond also inhibits thermal *Z* → *E* isomerization, but it nevertheless proceeds after pyridyl protonation using TFA. Transport of these compounds was followed in an HPTS assay, where **75a** showed the highest activity (EC_50_ = 2.5 μM), and with a Hill coefficient of ∼2, it likely involves a dimer. After photoswitching to the Z-isomer, transport activity significantly decreased.

Recently, in collaboration with the group of Gale, we tested the stiff-stilbene bis(thio)urea receptors **19a** and **22a** described in section 2.3.1, as well as their derivatives **76a**–**d** bearing different electron withdrawing groups, in membrane transport assays (Figure 35).^101^ Irradiation of the *E*-isomers with 365 nm light in DMSO-*d*_6_ produced similar amounts of the *Z*-isomers (35–51%) as described earlier for bis-urea **19a**. The reverse isomerization process, induced by 385 nm light, was slightly more favored toward the *E*-isomers of the bis-urea compounds (93%) as compared to the bis-thiourea analogues (74–86%). The nitrophenyl-substituted **76b** and **76d** were an exception to this, as their *E*-isomers could not be regenerated because of poor absorption band separation. ^1^H NMR titrations with chloride in DMSO-*d*_6_ indicated roughly 5–6 times weaker binding to the *E*-isomers (*K*_a_ = 15–21 M^–1^) than to the *Z*-isomers (*K*_a_ = 66–125 M^–1^).

An HPTS base-pulse assay revealed much higher transport activity for the *Z*-isomers than for the *E*-isomers, which were mostly inactive. The highest activity was found for *p*-nitrophenyl-thiourea **76d** (EC_50_ = 0.002 mol %), but the largest difference in activity (of more than 560 times) was observed for *p*-nitrophenyl-urea **76b** [EC_50_ = >10 mol % for (*E*)-**76b** and EC_50_ = 0.018 mol % for (*Z*)-**76b**]. Similar to the previously described examples, the difference in transport activity surpassed the difference in binding affinity for all compounds, again suggesting that distinct membrane partitioning and mobility plays an additional role. Detailed mechanistic studies using protonophore FCCP and by treating the vesicles with BSA (to remove fatty acids) revealed an electrogenic transport process, i.e., the transporters were selective for chloride uniport over H^+^/Cl^–^ symport and OH^–^/Cl^–^ antiport. This selectivity was particularly high for unsubstituted bis-urea (*Z*)-**19a** and bis-thiourea (*Z*)-**22a**, showing 283 and 103 times enhancement of chloride transport, respectively, when this was not limited by H^+^ transport. For the latter compound, this selectivity was further confirmed by monensin and valinomycin-coupled ISE assays, where the anion efflux rate using the latter was the highest. Furthermore, owing to its high transport activity and Cl^–^ uniport selectivity, transporter (*Z*)-**22a** was most effective in membrane depolarization studies (EC_50_ = 0.04 mol %), as was monitored with the membrane potential sensitive fluorescent dye Safranin O. Importantly, this membrane depolarization is accompanied by the generation of a chloride gradient.

Photocontrol over chloride transport was demonstrated *in situ* using an HPTS assay, in which (*E*)-**22a** was added and subsequently irradiated with 365 nm light to give 83% of the activity observed with the (*Z*)-isomer alone. Similar photocontrol was demonstrated using ISE and osmotic assays, where in the latter 365/385 nm irradiation was alternated several times to activate and partially deactivate the transport process. Most strikingly, membrane depolarization and concomitant buildup of a chloride gradient could also be triggered *in situ* by light irradiation, which is somewhat reminiscent of the function of halorhodopsin and anion channel rhodopsin.^234^

**Figure 36:**
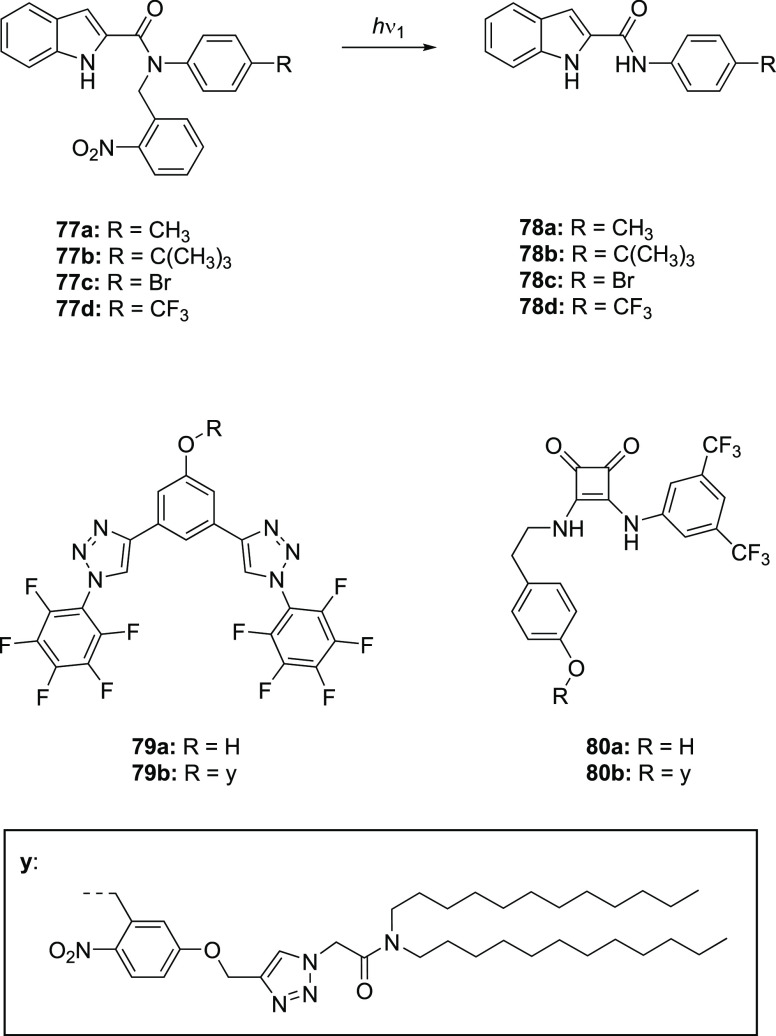
Anion transporters with photocleavable groups.

The group of Talukdar took a different and very simple approach toward photocontrol of transport, albeit not reversible, by equipment of indole carboxamide based transporters with a photocleavable *ortho*-nitrobenzyl group (compounds **77a**–**d**, Figure 36).^235^ The different *para*-substituents on the phenyl ring influenced the acidity of the amide proton (p*K*_a_ = 14.31–13.86) and affected the lipophilicity of the compound (log *P* = 3.60–4.63). Bromo-substituted **78c** showed the strongest binding toward chloride (*K*_a_ = 6.6 × 10^2^ M^–1^) by ^1^H NMR titrations in MeCN-*d*_3_ but displayed only moderate transport activity in an HPTS base pulse assay (EC_50_ = 0.768 μM, 1.23 mol %). The highest activity in this assay was found for the trifluoromethyl substituted transporter **78d** (EC_50_ = 0.219 μM or 0.327 mol %; *K*_a_ = 6.0 × 10^2^ M^–1^). Hill analysis indicated that chloride is bound by two receptors during transport. A possible structure with these two receptors oriented orthogonally around the chloride ion, held together through hydrogen bonding, was minimized by DFT calculations.

The anion binding site was blocked by attachment of the photocleavable nitrobenzyl group, which, moreover, made the compound’s lipophilicity more suitable for membrane partitioning (log *P* = 5.49) than the parent compound, allowing better deliverability. Irradiation with 365 nm light resulted in cleavage of the *ortho*-nitrobenzyl group, as was confirmed by ^1^H NMR spectroscopy in DMSO. The protected transporter itself proved incapable of transport, as was shown in a base-pulse HPTS assay, but transport could be initiated by UV irradiation. In the case of compound **78d**, for example, 91% of the transport activity measured for the unprotected compound was gained after 3 min of irradiation. Subsequently, MCF7 cells were incubated with this compound, and after 365 nm irradiation for 5 min, pronounced cell death was observed for the irradiated cells in an MTT assay. Furthermore, the protected transporter showed enhanced deliverability compared to the unprotected transporter, which precipitated in the extracellular medium and showed no significant cytotoxicity.

In a related approach, the group of Langton used a photocleavable group to trigger a change in carrier mobility in the bilayer.^236^ They modified transporters **79a** and **80a** with long alkyl tails that anchor the receptor to the membrane, preventing the translocation necessary to operate as a carrier. The alkyl tails are connected to the carriers via an *ortho*-nitrobenzyl group that can be cleaved with 365 nm light. This process was followed by ^1^H NMR in DMSO-*d*_6_, where full conversion to the free carriers was observed. A binding titration with ^1^H NMR revealed that both transporters are able to bind chloride, with **80a** showing stronger binding (*K*_a_ > 10^4^ in acetone-*d*_6_: D_2_O 98:2) than **79a** (*K*_a_ = 1 × 10^3^ in acetone-*d*_6_). Transport was investigated in an HPTS assay, where both compounds **79a** and **80a** displayed moderate transporter activity (EC_50_ = 2.7 μM for **79a** and 0.5 μM for **80a**). As expected, the pro-carriers with the membrane anchoring alkyl tails **79b** and **80b** did not display any activity, even at higher concentrations. However, when these compounds were irradiated with 365 nm for 3 min in the bilayer, the transport activity increased. The activity, however, slightly diminished when the active transporter was generated *in situ* when compared to addition of the parent unprotected compounds (EC_50_ = 2.7 μM for addition of **79a** and EC_50_ = 4.1 μM when generated *in situ*), likely because the cleavable group diminishes membrane delivery. Nevertheless for transporter **80b**, a 30% increase in transport kinetics was observed upon cleavage.

### 3.4 Chemically Controlled Partitioning or (De)Activation

As observed in the previously discussed light-based systems, membrane partitioning is a major factor in the ability of receptors to facilitate anion transport. This aspect is particularly relevant for future *in vivo* application because many transporters suffer from poor aqueous solubility leading to precipitation, which is inherent to their desired operation in the hydrophobic lipid bilayer membrane.^237^ For this reason, different research groups have worked on the development of water-soluble procarriers that upon application of a stimulus become more hydrophobic and are resultantly taken up by the bilayer.

**Figure 37:**
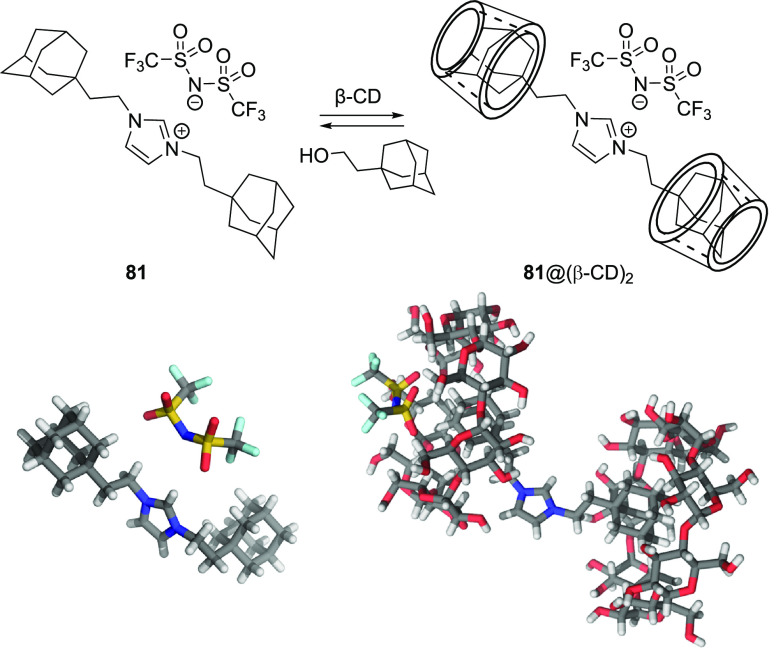
Imidazolium-based transporter encapsulated by cyclodextrin and their respective X-ray structures.

The group of Schmitzer modulated the solubility of adamantyl-functionalized imidazolium salt **81** by taking benefit of its encapsulation by β-cyclodextrin (Figure 37).^238^ This compound acted through a carrier mechanism,^238,239^ while related phenyl-substituted derivatives self-assembled into channels, of which the activity could be controlled in a similar way (section 4.1).^240^ The binding affinity for chloride, as determined by ^1^H NMR titrations in CHCl_3_, was moderate (*K*_a_ = 1356 M^–1^), just as the transport activity (EC_50_ = 17.7 mol %) studied by a lucigenin Cl^–^/NO_3_^–^ assay. The formation of an inclusion complex with β-cyclodextrin was supported by a ^1^H NMR titration experiment in D_2_O/MeCN, in which 1:1 and 1:2 binding was observed (*K*_11_ = 400 M^–1^ and *K*_21_ = 1800 M^–2^), and further confirmed by X-ray analysis of **81**@(β-CD)_2_ (Figure 37). The lucigenin Cl^–^/NO_3_^–^ assay showed a gradual decrease in activity of **81** with increasing amounts of β-cyclodextrin (transport was almost completely inhibited in the presence of 4 equiv). Further, deactivation could be achieved by addition of β-cyclodextrin *in situ*, i.e., during the transport process. In a similar fashion, transport could be activated by adding competitive guests, e.g., 1-adamantaneethanol, which binds β-cyclodextrin much stronger (*K*_a_ = 3000 M^–1^ in D_2_O/MeCN 1:1) than **81**. Similar results were obtained in planar bilayer conductance experiments, where sequential addition of **81**, β-cyclodextrin, and 1-adamantaneethanol led to conductance changes. In a subsequent study, antibacterial activity was demonstrated, while the β-cyclodextrin inclusion complex was inactive.^241^ With the use of 1-adamantaneethanol the antibiotic behavior could be restored, which may allow local activation when the compound is used as drug.

In a later stage, the group of Gale reported (thio)urea and squaramide transporters with a single adamantyl group. These compounds were encapsulated by 2-hydroxypropyl-β-cyclodextrin to enhance water solubility.^242^ By ^1^H NMR titrations in DMSO-*d*_6_/0.5%H_2_O, moderate affinities for chloride were determined (*K*_a_ = 21–249 M^–1^), which correlated with NH-proton acidity. The transport activities (EC_50_ = 0.31–0.95 mol %), which were assessed by a Cl^–^/NO_3_^–^ ISE exchange assay, changed only minimally when the compounds were added as their inclusion complexes in H_2_O as compared to the (normally used) addition as a solution in DMSO. It was thus demonstrated that 2-hydroxypropyl-β-cyclodextrin encapsulation can be used effectively to deliver the compounds from the aqueous phase.

**Figure 38:**
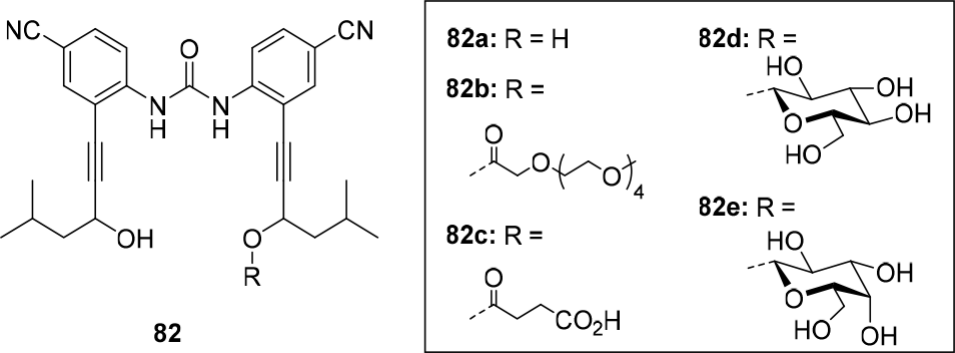
Cleavable urea-based pro-carriers.

The group of Jeong designed compounds **82a**–**e** (Figure 38),^243^ which were based on an earlier developed transporter having a urea binding site flanked by two aliphatic alcohols.^244^ Cleavable water-solubilizing groups were introduced, i.e., tetraethylene glycol, succinic acid, glucose, and galactose, which were connected by ester and ether linkages. These linkages are prone to hydrolysis catalyzed by esterases or lipases and by glycosylases, respectively. Such hydrolysis was envisioned to initiate transport *in situ*, whereas the cleavable water-soluble substituents would enhance deliverability to the membrane. Bond cleavage upon addition of the appropriate esterase, lipase, or glycosylase was confirmed by ^1^H NMR spectroscopic studies, however, it should be noted that the glucosyl- and galactosyl-protected compounds were less susceptible to hydrolysis. Nevertheless, under acidic conditions (pH 5.5), efficient cleavage was observed.

It was observed in a Cl^–^/NO_3_^–^ lucigenin assay that, after incubation with the appropriate enzyme for 30 min, the transport activity significantly increased. In addition, the ability of compounds **82b** and **82e** to facilitate transport of chloride across the plasma membrane was investigated. First, Fischer rat thyroid epithelial cells were transfected with halide-sensitive fluorescent protein YFP-F46L/H148Q/I152L, similar to what was described for the studies using azobenzene-based bis-ureas by the same group (see section 3.3). Transport was initiated when the appropriate enzyme was added. For the galactosyl-protected carrier **82e**, this process occurred only at lower pH (pH 6.0), in line with the ^1^H NMR hydrolysis studies, and is thus selective for acidic microenvironments. Furthermore, in these cell experiments, transport using the procarrier followed by enzyme addition was more efficient than when the active transporter **82a** was added directly, confirming that deliverability is enhanced using this strategy.

**Figure 39:**
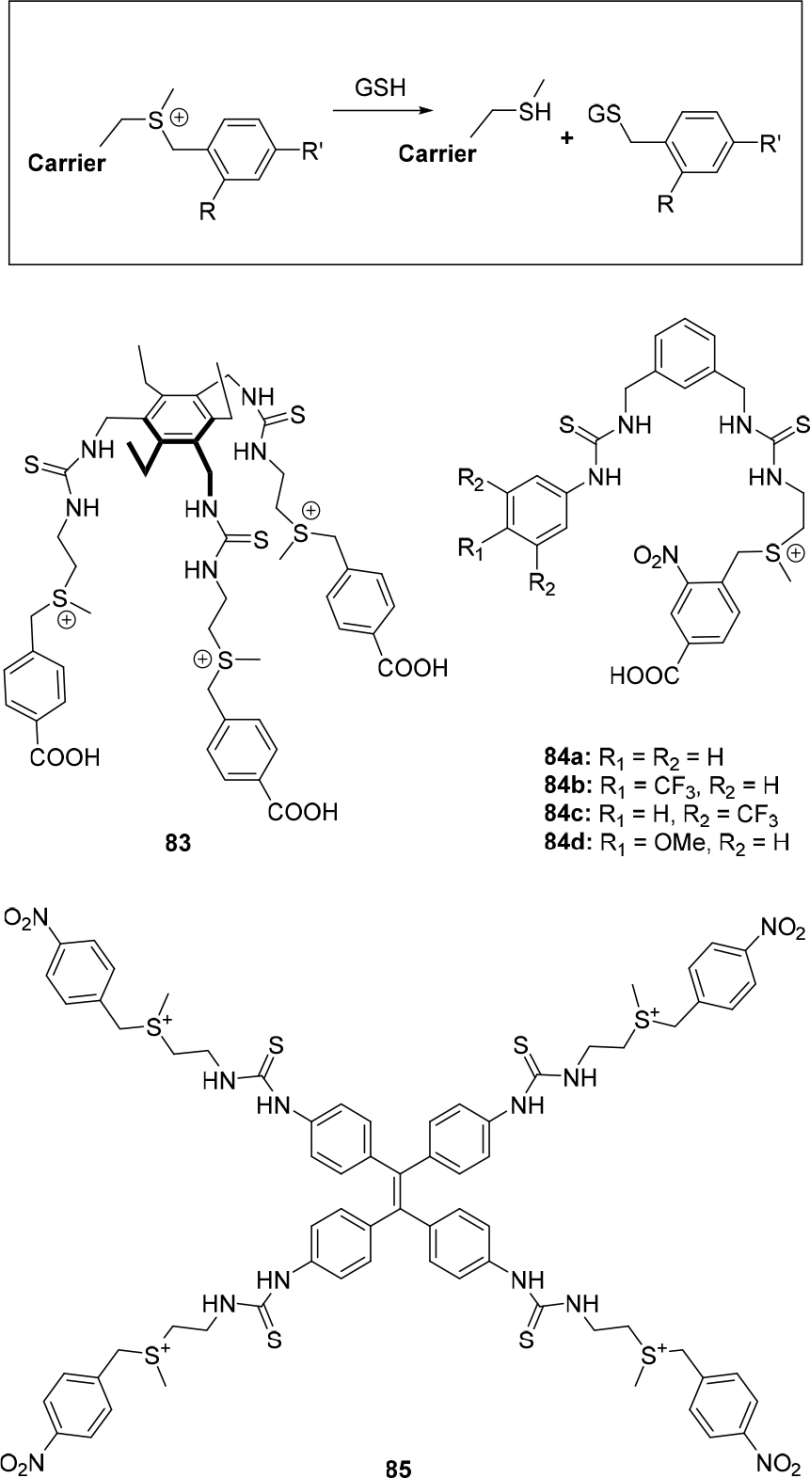
Thiourea-derived GSH-activated pro-carriers.

Toward cell-specific activation, the group of Manna used a protecting group that is cleaved by glutathione (GSH), which is present in elevated amounts in cancer cells.^245^ They synthesized tris-thiourea receptor **83** having charged carboxybenzyl sulfonium end groups (Figure 39). The chloride binding affinity of the unprotected thioether **83** was determined by a ^1^H NMR titration in DMSO as *K*_a_ = 12–21 M^–1^. In the solid state, two receptors were found to form a capsular structure containing two water molecules and two chloride ions. Thioether **83** showed moderate transport activity in an HPTS assay (EC_50_ = 0.14 mol %) and was selective for chloride over other anions. By using FCCP and valinomycin, an OH^–^/Cl^–^ antiport mechanism was deduced. The Hill coefficient was determined to be around 2, indicating that two receptor molecules form an active transport complex, in line with what was observed in the solid-state structure. As anticipated, negligible transport was observed for the protected transporter of **83**, as well as the mono and bis-alkylated intermediates. Cleavage in the presence of GSH was followed by HPLC and turned out to be slow, affording mainly monodeprotected product after 72 h. Nevertheless, when the pro-carrier was incubated with GSH, over the course of several days, transport was gradually activated, indicating that full deprotection occurred in these experiments. Lastly, in an MTT assay, using BHK-21, HeLa, and MDCK cell lines, both the protected and unprotected compound showed low toxicity. Using a MQAE fluorescence assay, the authors were able to demonstrate intracellular transport of chloride ions by **83**, but it was insufficient to promote cell death.

The group of Manna later designed bis-urea pro-carriers **84a**–**d** (Figure 39),^246^ which similarly could be deprotected using GSH, as well as by reactive oxygen species (ROS). Furthermore, owing to the nitro substituent in *ortho*-position to the benzyl sulfonium group, irradiation with UV light additionally caused bond cleavage, somewhat similar to the photocleavable transporters reported by Talukdar^235^ and Langton.^236^ Deprotection in the presence of GSH and ROS, as followed by HPLC analysis, took 72 and 12 h, respectively, whereas photocleavage using 365 nm light was completed in 10 min in DMSO. A ^1^H NMR titration experiment in DMSO-*d*_6_ revealed the highest binding affinity for **84c** (*K*_a_ = 69 M^–1^), which was also the most active transporter, as was determined using an HPTS assay (EC_50_ = 0.04 μM). Again, selectivity for Cl^–^ over other anions was demonstrated, and a Cl^–^/OH^–^ antiport mechanism was suggested. The protected variant of **84c** was indeed inactive, and transport activity similar to the nonprotected **84c** was observed over time by application of all three stimuli.

The same strategy was used for a tetrapodal transporter based on a tetraphenylethene (TPE) core (**85**, Figure 39),^247^ which was also used by Langton and Beer to develop a halogen-bonding receptor as described in section 2.3.1.^114^ In this case, the TPE core was chosen for its aggregation-induced emission (AIE) properties, making compound **85** potentially useful as a bioimaging tool, because bond cleavage would result in aggregation in the cell membrane. The benzyl sulfonium protective group containing a *para*-nitro-substituent was, in addition to GSH, expected to be cleaved by nitroreductase (NTR), which is overexpressed in hypoxic cells. Weak chloride binding (*K*_a_ = 10–19 M^–1^ in DMSO-*d*_6_) and moderate transport activity (EC_50_ = 0.03 mol %) was observed by ^1^H NMR titrations and a Cl^–^/NO_3_^–^ ISE assay, respectively. In the ISE assay, coupling with valinomycin was observed, indicating electrogenic chloride transport. While the protected **85** was inactive in transport studies, bond cleavage facilitated by both GSH and NTR led to an activity gain, although like in the previous designs, cleavage was slow (>72 h). Only for the unprotected **85** AIE was observed in DMSO by increasing the water content, while in the bilayer an enhancement of the emission was observed, showing that the simultaneous functioning as transporter and bioimaging tool is indeed viable.

**Figure 40:**
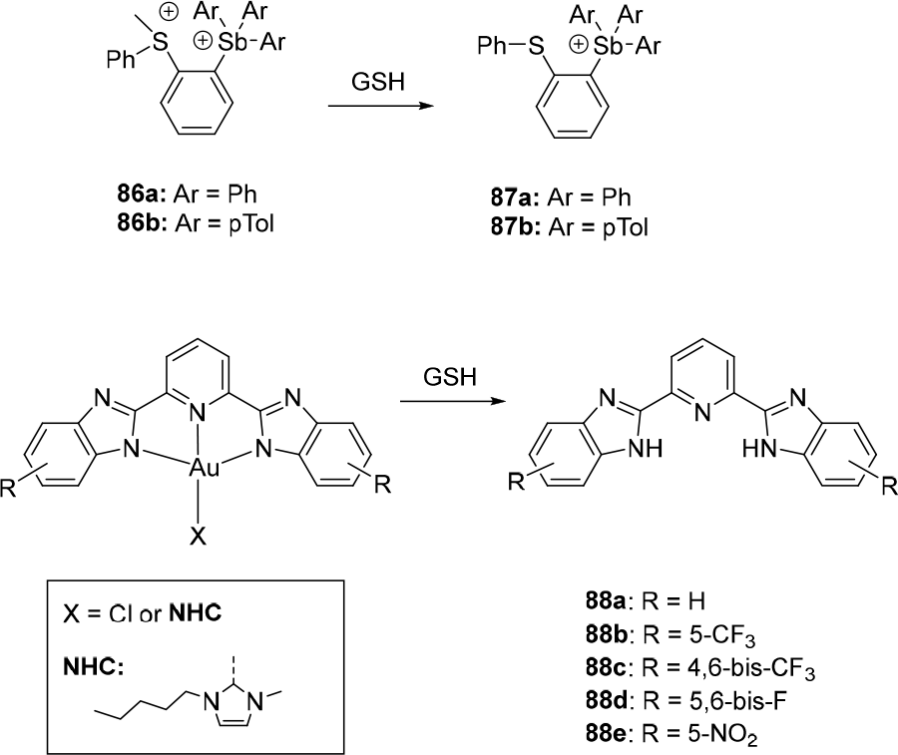
Binding site modification by GSH treatment.

The group of Gabbaï used a similar GSH-cleavable sulfonium motif as the group of Manna, which in this case was involved in anion binding.^248^ They developed dicationic receptors **86a** and **86b** able to bind chloride through both a chalcogen bond with the thioether and through a pnictogen bond with the stibonium substituent (Figure 40). These compounds were found to bind chloride strongly (*K*_a_ > 10^6^ M^–1^) as was investigated using a UV–vis titration experiment in MeCN. The DFT-optimized geometries of the chloride complexes supported the anticipated binding mode. The monocationic receptors **87a** and **87b** had a much lower binding affinity (*K*_a_ = 4.40 × 10^3^ M^–1^ and *K*_a_ = 2.34 × 10^3^ M^–1^, respectively), in line with a less-pronounced antimony-centered σ-hole character compared to **86a** and **86b**, in addition to the singly charged nature. In the solid-state structure of **87a**, a short S–Sb distance (within the sum of van der Waals radii) was observed, indicating an intramolecular interaction.

In a valinomycin-coupled ISE assay both **87a** and **87b** showed moderate chloride efflux (EC_50_ = 4.7 mol % and EC_50_ = 0.6 mol %, respectively). The higher activity of the latter compound was ascribed to a difference in lipophilicity (log *P* = 6.12 for **87a** and log *P* = 7.68 for **87b**), as there is only minor difference in binding affinity. In contrast, the dicationic receptors were virtually inactive, likely because of the high hydrophilicity of these compounds (log *P* = −0.46 for **86a** and log *P* = 1.26 for **86b**). An additional explanation was sought in the excessively high binding affinity, which could prevent release of the anion. By ^1^H NMR spectroscopy, it was determined that in the presence of three equivalents of GSH, **86b** was fully reduced to generate **87b** after 90 min at 37 °C in D_2_O/DMSO-*d*_6_ (8:2 *v*/*v*). Activation of transport was then demonstrated in the same valinomycin-coupled ISE assay, where incubation of **86b** at 37 °C for 20 min with increasing amounts of GSH, showed a gradual increase of transport activity.

Very recently, the group of Langton reported a tellurium-based transporter, of which the transport activity was controlled by redox stimuli. The tellurium center could form a chalcogen bond with chloride, which was the strongest when the transporter had a formal oxidation state of Te(IV) (*K*_a_ = 935 M^–1^). In this state, the molecule is an active transporter (EC_50_ = 45 nM), with a modest selectivity for electrogenic chloride transport. In contrast the Te(II) or Te(VI) analogues did not display any activity, providing redox control over the transport process. That is, transitioning between Te(II) and Te(IV), either by oxidation with an hydrophobic peroxide or by reduction with DTT activated and deactivated transport, respectively.

With the same goal of activating transport in the presence of GSH,^249^ Gale and co-workers used the liberation of Au^3+^ ions from the 1,3-bis(benzimidazol-2-yl)pyrimidine anion binding site in **88a**–**e** upon reduction (Figure 40). The uncomplexed compounds possessed moderate chloride binding affinities (*K*_a_ = 63–132 M^–1^) as determined by ^1^H NMR titration in DMSO-*d*_6_. The most active transporters were **88b** and **88c** as determined by ISE Cl^–^/NO_3_^–^ exchange and HPTS assays (i.e., EC_50_ = 0.42 and 0.49 mol % from ISE and EC_50_ = 0.10 and 0.094 mol % from HPTS, respectively). In mechanistic studies using cationophore-coupled assays, all compounds were found capable of both Cl^–^ uniport and H^+^/Cl^–^ symport, where proton transport was remarkably fast and likely occurs via deprotonation of the NH groups. The Au(III) complexes of **88b** and **88c**, ligated to either chloride or *N*-heterocyclic carbene (NHC), were prepared, and they could be effectively reduced by DTT, GSH, and TCEP to generate the active metal-free transporter as was studied by UV–vis and fluorescence spectroscopy in DMSO. The carbene complexes turned out to be more labile than the chloride complexes, and Au(**88c**) was reduced faster that Au(**88b)**. In a liposomal model, reduction using the neutral DTT was much faster than with the charged GSH and TCEP, suggesting that this process occurs in the bilayer membrane. Chloride transport was initiated upon reduction, however, some activity was also observed for the Au(III)-complex of **88c**, which is likely capable of mediating transport by coordination of Cl^–^ to the metal center. The cell viability in the presence of transporters **88a**–**e** and their complexes was investigated in cancerous (SW620) and noncancerous (HEK293 and MCF10A) cell lines. Here the carbene complex of **88b** and the chloride complex of **88c** showed the most promising results, where in the noncancerous cells they have limited toxicity, yet in the cancerous cell line, a high cytotoxic effect similar to the parent metal-free transporter was observed after reduction.

### 3.5 Summary

Similar stimuli have been used to control transport activity as described for binding affinity in the previous section, i.e. pH change, light, and redox agents. However, no examples of allosterically controlled systems are available. The earliest reports of switchable transport make use of variations in pH value, which could be of relevance in cancer treatment as tumor cells have a different pH profile compared to healthy cells. Initial designs mainly focused on (de)protonation of the hydrogen bond-donating motifs, but more advanced designs have been reported recently where the protonation event induces a structural change in the molecule. Overall, the observed changes in transport activity are primarily explained by an altered binding mode and affinity. Interestingly, although protonation of the receptor can also have a major impact on its solubility in the membrane, this aspect is rarely investigated.

In contrast to modulation of binding affinity, for which light was predominantly used, pH responsiveness takes up the largest portion for switchable anion transport. Nevertheless, control by light has been achieved, most often with tweezer-type receptors. Surprisingly, even though the differences in binding affinity between the two photoaddressable states is small in these examples, relatively large differences in transport activity have been observed, which suggest an additional role for changes in membrane solubility and mobility. In a different approach, pro-carriers containing a photocleavable group, which influenced anion binding and partitioning or mobility have been synthesized. With light, transport mediated by these compounds was successfully activated, but the process is irreversible.

Most of the systems that respond to chemical stimuli do not undergo a change in binding strength but instead make use of a change in membrane partitioning. They have cleavable groups installed that solubilize them in aqueous media, where after cleavage they are delivered to the membrane. In most examples, these pro-carriers can be cleaved with glutathione, which is present in elevated amounts in cancer cells. Attempts to control binding affinity by chemical stimuli in transmembrane transport systems remain scarce, but promising first examples have recently been reported.

## 4 Channel and Pore Formation

The transport of anions can not only be mediated by receptor molecules that operate as carrier, but also by anion recognition motifs that form channels or pores in the membrane without disrupting its integrity. The main strategy employed toward responsive channels is to control their supramolecular self-assembly in the membrane, whereas also a few examples of blocking pores in metal–organic and cavitand-based channel-forming structures are known. Although various types of stimuli-responsive channels and pores have been described in the literature, many of them do not discriminate between ions or are cation-selective. These fall outside the scope of this discussion and have been reviewed elsewhere.^250−253^ Here, we exclusively focus on stimuli-responsive channels for which anion selectivity has been demonstrated.

### 4.1 Molecular Self-Assembly

**Figure 41:**
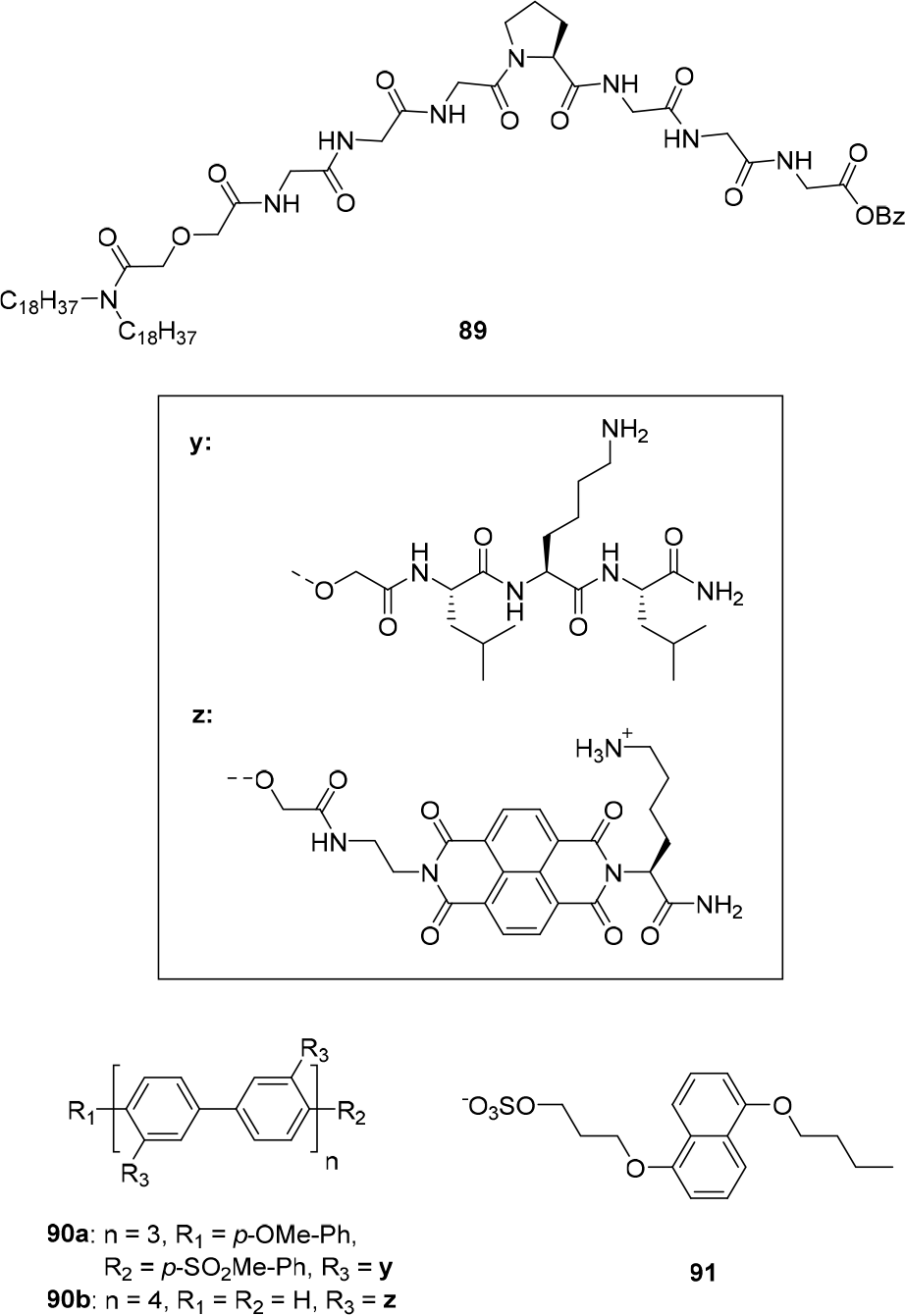
Building blocks that self-assemble into voltage-gated channels.

Many proteins are known to respond to a change in membrane potential (voltage gating). One of the first studies that described voltage-gating of anion transport through an artificial (self-assembled) channel came from the group of Gokel.^185^ They designed lipid mimic **89** (Figure 41), which contains a polar heptapeptide headgroup as well as a hydrophobic dialkylamino-group as the membrane anchor. The heptapeptide contained a central proline residue to induce a bend in the structure to adopt a conformation through which anions were able to flow. In the bilayer, two molecules were predicted to dimerize into a channel. Ion exchange (Cl^–^/NO_3_^–^) and planar bilayer conductance assays demonstrated the formation of channels having >10:1 Cl^–^/K^+^ selectivity. Most importantly, the channel exhibited longer opening times at higher voltages. In a later stage, the same group investigated various analogues of this compound aiming at enhanced transport activity, however, the voltage gating behavior was not studied further.^252^

Around the same time, the group of Matile developed oligophenylene-based rigid rods (**90a**, Figure 41), containing push–pull substituents and lateral peptides, which could assemble into β-barrel-like structures.^254,255^ Upon applying high membrane potentials, these rods oriented in a parallel fashion, which amplifies the dipole and creates the active structure. Transport assays using small unilamellar vesicles containing HPTS and Safranin O, to simultaneously monitor changes in pH and potential, showed that the transport activity increased with increasingly polarized membranes. Further, it was deduced that transport occurred via an anion-selective OH^–^/Cl^–^ antiport mechanism and, while varying the external cation did not have any influence, the rate of transport was anion-dependent and decreased in the order I^–^ ≥ Cl^–^ ≥ OAc^–^ ≥ F^–^, following the Hofmeister series. In addition, concentration-dependent studies supported a tetrameric assembly and planar bilayer conductance studies confirmed single-channel characteristics. The ion selectivity in the latter studies was consistent with that in the unilamellar vesicle assays, with permeability ratios (*P*_Cl–_/*P*_K+_) between 2.7 and 3.7.

**Figure 42:**
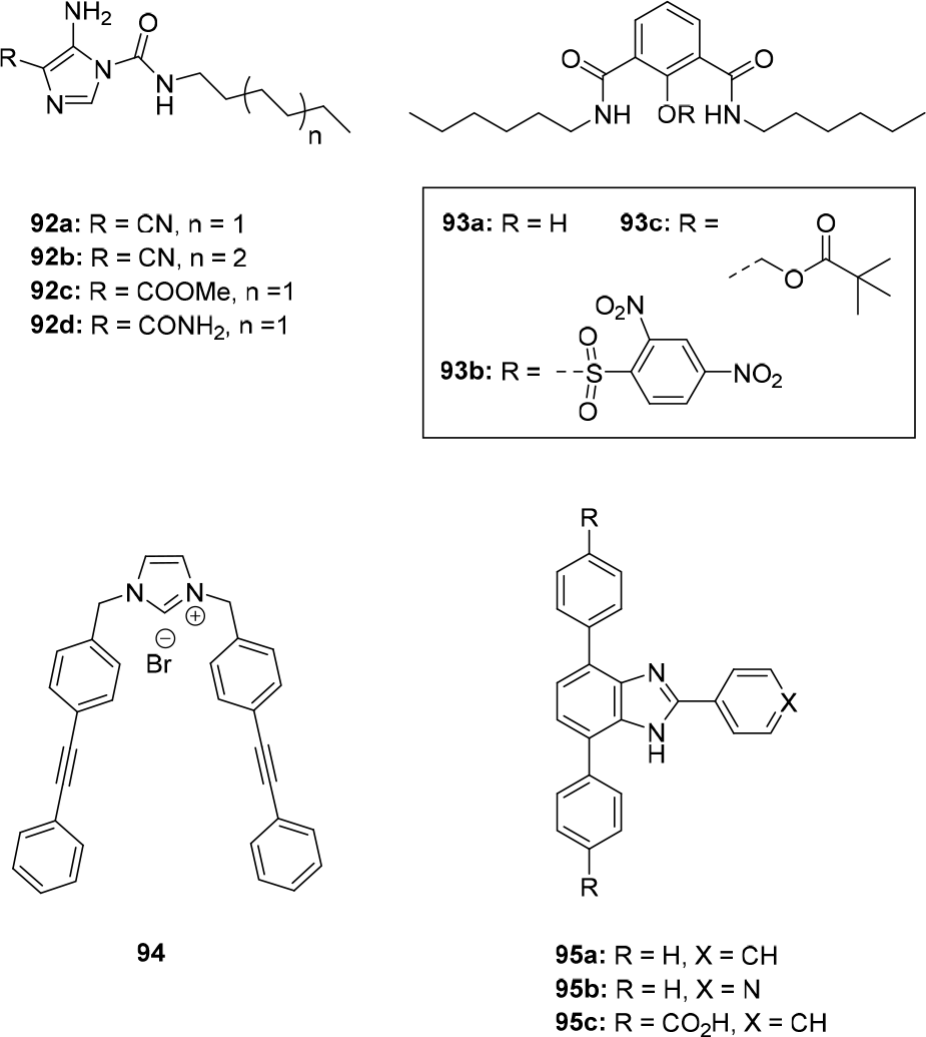
Chemically responsive channel-assembling molecules.

**Figure 43:**
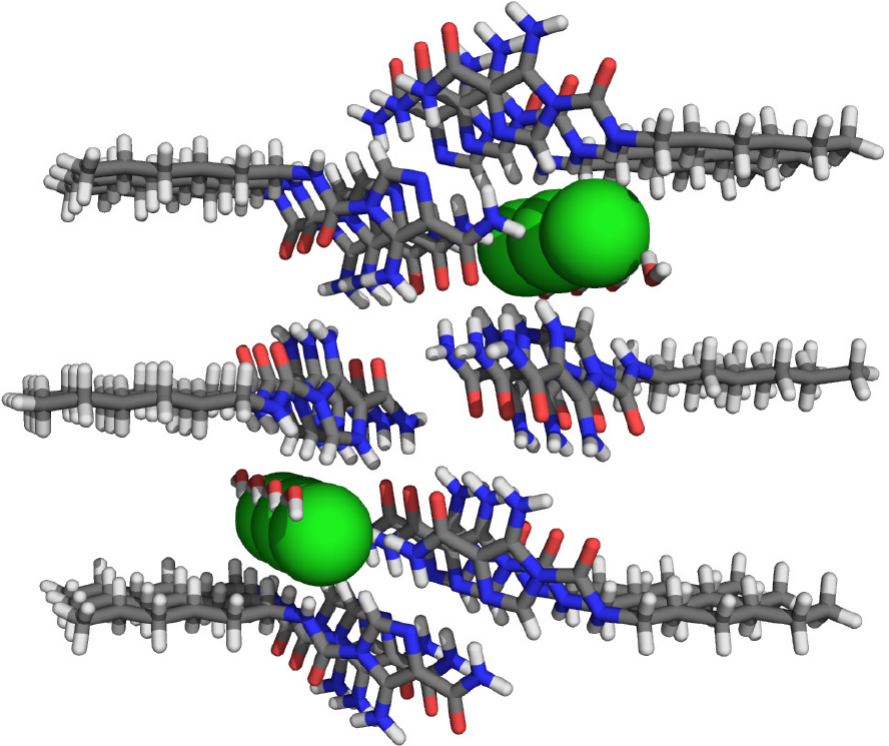
Single crystal X-ray structure of **92d**•HCl showing pore formation.

Barboiu and co-workers assembled channels from amino-imidazoles **92a**–**d** (Figure 42), which responded to changes in pH, in addition to voltage.^256^ Single-crystal X-ray structures of **92c** and **92d**•HCl showed layered hydrogen bonded networks, leaving pores filled with Cl^–^ and H_2_O (Figure 43). On this basis, the authors considered Cl^–^ channel formation in the bilayer, which was confirmed by HPTS and patch-clamp assays. The highest activity was observed at the lower and higher pH values tested (pH range 7.4–11.4), with a minimum at around pH 8.4. The authors reasoned that at the lower pH value, partial protonation of the amino-imidazoles would induce a Cl^–^/H^+^ symport mechanism, while at the increased OH^–^ concentration at the higher pH would favor Cl^–^/OH^–^ antiport. Importantly, no significant changes in Cl^–^ transport behavior were found upon varying the alkali metal countercation in the extravesicular solution, corroborating anion selectivity. In addition to the remarkable pH responsiveness, a very large increase in transport activity was observed at higher voltage in planar lipid bilayer experiments.

Following up on their earlier work, the group of Matile reported activation of a synthetic ion channel by a small-molecule effector.^257^ The same oligophenylene rod as before was now functionalized with naphthalenediimide (NDI) (**90b**, Figure 41). This rod dimerized into an inactive “closed” helical structure via NDI-NDI π-stacking interactions. Addition of dialkoxynaphthalene (DAN) **91**, however, caused a conformational change as a result of the formation of NDI-DAN charge-transfer complexes, resulting in an “open” barrel-like structure. In contrast to when compounds **90b** and **91** were used alone, significant transport was observed after their mixing (in an HPTS assay), which was facilitated by an anion-selective OH^–^/X^–^ antiport mechanism. Transport studies at various concentrations of **91** revealed an EC_50_ value of 13.7 μM and a Hill coefficient of 6.5, indicating that at least six DAN molecules need to form a complex with **90b** to activate the ion channel. More detailed studies on this system using patch-clamp techniques confirmed the selectivity for anions, showing a permeability ratio (*P*_Cl–_/*P*_K+_) of 1.38.^258^ From the single-channel conductance (of 94 pS), an inner diameter of 3.5 Å was calculated, which was in line with what was expected based on geometry optimization for a tetrameric barrel-like assembly having 12–16 incorporated DAN ligands. Formation of the initial helical structure and its unwinding upon addition of DAN was corroborated by circular dichroism and fluorescence spectroscopy. Titration studies in which compound **91** was added to rod **90b** in vesicles showed that a helix-to-barrel transition occurs when 1–3 equiv are added, while up to 7 equiv can be added to maintain the “open” barrel-like structure. It was noted that more equivalents could diminish transport activity due to spatial restriction of the central cavity.

The group of Talukdar used glutathione (GSH) to activate an isophthalamide building block, with a 2,4-dinitrobenzenesulfonyl (DNS) cleavable protecting group, for self-assembly.^259^ The parent unprotected **93a** (Figure 42) exhibited transport activity, as demonstrated by an HPTS assay (EC_50_ = 6.54 μM). Transport was proposed to occur via an M^+^/Cl^–^ symport mechanism and was selective for KCl. Channel formation was corroborated by planar bilayer conductance studies and the rate of Cl^–^ transport was found to be higher than that of K^+^ transport (permeability ratio *P*_Cl–_/*P*_K+_ 50:1). On the other hand, the DNS-protected analogue **93b** did not show significant transport activity but, nevertheless, addition of GSH to cleave the DNS protecting group *in situ* led to activation of transport to reach an efflux rate similar to that of **93a**. Remarkably, the cytotoxicity toward MCF-7 cancer cells, known to have an elevated concentration of GSH, was higher for **93b** than for **93a**. The authors suggested that the former compound could be more membrane permeable and is thus better delivered to the membrane, prior to its conversion to the active **93a**. In a following study, the same authors prepared methyl pivalate-protected derivative **93c**.^260^ In this case, the protecting group could be cleaved by an esterase instead of GSH to yield the active channel-forming compound, as was again shown using liposomal and biological assays.

As described for carriers in section 3.4, altering membrane partitioning is a viable strategy to modulate transport properties. For channel-assembling molecules, it has been applied as well by the group of Schmitzer.^240^ In an earlier stage, they had observed that stacking of aromatically substituted imidazolium salts is influenced by complexation with cyclodextrin hosts.^261,262^ The same strategy was applied to channel formation using imidazolium compound **94** (Figure 42). Its capability of transporting Cl^–^ was demonstrated in a lucigenin assay. Molecular modeling showed that aggregates, formed via π–π stacking interactions, match the width of the lipid bilayer and could potentially act as channels with a positively charged interior. This assembly mode was supported using fluorescence spectroscopy, by which excimers were observed after incorporation into liposomes. The possibility of forming inclusion complexes with either α-cyclodextrin or cucurbit[7]uril was demonstrated with ^1^H NMR and UV–vis spectroscopy and, moreover, the fluorescence emission of **94** within liposomes changed upon addition of these macrocycles, as a consequence of its extraction to the aqueous phase. As envisioned, in a lucigenin assay, chloride transport was shown to be inhibited by adding 1–2 equiv of these macrocyclic hosts. In a later study, the same authors expanded on this concept using adamantyl-functionalized imidazolium salts that were shown to operate as carriers (section 3.4).^238^

### 4.2 Metal–Organic Structures

The group of Schmitzer showed that benzimidazole **95a** is able to self-assemble into anion-selective channels,^263^ and later that modification with pyridyl and carboxylic acid groups (**95b** and **95c**, respectively, Figure 42) allowed formation of active transporters upon complexation with Pd^2+^ ions.^264^ This complexation was first monitored in both organic solution and the lipid bilayer by UV–vis and fluorescence spectroscopy. Interestingly, addition of Pd(OAc)_2_ to **95b** and **95c** in DMSO resulted in gelation. Scanning electron microscopy (SEM) on the gels showed bundled fibers and based on molecular modeling, the aggregated structures were envisioned to be able to form membrane-spanning channels. By a Cl^–^/NO_3_^–^ ISE exchange assay, an up to 2-fold increase in (modest) activity was observed upon *in situ* addition of PdCl_2_ to prior-delivered **95b** and **95c**, whereas in the case of **95a**, the activity remained virtually the same. Interestingly, when preformed metal–ligand complexes were used, no increase in activity with respect to the ligands alone was found, which led the authors to speculate that the structure disassembles in the aqueous external vesicle solution. Indeed, when PdCl_2_ was added afterward, the higher activity was restored. Finally, inhibition of bacterial growth of Gram-positive wild-type strain *Bacillus thuringiensis* (HD73) was demonstrated upon PdCl_2_ addition.

**Figure 44:**
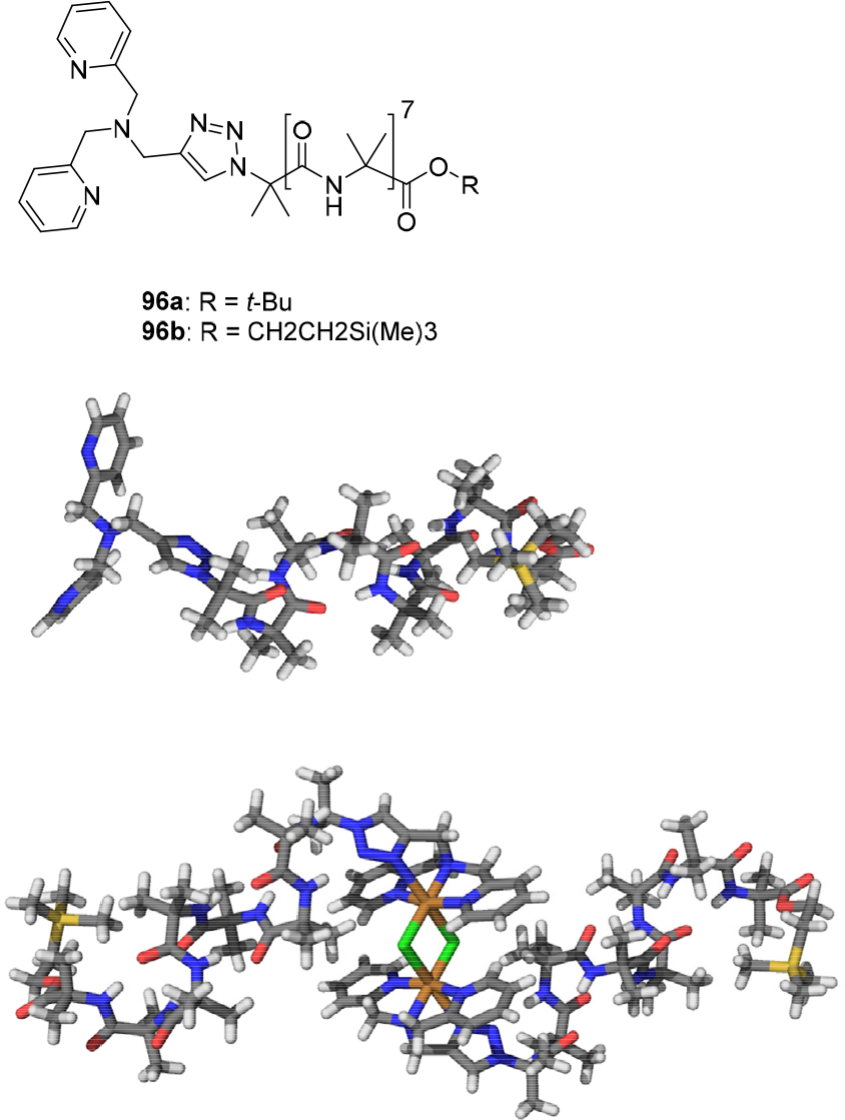
Metal-chelating α-aminoisobutyric acid foldamers, including X-ray structures of **96b** and the dichloro-bridged copper complexes of **96b**.

Webb and co-workers developed octameric α-aminoisobutyric acid foldamers **96a** and **96b** with a metal chelation moiety attached to the N-terminus (Figure 44), which allowed activation of transport by the addition of CuCl_2_.^265^ Single-crystal X-ray analysis of **96b** showed a monomeric 3_10_ helical structure with a length of 2.1 nm in the absence of copper, whereas in its presence, dimers with a total length of 3.5 nm were observed. The copper complexes of **96a** and **96b** revealed 5-to-8 times higher activities than the foldamers alone as was determined in HPTS assays. Furthermore, transport could be activated *in situ* by adding CuCl_2_, where subsequent addition of a competing (EDTA) ligand caused deactivation. Although some differences in transport rates were observed by varying the countercation of Cl^–^ (Li^+^, Na^+^, K^+^, and Rb^+^), the largest differences were found when the anions were varied (Cl^–^, Br^–^, I^–^, NO_3_^–^, and SO_4_^2–^). A carrier-like transport mechanism was excluded by U-tube experiments and the channel-forming capability of the most active complex Cu(**96b**)^2+^ was confirmed by planar lipid bilayer experiments, while no conductance was observed using parent **96b**. Last of all, the antibiotic activity of **96a**, **96b**, Cu(**96a**)^2+^, and Cu(**96b**)^2+^ was estimated using *BacillusMegaterium* strain DSM 319. The minimum inhibitory concentration of the copper complexes was found to be 3–4 times lower than that of the corresponding copper-free foldamers.

**Figure 45:**
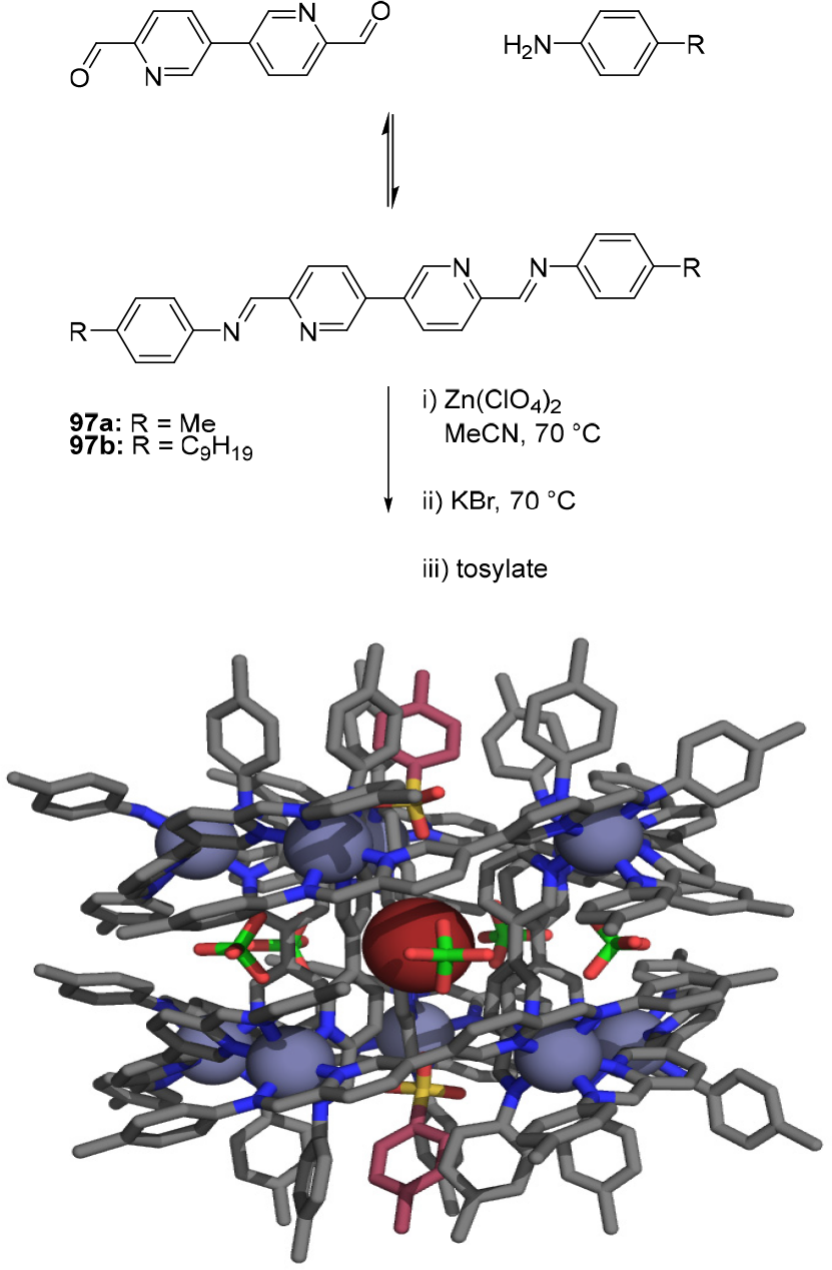
Self-assembly and single-crystal X-ray structure of a metal–organic cage functioning as tosylate-responsive channel.

The group of Nitschke tested the channel-forming ability of a metal–organic cage.^266^ They had found earlier that in the solid-state the cage Zn_10_(**97a**)_15_, shown in Figure 45, contains a halide ion in its central cavity, which is held in place by multiple aromatic C–H hydrogen bonds.^267,268^ Furthermore, in a different crystal structure, two tosylate anions were bound above and below that halide ion, giving the appearance of a blocked channel. To investigated whether such a cage can indeed function as anion channel, and if blockage by larger anions is possible, a cage comprised of ligand **97b** was created, which contains long alkyl chains to enhance lipophilicity and span the bilayer thickness. Ionic current measurements with Zn_10_(**97b**)_15_ inserted into a planar lipid bilayer indeed indicated channel formation and in an HPTS assay, selectivity for halide ions (Cl^–^, Br^–^, I^–^) over larger anions (e.g., NO_3_^–^, ClO_4_^–^) was demonstrated (EC_50_ = 5.02 μM for Cl^–^ transport). By changing the countercation from Na^+^ to Li^+^, K^+^, or Rb^+^, no changes in the rate of transport were noted, verifying selectivity for anions. The mediation of chloride transport was further confirmed in a lucigenin assay. To study potential blockage of the channel by larger anions, dodecyl sulfate was used, as by UV–vis titrations in octanol it showed a higher binding affinity (*K*_a_ = 1.02 × 10^5^ M^–1^) than the tosylate anion (*K*_a_ = 4.8 × 10^4^ M^–1^), and it is better soluble in the hydrophobic bilayer. As anticipated, addition of this anion diminished the transport activity as observed in a lucigenin assay, and closing of the channel was corroborated by ionic current recordings in the planar lipid bilayer experiments.

### 4.3 Cavitand-Based Channels

**Figure 46:**
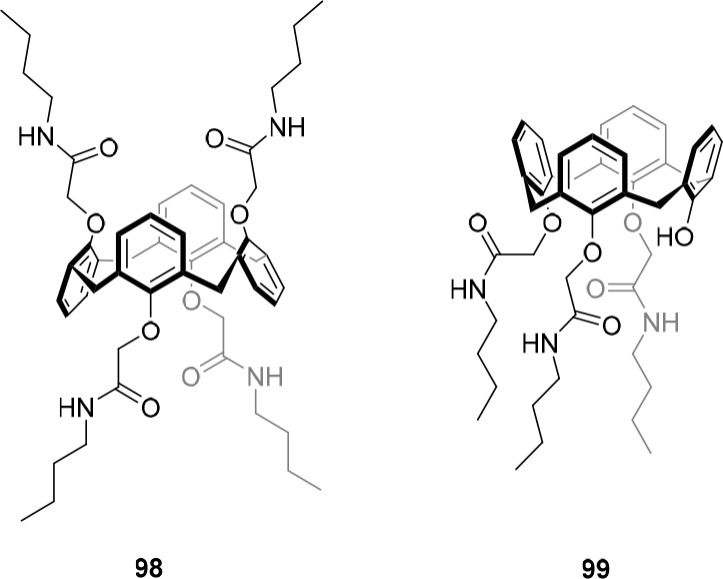
Calix[4]arene-based anion channels.

Among the first to demonstrate pH-controlled anion transport was the group of Davis.^269^ They discovered that tetrabutyl amide-functionalized calix[4]arene **98**, fixed in the 1,3-*alternate* conformation, is able to form channels that mediate passage of chloride ions.^270^ Later, they found that the *cone* conformer is also active, and that, when one of the *n*-butyl amide groups is replaced for an alcohol (**99**, Figure 46), pH-dependent transport can be observed. Calix[4]arene **99** bound chloride weakly as determined by a ^1^H NMR titration experiment in CD_2_Cl_2_ (*K*_a_ = 24 M^–1^). Transport of chloride was monitored using a lucigenin assay, where the activity decreased with more basic extravesicular solution (pH range 6.4–9.0). It was reasoned that by deprotonation of the phenolic OH, the resulting negative charge would inhibit chloride binding via charge repulsion, while also membrane permeability would be affected, both explaining the diminished transport observed at higher pH values.

The group of Gin developed an anion-selective β-cyclodextrin channel functionalized on the primary face with oligoether tails connected through amine linkages.^271^ Upon insertion in the membrane, a pore is created through which various ions can flow. The rates of transport were determined using an HPTS assay and, although these were similar for the cations tested (Na^+^, K^+^, and Li^+^), Br^–^ and I^–^ transport were faster (2.7 and 9.5 times, respectively) than that of Cl^–^, which in turn showed a rate similar to that determined for Na^+^ ions. The authors suggested that partial protonation of the amine linkages would favor anion transport through electrostatic interactions. It was indeed found later that, at a higher pH value at which the amines are not protonated, there was no selectivity for anion over cation transport.^272^

**Figure 47:**
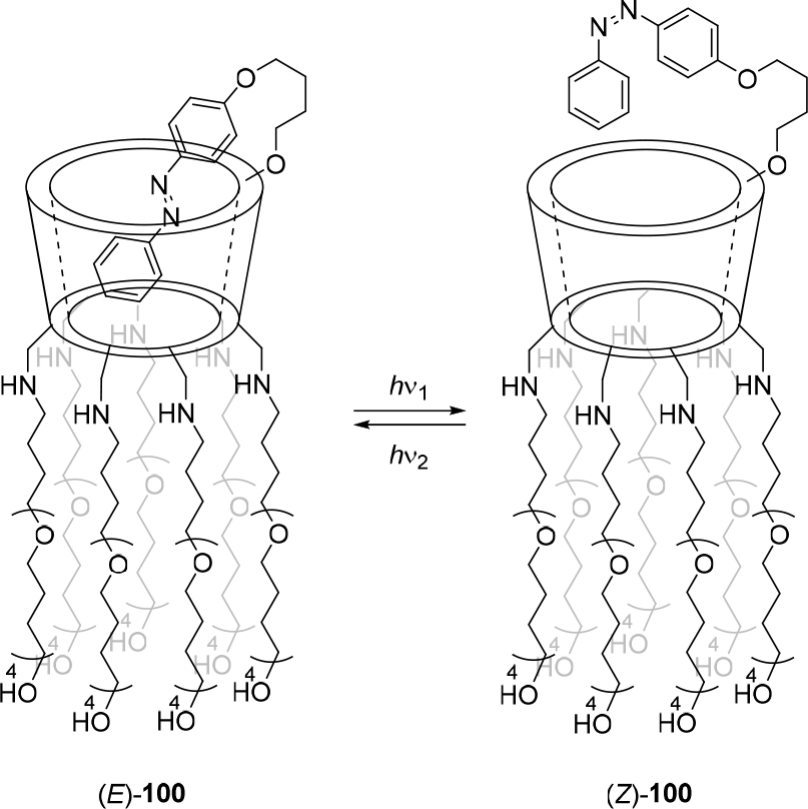
β-Cyclodextrin-based photoresponsive channel.

The fact that *E*-azobenzene is able to bind within the hydrophobic cavity of β-cyclodextrin with greater affinity than its *Z*-isomer^273^ offered a unique opportunity to open and close the channel by light. Therefore, the variant **100** with azobenzene tethered to the secondary face of the β-cyclodextrin unit was synthesized (Figure 47),^274^ and formation of an intramolecular inclusion complex was confirmed by ^1^H NMR and UV–vis studies. While by using an HPTS assay, it was established that small cations can pass the channel nonselectively in both the “open” and “closed” state, the overall rate of transport was surprisingly higher in the latter case. A possible explanation is that cation−π interactions with the azobenzene aid in the passage of cations. In contrast, the rate of anion transport reduced by blocking the pore, where the biggest difference was observed for Cl^–^ showing a 2.5-fold decrease for the *Z*-isomer with respect to the *E*-isomer. With this, it was demonstrated for the first time that blocking of a channel by a photoresponsive moiety is a viable strategy to gain control over transport.

### 4.4 Summary

As channels are membrane-spanning structures, control over transport can be achieved by other means than for carrier molecules. Most commonly, their formation has been controlled through stimuli-induced self-assembly of anion recognition motifs in the lipid bilayer, for example by applying a voltage, a change in pH, addition of a molecular effector, or reduction with glutathione. In particular, channel assembly activated by pH-change or glutathione is interesting for biological application because these are frequently encountered endogenous stimuli. Further, blockage of a channel constructed from a metal–organic cage was achieved using a strongly binding guest, whereas deprotonation or the formation of an inclusion complex with a photoswitchable azobenzene guest was used to control anion passage through cavitands.

## 5 Conclusions and Outlook

We have given a comprehensive overview and discussion of the strategies that have been developed so far to control binding as well as transmembrane transport of anions. The first reports describing responsive anion binding were mostly based on allosteric modulation by (alkali) metal cations, while ion-pairing interactions may have played an additional role in the observed affinity enhancements. Notably, too strong binding could impede reversibility of the binding process and therefore hamper potential application. Apart from that, the generation of chemical waste can be seen as a disadvantage of chemically responsive systems. The use of light as a stimulus seems to be ideal, as it is noninvasive and can be applied with high spatiotemporal precision.^22−27^ Indeed, over the past decade, there has been a surge in the development of receptors that are responsive to light. From the perspective of biological applications, pH- and redox-responsive systems are particularly interesting as they could be triggered by endogenous stimuli in the body, e.g., tumor cells have acidic microenvironments and elevated levels of reducing agents.^50−53^ Redox control of anion binding has been achieved in only a very few cases though.

As a considerable number of (nonswitchable) anion receptors has been shown to be capable of facilitating membrane transport,^14−20^ logically, their stimuli-responsive counterparts are potential candidates to control this process. The majority of examples reported thus far is based on pH change, in most cases to modify the anion binding site by (de)protonation, and sometimes to induce conformational changes that distort the binding site, which seems to be more effective. Likewise, with photoresponsive systems the principal thought is that control of binding affinity will result in altered transport activity. Although many photoresponsive anion receptors have been developed successfully and the first example of photocontrolled transport was reported in 2014,^124^ only in the past few years this direction has started to develop more rapidly. Successful control of transport activity by light has been achieved using molecular tweezers based on stiff-stilbene and azobenzene, through binding site blocking via intramolecular hydrogen bonding in hydrazone-based receptors, and with photocleavable protecting groups. Importantly, it appears clearly from these examples that other factors than a change in binding affinity, such as altered membrane partitioning and mobility upon switching are just as important to control transport activity. At the moment, studies aimed at altering solubility in the membrane with the use of chemical stimuli are mostly based on cleavage of water-soluble protecting groups using agents that are overexpressed in tumor cells (e.g., glutathione). Nevertheless, the potential role of membrane solubility in the pH- and light-responsive systems that we discussed herein should not be neglected.

Another important finding is that certain compounds with anion recognition motifs are able to self-assemble into membrane-spanning channels. This self-assembly has been influenced by stimuli such as voltage- and pH-change, small molecules or metal ions, as well as reductants and macrocyclic hosts. A foldamer, metal–organic cage, and a molecular cavitands were also shown to act as stimuli-responsive channels. As photoresponsive foldamers and metal–organic cages have been prepared, these results encourage assessment of their transport behavior. The same holds for the vast amount of other responsive anion receptors highlighted in the first section of this review. Will hidden potential be uncovered or will they fail (or already have failed) to show transport activity?

By connecting the themes of stimuli-responsive anion receptors and transmembrane anion transport, we hope to have stimulated researchers that currently focus on the former to explore the potential of their designs in the latter. As a guidance, we have identified the following key questions and challenges that, in our opinion, are crucial to address for further advancement of the combined field of responsive anion binding and transmembrane transport.

### 5.1 How Do Binding and Transport Relate?

The development of stimuli-responsive anion receptors is focused on maximizing the binding affinity difference between addressable states. With regard to application in transport, a switch in binding affinity is presumed to translate into altered transport activity, however, a relation between those two parameters has not unequivocally been established. In the examples that are discussed in this review, the strongest binding form does usually show the highest activity in transport studies, but the activity difference found between states often cannot be explained by their binding properties alone. Moreover, the binding constants of distinct types of receptors do not always correlate to their transport activity, i.e., weakly binding receptors can be good transporters and *vice versa*. This means that factors other than binding affinity are at play in defining transport activity, which could also be influenced by the switching process.

### 5.2 Controlling Affinity or Deliverability?

One of the factors, other than binding affinity, that can influence anion transport capability is membrane partitioning or deliverability. Where this aspect has been exploited by cleaving water-solubilizing groups, it has been mostly neglected in pH- and light-responsive systems. Yet, stimulus-controlled structural changes that alter binding affinity could additionally give rise to changed membrane partitioning, either in advantageous or disadvantageous manner. To dissect solubility or deliverability issues from other variables, the activity can be assessed via preincorporation of the transporter in the lipid bilayer instead of postaddition from solution to vesicles. While more labor-intensive, it will give better information about the origin of “apparent” differences in transport activity between interconvertible states.

### 5.3 Is There Necessity for Dynamic Control?

Where most of the responsive receptors that have been developed can be switched reversibly between a high- and low-affinity state, a large portion of the compounds used in transport studies is irreversibly activated by a stimulus. Is there a need for reversible on/off switching of transport? An increase in activity at a certain pathological site having different pH profile or elevated concentration of reductant may be sufficient in targeted pharmacological treatment. Nevertheless, full control over the activity of a drug in space and time can be desired to avoid build-up of resistance and further diminish side effects.^22−27^ Light-based systems are ideal in this regard, although a current drawback is that most of the reported receptors rely on irradiation with high-energy UV light, which could cause material degradation and has low penetration depth in tissue. With recent developments in visible-light activation of photoswitches,^119,120^ however, this issue can be fairly easily addressed in the near future.

### 5.4 Redox Responsiveness in Transport

At the moment, examples of redox-switchable anion receptors are scarce, and they are operated by an externally applied voltage or addition of metal reagents. Such systems are difficult to apply in membrane transport because of practical limitations. In contrast, several transporters and channels that are activated by glutathione have been reported, which is a naturally occurring reducing agent. Interestingly, these systems have been used to target tumor cells, in which glutathione concentrations are elevated. As redox control thus provides opportunities toward the development of selective therapeutic agents, further research into the design and synthesis of anion receptors that can respond to biologically relevant reductants is desired.

### 5.5 From Passive to Active Transport

Various biological processes rely on active anion transport, i.e., to pump against a concentration gradient by consuming energy. Conversely, all currently existing responsive synthetic anion transport systems solely enable control of passive transport, i.e., diffusion down a concentration gradient. The creation of anion concentration gradients across membranes by active anion transport is a major challenge in the field and would be particularly interesting in terms of energy conversion as well as the development of synthetic life-like systems. We are confident that new insights and developments from the field of artificial molecular machines^275−279^ will help addressing this challenge in the future, which is an exciting prospect.

### 5.6 Therapeutic Application: Promise or Reality?

In the examples of stimulus-controlled anion receptors, transporters and channels that have been highlighted in this review article, the general focus is on understanding fundamental design principles. Only recently, antibacterial and anticancer properties of some of the compounds have been demonstrated *in vitro*. There is a large step to take from these fundamental studies to pharmaceutical application. The use of these systems as new tools to interrogate and interfere with biological systems seems more realistic on the short-term. For example, to control neuronal activity by light somewhat similar to photoactive proteins in the field of optogenetics,^280^ or more simply to gain insight into faulty transport-related diseases by temporarily turning them on/off. Nevertheless, site-specific antibacterial and anticancer agents could emerge from this research field. One of the most exciting longer-term goals is to develop fully autonomous artificial transport systems that are capable of taking over (defective) protein function, as it could open a whole new range of possibilities for disease treatment.
